# Xanthine oxidase inhibition and white matter hyperintensity progression following ischaemic stroke and transient ischaemic attack (XILO-FIST): a multicentre, double-blinded, randomised, placebo-controlled trial

**DOI:** 10.1016/j.eclinm.2023.101863

**Published:** 2023-02-16

**Authors:** Jesse Dawson, Michele Robertson, David Alexander Dickie, Phillip Bath, Kirsten Forbes, Terence Quinn, Niall M. Broomfield, Krishna Dani, Alex Doney, Graeme Houston, Kennedy R. Lees, Keith W. Muir, Allan Struthers, Matthew Walters, Mark Barber, Ajay Bhalla, Alan Cameron, Alexander Dyker, Paul Guyler, Ahamad Hassan, Mark T. Kearney, Breffni Keegan, Sekaran Lakshmanan, Mary Joan Macleod, Marc Randall, Louise Shaw, Ganesh Subramanian, David Werring, Alex McConnachie

**Affiliations:** a School of Cardiovascular and Metabolic Health, College of Medical, Veterinary & Life Sciences, University of Glasgow, Queen Elizabeth University Hospital, Glasgow, G51 4TF, UK; b Robertson Centre for Biostatistics, School of Health and Wellbeing, College of Medical, Veterinary & Life Sciences, University of Glasgow, Glasgow, G12 8QQ, UK; c DD Analytics Cubed Ltd, 73 Union Street, Greenock, Scotland, PA16 8BG, UK; d Stroke Trials Unit, Mental Health & Clinical Neuroscience, University of Nottingham, Nottingham, NG7 2UH, UK; e Department of Neuroradiology, Institute of Neurological Sciences, Queen Elizabeth University Hospital, 1345 Govan Road, Glasgow, G51 4TF, UK; f School of Cardiovascular and Metabolic Health, College of Medical, Veterinary & Life Sciences, University of Glasgow, Glasgow Royal Infirmary, Glasgow, UK; g Department of Clinical Psychology and Psychological Therapies, Norwich Medical School, University of East Anglia, NR4 7TJ, UK; h Department of Neurology, Institute of Neurological Sciences Glasgow, Queen Elizabeth University Hospital, 1345 Govan Road, Glasgow, G51 4TF, UK; i Medicine Monitoring Unit (MEMO), School of Medicine, University of Dundee. Ninewells Hospital, Dundee, DD1 9SY, UK; j Division of Imaging and Science Technology, School of Medicine, Ninewells Hospital, Dundee, DD1 9SY, UK; k School of Medicine, College of Medical, Veterinary & Life Sciences, University of Glasgow, Glasgow, G12 8QQ, UK; l School of Psychology and Neuroscience, College of Medical, Veterinary & Life Sciences, University of Glasgow, Queen Elizabeth University Hospital, Glasgow, G51 4TF, UK; m Division of Molecular and Clinical Medicine, University of Dundee, UK; n University Department of Stroke Care, University Hospital Monklands, Airdrie, ML6 OJS, UK; o Department of Stroke, Ageing and Health, Guy's and St Thomas NHS Foundation Trust, St Thomas' Hospital, Lambeth Palace Rd, London, SE1 7EH, UK; p Wolfson Unit of Clinical Pharmacology, Royal Victoria Infirmary, Newcastle Upon Tyne, UK; q Department of Stroke Medicine, Mid and South Essex University Hospitals Group, Southend University Hospital, Prittlewell Chase, Westcliff-on-Sea, Essex, SS0 0RY, UK; r Department of Neurology, Leeds General Infirmary, Leeds, UK; s Leeds Institute of Cardiovascular and Metabolic Medicine, The University of Leeds, Leeds, UK; t Department of Medicine, South West Acute Hospital, Enniskillen, BT74 6DN, UK; u Department of Stroke Medicine The Luton and Dunstable University Hospital, Bedfordshire, NHSFT, Lewsey Road, Luton, LU4 0DZ, UK; v Institute of Medical Sciences, University of Aberdeen, Aberdeen, UK; w Department of Neurology, Leeds Teaching Hospitals NHS Trust, Leeds, UK; x Department of Stroke Medicine, Royal United Hospital, Combe Park, Bath, BA1 3NG, UK; y Department of Stroke Medicine, Nottingham University Hospitals, Nottingham, NG5 1PB, UK; z Stroke Research Centre, UCL Queen Square Institute of Neurology, London, UK; aa Comprehensive Stroke Service, National Hospital for Neurology and Neurosurgery, Queen Square, University College Hospitals NHS Foundation Trust, London, UK

**Keywords:** Stroke, Allopurinol, Hypertension, White matter hyperintensities

## Abstract

**Background:**

People who experience an ischaemic stroke are at risk of recurrent vascular events, progression of cerebrovascular disease, and cognitive decline. We assessed whether allopurinol, a xanthine oxidase inhibitor, reduced white matter hyperintensity (WMH) progression and blood pressure (BP) following ischaemic stroke or transient ischaemic attack (TIA).

**Methods:**

In this multicentre, prospective, randomised, double-blinded, placebo-controlled trial conducted in 22 stroke units in the United Kingdom, we randomly assigned participants within 30-days of ischaemic stroke or TIA to receive oral allopurinol 300 mg twice daily or placebo for 104 weeks. All participants had brain MRI performed at baseline and week 104 and ambulatory blood pressure monitoring at baseline, week 4 and week 104. The primary outcome was the WMH Rotterdam Progression Score (RPS) at week 104. Analyses were by intention to treat. Participants who received at least one dose of allopurinol or placebo were included in the safety analysis. This trial is registered with ClinicalTrials.gov, NCT02122718.

**Findings:**

Between 25th May 2015 and the 29th November 2018, 464 participants were enrolled (232 per group). A total of 372 (189 with placebo and 183 with allopurinol) attended for week 104 MRI and were included in analysis of the primary outcome. The RPS at week 104 was 1.3 (SD 1.8) with allopurinol and 1.5 (SD 1.9) with placebo (between group difference −0.17, 95% CI −0.52 to 0.17, p = 0.33). Serious adverse events were reported in 73 (32%) participants with allopurinol and in 64 (28%) with placebo. There was one potentially treatment related death in the allopurinol group.

**Interpretation:**

Allopurinol use did not reduce WMH progression in people with recent ischaemic stroke or TIA and is unlikely to reduce the risk of stroke in unselected people.

**Funding:**

The 10.13039/501100000274British Heart Foundation and the 10.13039/501100000364UK Stroke Association.

**Research in context**

**Evidence before this study**

We searched PubMed and Embase between 1st October 2019 and October 31st 2022. These dates were chosen as we have recently published a systematic review which covered dates from inception to 30th September 2019. In people with stroke, allopurinol has been shown to reduce markers of inflammation, endothelial function and blood pressure in small studies. Meta-analysis of randomised trials suggests allopurinol may lower cardiovascular event rate in people with established cardiovascular disease but this was not confirmed for people with ischaemic heart disease in the recent Allopurinol versus usual care in UK patients with ischaemic heart disease (ALL-HEART) study.

**Added value of this study**

Our study confirms a small effect of allopurinol on blood pressure. However, this effect, or that of other putative modes of action, were insufficient to modify a marker of cerebral small vessel disease and recurrent stroke risk.

**Implications of all the available evidence**

Allopurinol did not reduce white matter hyperintensities and is unlikely to reduce risk of stroke or cognitive decline in unselected people with ischaemic stroke and transient ischaemic attack. Allopurinol has a small effect on blood pressure, which is unlikely to be important in unselected people, but may be larger in people with hyperuricaemia.

## Introduction

People who have an ischaemic stroke are at risk of cognitive decline and recurrent vascular events.^1^^,^^2^ Higher serum uric acid (UA) levels are associated with vascular cognitive impairment,^3^ increased risk of first and recurrent stroke^4^ and a worse outcome after ischaemic stroke.^5^ Mendelian randomisation studies demonstrate that higher genetically predicted serum uric acid level is associated with increased risk of coronary artery disease and ischaemic stroke and that this is, in part, mediated by the relationship between serum uric acid and blood pressure (BP).^6^ Allopurinol, the most used urate-lowering drug in people with gout, has been shown to reduce markers of inflammation,^7^ augmentation index, progression of carotid intima-media thickness and BP^8^ in people with stroke and to increase cerebral nitric oxide bioavailability in people with diabetes.^9^ Allopurinol also reduces blood pressure in hyperuricemic adolescents with hypertension and may have additional urate-independent effects.^10^ Meta-analysis of randomised trials suggests allopurinol may lower cardiovascular event rate in high-risk individuals.^6^

White matter hyperintensities of presumed vascular origin (WMH) are a marker of cerebral small vessel disease and are present in as many as 90% of people with ischaemic stroke.^11^ The degree of WMH burden and progression over time are associated with higher rates of stroke, death, and cognitive and physical decline.^12^ BP reduction may reduce WMH progression.^13^^,^^14^ We hypothesized that allopurinol may reduce WMH progression in people with recent stroke by lowering BP, and through additional effects on vascular stiffness and function. If this were the case, this would raise the possibility that allopurinol may reduce cognitive decline and stroke recurrence after stroke.

The Xanthine oxidase Inhibition for improvement of Long-term Outcomes Following Ischaemic Stroke and Transient ischaemic attack (XILO-FIST) trial aimed to determine whether allopurinol reduces WMH progression and BP in people with recent ischaemic stoke.

## Methods

### Study design

XILO-FIST was a multicentre, prospective, randomised, double-blinded, placebo-controlled trial performed in 22 sites in the UK. Further details regarding the design of the trial have been published previously and the protocol is available on-line.^15^ The study was approved by the NHS Research Ethics Committee (REC number 14/WS/0113) and by the UK Medicine and Health Regulatory Agency. Written informed consent was obtained from all participants. The study was conducted according to the Declaration of Helsinki 2013. The study reporting followed Consolidated Standards of Reporting Trials (CONSORT) guidance. The study included a cardiac Magnetic Resonance Imaging (MRI) sub-study, which is not reported here.

### Participants

Study participants were adults aged greater than 50 years with a history of ischaemic stroke or transient ischaemic attack (TIA) within the past 4 weeks. Potential participants were identified during in-patient stay in an acute stroke unit or in a cerebrovascular out-patient clinic. Diagnosis was confirmed by a stroke physician. All subtypes of ischaemic stroke and TIA were included. Full inclusion and exclusion criteria are provided in Appendix Table 1.

### Randomisation and masking

Participants were randomised (1:1) following completion of the run-in phase to receive either allopurinol or matching placebo orally for 104 weeks. Randomisation was carried out using a bespoke study web portal and was performed by the Robertson Centre for Biostatistics at the University of Glasgow. Twenty percent of participants were allocated to treatments by simple randomisation, with the remaining 80% allocated by a minimisation algorithm which included presence of WMH at baseline and cardiac sub-study eligibility as minimisation factors. Changes in serum uric acid concentration would have compromised allocation concealment so this was not measured as part of the study.

### Procedures

The study comprised a 4-week run in phase and a 104-week treatment phase. The run-in phase comprised an enrolment visit on day 0 and a baseline assessment visit at 4 weeks. In order to successfully complete the run-in phase and proceed to randomisation, participants must have completed baseline data collection and have undergone brain MRI. No study medication was given during the run-in phase.

A summary of study procedures is given in Appendix Table 2. The baseline assessment visit included assessment of brachial sphygmomanometer BP, ambulatory blood pressure monitoring (ABPM), electrocardiography, brain MRI, and assessment of cognitive function using the Montreal Cognitive Assessment (MoCA, version 7.3) and a multidomain neuropsychological battery.

After randomisation, participants were dispensed study medication and were followed up at weeks 4, 13, 26, 52, 78 and 104. At all visits, participants were assessed for adverse events, brachial BP was measured, safety blood tests were performed, and study medication was dispensed. In addition, ABPM was performed at the week 4 visit and a MoCA was performed at week 52. At the week 104 visit, measurement of brachial BP, ABPM, electrocardiography, brain MRI, and assessment of cognitive function were performed, and study medication was stopped. A telephone follow-up was performed one week later to assess for adverse events.

During the first 4 weeks after randomisation, a single 300 mg daily dose of oral allopurinol or placebo was prescribed. All participants underwent dose titration to allopurinol 300 mg twice daily or placebo unless estimated Glomerular Filtration Rate (eGFR) was <60 mL/min, where once daily dosing was continued. The total treatment duration was 104 weeks. Dose modification (a reduction from 300 mg twice daily to 300 mg once daily) occurred if renal function declined to an eGFR of <50 mL/min or in the event of side effects. Dosing was stopped if renal function declined to an eGFR of <30 mL/min.

Brain MRI was performed using 1.5 or 3 T MRI. The protocol required the same MRI scanner and same sequence parameters to be used for the baseline and follow-up scans. Study sequences include T1 weighted imaging, T2-weighted imaging, fluid attenuated inversion recovery (FLAIR), diffusion weighted imaging and susceptibility weighted imaging. Isotropic T1, T2 and FLAIR imaging were performed where possible. Typical sequence parameters are given in Appendix Table 3.

All scans were reviewed blinded to treatment allocation. The Standards for Reporting Vascular changes on Neuroimaging (STRIVE) recommendations were followed during image review.^16^ Accordingly, WMH of presumed vascular origin were defined as hyperintense lesions on FLAIR in the white matter that were not due to the index stroke, did not have a hyperintense rim, and were not confluent with areas of cortical infarction. All visual rating scales were assessed independently by two trained observers (JD, KD, or DD). Where there was any level of disagreement on a score, this was resolved in an adjudication meeting between the two reviewers and a consensus score applied.

Fazekas and Scheltens scale scores were calculated for each baseline and week 104 scan.^17^^,^^18^ A Rotterdam progression score (RPS) and Schmidt's progression score were calculated by simultaneous side by side review of the baseline and week 104 scans.^19^^,^^20^ This was done with random ordering of baseline and follow-up scans. Once review was complete, ordering was un-blinded to allow determination of progression score.

Volumetric assessment of WMH volume was performed. First, the white matter volume was estimated using atlas-based segmentation.^21^ A probability map of white matter created from 313 volunteers aged 18–96 years was used^22^ and registered to each scan using non-linear (diffeomorphic) registration to provide an initial estimate of white matter in each participant.^23^^,^^24^ Hyperintense outliers were identified on FLAIR by transforming each voxel to a standard (z) score. Voxels with z ≥ 1.5 and within the estimated white matter volume were initially defined as WMH. Final WMH estimates were defined by 3D Gaussian smoothing to reduce noise and account for partial volumes around WMH edges. Automatic WMH estimates were visually checked, and infarcts masked by a trained image analyst following STRIVE guidelines.^16^

New brain infarction was assessed by side-to-side review of baseline and follow-up MRI scans by 1 reviewer (JD). FLAIR and DWI images were reviewed. Areas of new cortical infarction or new lacunar infarction were classed as new brain infarction.

Twenty four hour ABPM was performed at baseline, week 4 and week 104 unless contraindicated. A Spacelabs Ultralight Ambulatory Blood Pressure Monitor was used. This was set to take readings every 30 minutes during daytime (0800 h–2159 h) and every 60 minutes during night-time (2200 h–0759 h). ABPM was not performed in participants with significant arm weakness who would be unable to remove the device in the event of discomfort or other problems.

### Outcomes

The primary outcome was WMH progression measured using the RPS. Secondary outcomes were Schmidt's progression score, change in WMH volume at week 104, change in Fazekas' score at week 104, change in Scheltens' score at week 104, new brain infarction at week 104 MRI, RPS with those who died/became too frail to undergo repeat imaging assigned worst score, change in mean day-time systolic BP (SBP) at week 4, change in mean day-time diastolic BP (DBP) at week 4, change in mean day-time SBP at week 104, change in mean day-time DBP at week 104 and change in MoCA score. We also assessed in-clinic BP at week 4 and week 104 as an exploratory outcome.

### Protocol amendments and additional changes due to the COVID-19 pandemic

A summary of all protocol amendments is given in Appendix Table 4. On the 23rd of March 2020 the government in the United Kingdom issued a stay-at-home order in response to the COVID-19 pandemic. All participating sites suspended clinical research activity, unless it was related to COVID-19 or unless there was a specific participant safety issue. Several sites implemented similar changes in the weeks prior to this date. At this point, enrolment and visits up to week 52 had completed but there were 90 participants still under follow-up. An amendment was approved to allow participants to continue study medication for a maximum of an additional 6-months in the hope that follow-up could be completed after the first wave of infections had passed. The amendment also allowed for telephone visits to be conducted to obtain study data if a face-to-face visit was not possible. In addition, ABPM was no longer performed at the week 104 visit.

### Statistical analysis

We assumed that 90% of participants would have evidence of WMH at baseline and that approximately 64% would progress by one point or more on the RPS and that the mean progression score in the placebo group would be 1.293 at week 104 based on data from the Leukoaraiosis and Disability Study.^25^ We calculated, based on a Wilcoxon-Mann-Whitney test, that a sample size of 192 participants per group would give 80% power to detect a 30% relative reduction in progression score at a 5% significance level (nQuery Advisor® v7.0). This was chosen as a conservative minimally important difference as it is less than the previously reported difference seen with BP reduction.^26^ Further detail on the assumptions used of the sample size calculation are contained in the study protocol. We planned to randomize 232 participants per group to allow for withdrawals and for participants who would be unable to return for the week 104 MRI. We also calculated that 101 participants per group would be required to give 80% power at a 5% significance level to detect the previously reported 3.3 mmHg difference in change in SBP^26^ (assumed SD 8.3) at week 4.

All analyses were carried out according to the intention-to-treat (ITT) principle. Additional analyses were to be carried out using a per-protocol (PP) population. This excluded participants where there was an eligibility violation, participants who had more than 90 days of total treatment interruption and participants from one site where a serious breach of good clinical practice (GCP) was detected. The safety analysis set included all participants who received at least one dose of study medication.

The primary outcome was assessed by a linear regression model which adjusted for minimisation variables, site (as a random factor), and baseline characteristics associated with WMH progression (age, baseline National Institute of Health Stroke Scale score, baseline clinical SBP and Scheltens total score). Secondary outcomes were assessed by the same method except for progression on Schmidt's Progression score and presence of new brain infarction which were analysed by a Chi-squared test and logistic regression to adjust for minimisation variables. A p value of <0.05 was used for statistical significance. We pre-specified three sub-group analyses. These were by age, baseline uric acid level defined by the median and whether participation was completed before the introduction of Covid restrictions. We also performed a sensitivity analysis for MRI outcomes which included only those participants who had baseline and week 104 imaging performed on the same scanner, with the same sequence parameters, and no other quality issues deemed to affect interpretation.

The trial is registered in clinicaltrials.gov (registration number NCT02122718) and was adopted by the UK National Institute of Health Stroke Research Network and the Scottish Stroke Research Network.

The trial was overseen by a Trial Steering Committee (TSC) which met at least annually and comprised an independent chair, three other independent members, a participant representative, the Chief Investigator, and trial statistician. An independent Data Monitoring Committee (IDMC) met at least annually to review unblinded data. This comprised 4 independent members. The day-to-day running of the trial was overseen by the Trial Management Group at the University of Glasgow chaired by the Chief Investigator. Details of committee members are given in Appendix X.

### Role of the funding source

The funder of the study had no role in study design, data collection, data analysis, data interpretation, or writing of the report. JD, AM and MR have had access to all study data. JD had final responsibility for the submission of the manuscript. All authors agreed to submission.

## Results

**Fig. 1:**
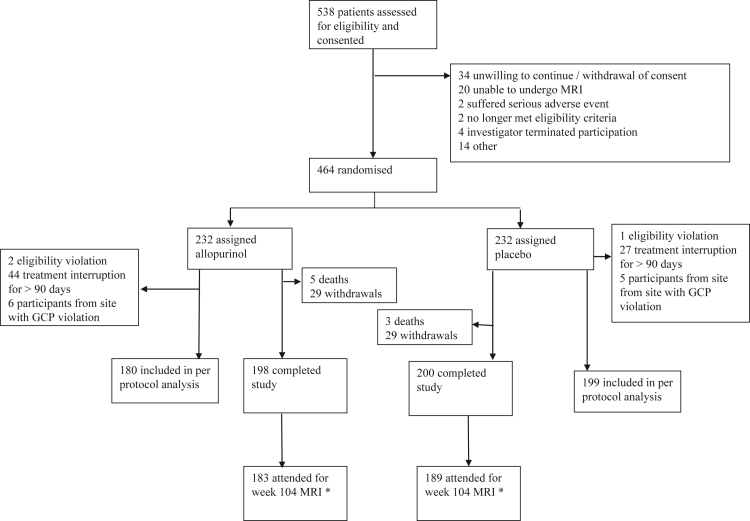
**Trial profile**. Figure shows the number of participants who were eligible for participation and gave consent followed by details on randomisation, follow-up and withdrawal. ∗Included in assessment of the primary outcome. MRI = magnetic resonance imaging. GCP = good clinical practice.

A total of 538 participants were consented and entered the run-in phase between 25th May 2015 and the 29th November 2018. Of these, 74 withdrew prior to randomisation, leaving 464 participants who were randomised (232 per group). Of these, 372 (189 with placebo and 183 with allopurinol) attended for week 104 MRI (see trial profile, Fig. 1). There were no significant protocol deviations that affected the rights, safety, or well-being of participants or the scientific integrity of the study with the exception of at one site which enrolled fewer than 20 participants. This site was found to be in serious breach of GCP, which was reported to UK Medicine and Health Regulatory Agency. No participant came to harm from this breach. The safety analysis set included 460 participants who received at least one dose of medication. The per protocol analysis set included 379 participants. Reasons for exclusion from the per-protocol population were eligibility violation (n = 3), treatment interruption for more than 90 days (n = 71) and enrolment from the site with a serious breach of GCP. The trial was subject to a routine inspection by the UK Medicine and Health Regulatory Agency in January 2019. There were no critical findings.

Baseline demographics are shown in Table 1. Baseline measures of WMH are shown in Table 2. Groups were well matched at baseline. Enrollment by site is shown in Appendix Table 5.

**Table 1:** Baseline characteristics.

	Allopurinol (n = 232)	Placebo (n = 232)
Age years, mean (SD)	65.8 (8.9)	65.6 (8.6)
Male gender, n (%)	154 (66.4%)	165 (71.1%)
Female gender, n (%)	78 (33.6%)	67 (28.9%)
Ethnicity, n (%)		
White	231 (99.6%)	228 (98.3%)
Multiple/mixed ethnic	0	1 (0.4%)
Black	1 (0.4%)	2 (0.9%)
Other	0	1 (0.4%)
Current smoker, n (%)	47 (20.3%)	48 (20.7%)
Body mass index, mean (SD)	28.1 (4.7%)	28.8 (5.4%)
Systolic blood pressure, mmHg, mean (SD)	136.0 (17.7)	136.6 (17.2)
Diastolic blood pressure, mmHg, mean (SD)	78.5 (10.6)	79.9 (10.4)
Myocardial infarction, n (%)	20 (8.6%)	21 (9.1%)
Stroke, n (%)	17 (7.3%)	24 (10.3%)
Transient ischaemic attack, n (%)	21 (9.1%)	25 (10.8%)
Peripheral vascular disease, n (%)	12 (5.2%)	15 (6.5%)
Hypertension, n (%)	121 (52.2%)	122 (52.6%)
Diabetes, n (%)	48 (20.7%)	51 (22.0%)
Dyslipidemia, n (%)	83 (35.8%)	80 (34.5%)
Gout, n (%)^a^	4 (1.8%)	1 (0.4%)
Qualifying event, n (%)		
Ischemic stroke	215 (92.7%)	217 (93.5%)
Transient ischaemic attack	17 (7.3%)	15 (6.5%)
Time from index event to randomization, days mean (SD)	41.2 (11.6)	41.5 (9.4)
Stroke class, n (%)		
Total anterior circulation stroke	7 (3.0%)	7 (3.0%)
Partial anterior circulation stroke	68 (29.3%)	66 (28.4%)
Lacunar stroke	103 (44.4%)	91 (39.2%)
Posterior circulation stroke	54 (23.3%)	67 (28.9%)
Amaurosis fugax	0	1 (0.4%)
Lipid lowering therapy, n (%)	226 (97.4%)	225 (97.0%)
Antithrombotic therapy, n (%)	227 (97.8%)	226 (97.4%)
Blood pressure lowering therapy, n (%)	205 (88.4%)	205 (88.4%)
National Institute of Health Stroke Scale score, mean (SD)	1.4 (1.7)	1.6 (2.0)
Modified Rankin Scale Score of 0–1	128 (55.2%)	134 (57.7%)
Estimated Glomerular Filtration Rate, ml/min/1.73 m^2^, mean (SD)	85.5 (19.8)	86.3 (19.8)
Serum uric acid, μmol/l, mean (SD)	342.2 (84.2)	328.5 (87.5)

a Gout data were only reported on n = 228 with allopurinol and 229 with placebo. SD = standard deviation.

**Table 2:** Baseline measures of white matter hyperintensities.

	Allopurinol (n = 232)	Placebo (n = 232)
Fazekas total score, mean (SD)	2.7 (1.2)	2.7 (1.3)
Scheltens total score, mean (SD)	12.5 (6.0)	12.7 (7.1)
White matter hyperintensity volume, mls, mean (SD)	16.7 (15.0)	18.2 (18.8)
Fazekas periventricular hyperintensities score, n (%)		
Caps or pencil thin lining	161 (69.4%)	158 (68.1%)
Smooth halo around ventricles	45 (19.4%)	50 (21.6%)
Irregular halo	26 (11.2%)	24 (10.3%)
Fazekas deep white matter score n (%)		
Absent	8 (3.4%)	11 (4.7%)
Multiple focal lesions	163 (70.3%)	154 (66.4%)
Early confluent lesions	48 (20.7%)	48 (20.7%)
Confluent lesions	13 (5.6%)	19 (8.2%)
Scheltens periventricular hyperintensity score, mean (SD)	3.6 (1.1)	3.7 (1.2)
Scheltens white matter hyperintensity score, mean (SD)	6.7 (4.3)	6.5 (4.4)
Scheltens basal ganglia score, mean (SD)	0.9 (1.1)	1.1 (1.5)
Scheltens infratentorial fossa score, mean (SD)	1.3 (1.4)	1.4 (1.7)

SD = standard deviation.

A total of 83 participants stopped study medication (52 (22.4%) with allopurinol and 31 (13.4%) with placebo). This was due to a related adverse event in 33 treated with allopurinol and 21 with placebo. The median time (IQR) to treatment discontinuation was 181 (54–368) days with allopurinol and 109 (43–208) days with placebo. The mean change in serum uric acid level at week 104 was - 92.6 μmol/l (SD 135.1) in the allopurinol group and - 13.8 μmol/l (SD 101.4) in the placebo group.

Four sites installed new MRI scanners during the study period meaning that their week 104 scans were performed on a different scanner to the baseline scan (n = 32).

The RPS was 1.3 (SD 1.8) with allopurinol and 1.5 (SD 1.9) with placebo, between group difference −0.17, 95% CI −0.52 to 0.17, p = 0.33. There was no significant difference in the Schmidt's Progression Score, in WMH volume, or in the RPS where those who died or became too frail to undergo MRI were assigned the highest score (Table 3). There was also no difference in the odds of new brain infarction at week 104 (Table 3). There was no significant difference in change in Fazekas' score but change in Scheltens' score was lower with allopurinol (Table 3).

**Table 3:** Imaging outcomes.

Outcome	Change/n (%) with allopurinol	Change/n (%) with placebo	Between group difference	95% CI	P value
RPS score, mean (SD)	1.3 (1.8)	1.5 (1.9)	−0.17	−0.52 to 0.17	0.33
WMH volume (log), mean (SD)	0.1 (0.3)	0.1 (0.3)	0.01	−0.04 to 0.07	0.61
Schmidt's progression score, n (%)	107 (58.5%)	107 (56.6%)	OR 1.08	0.72–1.63	0.72
Schelten's score, mean (SD)	0.8 (2.8)	1.3 (3.1)	−0.68	−1.28 to −0.08	0.026
Fazekas score, mean (SD)	0.0 (0.6)	0.1 (0.7)	−0.07	−0.19 to 0.06	0.29
New infarction, n (%)	28 (15.3%)	23 (12.4%)	OR 1.31	0.72–2.37	0.38

CI = confidence interval. RPS = Rotterdam progression scale. WMH = white matter hyperintensity. A negative value denotes a change in favour of allopurinol. The sample size for these analyses was n = 189 for placebo and n = 183 for allopurinol. OR = odds ratio for progression. All reported outcomes are adjusted for site, age, baseline National Institute of Health Stroke Scale score, baseline clinical systolic blood pressure and Schelten's total score. SD = standard deviation.

At week 4 SBP fell with allopurinol (−2.4 mmHg 95% CI 95% CI −4.0 to −0.8, p = 0.0029) but was unchanged with placebo (0.9 mmHg, 95% CI −0.6 to 2.4, p = 0.24). The between-group difference was −3.3 mmHg (negative value in favor of allopurinol, 95% CI −5.5 to −1.1, p = 0.0034). The change in daytime SBP at week 104 was similar but the difference was not significant. There was no significant difference in daytime DBP at week 4 or week 104 (Table 4).

**Table 4:** BP outcomes.

Outcome	Change with Allopurinol	Change with Placebo	Between group difference	95% CI	P value
ABPM SBP week 4, mean (SD)	−2.3 (12.9)	0.8 (10.7)	−3.33	−5.55 to −1.11	0.0034
ABPM SBP week 104, mean (SD)	−2.3 (14.2)	0.1 (13.4)	−2.95	−6.0 to 0.10	0.058
ABPM DBP week 4, mean (SD)	−1.2 (7.4)	−0.3 (5.6)	−1.17	−2.47 to 0.13	0.076
ABPM DBP week 104, mean (SD)	−1.3 (8.5)	−1.1 (8.5)	−0.84	−2.65 to 0.96	0.36

CI = confidence interval. ABPM = ambulatory blood pressure monitor. SD = standard deviation. SBP = systolic blood pressure. DBP = diastolic blood pressure. A negative value for the between group difference denotes a change in favour of allopurinol. The sample size for the ABPM analyses was n = 182 for placebo and n = 170 for allopurinol at week 4 and n = 126 for placebo and n = 109 for allopurinol at week 104. All reported outcomes are adjusted for site, age, baseline National Institute of Health Stroke Scale score, baseline clinical systolic blood pressure and Schelten's total score.

The change in MOCA score at week 104 was 0.2 (SD 2.7) with allopurinol and 0.4 (SD 2.8) with placebo, between group difference 0.00, 95% CI −0.49 to 0.49, p = 0.99.

Findings were consistent for all primary and secondary endpoints in the per protocol analysis and the sensitivity analyses (Appendix Table 6). Results were consistent for all outcomes across the age, pre or post COVID-19 and uric acid sub-groups subgroups with no significant treatment interactions (Appendix Table 7). Although the treatment interaction as not significant, there was no significant difference in the week 4 change in SBP in people with baseline uric acid below the median level but there was a significant difference in favor of allopurinol in people with baseline uric acid above the median level (between group difference −4.9 mmHg, 95% CI −8.8 to −1.0, p = 0.014) (Appendix Table 7).

No suspected unexpected serious adverse reactions were reported. A total of 239 serious adverse events (127 with allopurinol, 112 with placebo) were reported in 137 (30%) participants (73 (32%) with allopurinol and 64 (28%) with placebo) (Table 5). There was one fatal serious adverse event of aplastic anemia in a participant randomised to allopurinol. Eleven participants had a suspected drug rash with allopurinol and 3 with placebo. None of the rash events were reported as serious. There were four SAEs reported as possibly or probably related to study drug in the allopurinol group. There were 5 deaths in allopurinol treated participants (three cardiovascular and two non-cardiovascular) and three deaths in placebo treated participants (two cardiovascular and one non-cardiovascular).

**Table 5:** Safety data.

Serious adverse event	All participants (n = 460)	Allopurinol (n = 231)	Placebo (n = 229)
At least one SAE	137 (29.8%)	73 (31.6%)	64 (28.0%)
Nervous system disorders	51 (11.1%)	31 (13.4%)	20 (8.7%)
Infections and infestations	29 (6.3%)	14 (6.1%)	15 (6.6%)
Cardiac disorders	23 (5%)	10 (4.3%)	13 (5.7%)
Injury, poisoning and procedural complications	14 (3.0%)	7 (3.0%)	7 (3.1%)
Neoplasms, benign, malignant and unspecified	12 (2.6%)	8 (3.5%)	4 (1.7%)
Surgical and medical procedures	11 (2.4%)	4 (1.7%)	7 (3.1%)
Vascular disorders	11 (2.4%)	6 (2.6%)	5 (2.2%)
Gastrointestinal disorders	9 (2.0%)	4 (1.7%)	5 (2.2%)
Renal and urinary disorders	8 (1.7%)	6 (2.6%)	2 (0.9%)
Metabolism and nutrition disorders	7 (1.5%)	2 (0.9%)	5 (2.2%)
Respiratory, thoracic and mediastinal disorders	7 (1.5%)	2 (0.9%)	5 (2.2%)
General disorders and administration site conditions	6 (1.3%)	4 (1.7%)	2 (0.9%)
Musculoskeletal and connective tissue disorders	5 (1.1%)	3 (1.3%)	2 (0.9%)
Psychiatric disorders	5 (1.1%)	4 (1.7%)	1 (0.4%)
Hepatobiliary disorders	3 (0.7%)	1 (0.4%)	2 (0.9%)
Skin and subcutaneous tissue disorders	2 (0.4%)	2 (0.9%)	0
Blood and lymphatic system disorders	1 (0.2%)	1 (0.4%)	0
Ear and labyrinth disorders	1 (0.2%)	1 (0.4%)	0
Eye disorders	1 (0.2%)	1 (0.4%)	0
Investigations	1 (0.2%)	1 (0.4%)	0

Adverse events in each MedDRA system.

All values are n (%). SAE = serious adverse event.

## Discussion

Two years of allopurinol treatment did not reduce progression of brain WMH when initiated within 4 weeks of ischaemic stroke or TIA in people aged greater than 50 years. Systolic BP was lower following 4 weeks of allopurinol treatment and although the between group difference in systolic BP was not statistically significantly different at week 104, it was of similar magnitude and this may reflect type two error.

The change in WMH volume in our study was similar to that seen in other studies which included people with stroke.^13^^,^^27^ In the PROGRESS MRI sub-study,^13^ SBP was 11.2 mmHg lower and WMH volume 1.6 cm^3^ lower with BP treatment. In the PRoFESS MRI substudy,^27^ there was no difference in WMH volume with BP treatment but the between group difference in SBP was 3 mmHg. Studies in other populations consistently show a reduction in WMH volume in favour of intensive BP control and there is a strong relationship between the intergroup BP difference and the difference in change in WMH volume^28^ during prospective follow up. It is likely that the BP difference obtained in our study was insufficient to lead to a difference in WMH volume over a two-year period of follow up.

Allopurinol is reported to have effects on the cardiovascular system which are independent of BP reduction, and which could be associated with WMH progression.^29^ However, our data support neither a BP dependent nor independent effect of allopurinol on WMH. There was a statistically significant difference in the change in Schelten's score. However, this difference was not apparent in any other measure, including volumetric analysis, so may be a chance finding. While many studies show potentially beneficial effects of xanthine oxidase inhibition on the cardiovascular system, it is important to note that the xanthine oxidase system is part of a complex pro-oxidant and anti-oxidant system. Xanthine oxidase inhibition may inhibit reduction of nitrite and nitrate back to NO.^30^ In addition, metabolism of allopurinol to oxypurinol, which occurs rapidly in the plasma, can generate hydrogen peroxide and oxidative stress.

The recently reported Allopurinol versus usual care in UK patients with ischaemic heart disease (ALL-HEART) study found no difference in the rate of a composite primary outcome of non-fatal myocardial infarction, non-fatal stroke, or cardiovascular death (or in any secondary outcome) in approximately 6000 participants with ischaemic heart disease.^31^ There was also no suggestion of benefit in people in the highest tertile of serum uric acid levels.

The observed reduction in BP following allopurinol treatment was small but was greater in people with higher baseline serum uric acid. Broadly this is consistent with results of a recent systematic review and meta-analysis of randomised trials,^6^ and results of studies in hyperuricemic adolescents where large reductions in BP were seen with allopurinol.^10^^,^^32^ However, it is in contrast to the recent cross-over SURPHER trial,^33^ where no difference between allopurinol or placebo in 24-h average SBP or ABPM was seen. One important difference with the SURPHER trial is that we used a higher dose of 300 mg twice daily, which has been shown to have a greater effect on endothelial function.^34^ Meta-regression analysis suggests a greater fall in BP with a higher baseline serum UA level and it is likely that older adults with prolonged hyperuricemia become insensitive to the large effect of UA reduction seen in younger adults.^6^ This means it is likely that high doses of allopurinol are needed to see even a small BP effect. Previous studies may have been underpowered to detect small differences in BP, particularly if baseline serum UA is low, participants are well treated with other medications, and lower doses of allopurinol are used. We believe the most informative measure of the effect of allopurinol on BP is the week 4 change. This is because BP treatment is highly likely to be modified to achieve BP targets in the longer-term, which could mask any effect of allopurinol on BP. This is less likely to confound change at earlier timepoints. This may partly explain why the two-year between group difference in change in SBP did not reach statistical significance, although it was similar to the week 4 data. This may also reflect less precision due to a lower sample size for the week 104 analysis, due to the Covid pandemic, and the greater standard deviation for the change at week 104.

Allopurinol has important potential side effects. We saw adverse events at a rate in keeping with other secondary prevention trials. The study had an independent data monitoring committee who regularly reviewed all efficacy and safety data. Importantly, the number of serious adverse events reported to be possibly or probably related to study drug were in the allopurinol group was 4.

The strengths of our study include blinding to treatment allocation, use of central randomisation and wide inclusion criteria which should increase generalizability. The trial was rigorously monitored and was subject to a routine regulatory inspection by the regulatory authority. We also used a high dose of allopurinol.

Our trial has limitations. We anticipated that some participants would be unable to attend follow up due to death or illness. Our sample size calculation was based on 384 participants having a 2-year MRI performed but only 372 attended. Unfortunately, 12 participants did not attend for final follow up and we were unable to perform ABPM in 90 people at the week 104 visit as a direct consequence of the Covid pandemic. In addition, 52 participants in the allopurinol group stopped taking study medication. This withdrawal rate from treatment rate of 22.4% in allopurinol treated participants is in line with other secondary prevention trials. In the Insulin Resistnace Intervention Trial of pioglitazone use in people with stroke or TIA and insulin resistance, it was 40%.^35^ In the Effects of fluoxetine on functional outcomes after acute stroke (FOCUS) trial, approximately 2/3 of participants took medication for 150 days or more.^36^ Although this will reduce study power, it is unlikely this led to type 2 error given the absence of any evidence of a between group difference in WMH volume and no suggestion of important differences in the per-protocol and sensitivity analyses. Our study included a majority of male and Caucasian participants. In addition we included a heterogenous sample of people with ischaemic stroke and TIA and we did not specifically select people with small vessel disease or by degree of white matter hyperintensity burden. We did not adjust for multiple testing on assessment of our secondary endpoints.

In the XILO-FIST trial we found no evidence of an effect of allopurinol on white matter hyperintensity progression or on new brain infarction but a small reduction in SBP at 4 weeks, which was broadly sustained at 2 years. The change in BP with allopurinol may be greatest in people with higher serum uric acid levels and further study should aim to assess the clinical importance of this in people with stroke. It is unlikely it will reduce progression of white matter hyperintensities in a clinically important way in an unselected population of people with ischaemic stroke or TIA.

## Contributors

JD, NB, MRW, KD, KM, KL, AD, GH, SK, AM, TQ, MK, AS were involved in design of the study. MR and AM performed statistical analysis. JD, DD, KF, KD, TQ, MB, DW, AB, AD, AC, AH, MM, BK, AB, LS, GS, PG and SL acquired study data. JD drafted the manuscript. PB chaired the trial steering committee. All authors provided critical comment and contributed to the design of the study. JD, MR and AM have accessed and verified the underlying data.

## Data sharing statement

Study data, including brain MRI will be shared with the Virtual International Stroke Trials Archive after publication of the primary manuscript. Study data including anonymized individual level participant data will be shared with academic investigators or health care professionals following review and approval of a proposal and subject to a data sharing agreement (contact jesse.dawson@glasgow.ac.uk).

## Declaration of interests

JD has received honoraria from Pfizer, Daiichi Sankyo, Medtronic, Astra Zeneca, Bristol Myers Squibb, and Bayer unrelated to this trial.

PMB is Stroke Association Professor of Stroke Medicine and an Emeritus NIHR Senior Investigator. He has received consulting fees from CoMInd, DiaMedica, Roche and Phagenesis. He is co-chair of the World Stroke Organisation Industry Committee. He has received equipment for research studies from Phagenesis. He reports stock options in DiaMedica and CoMind and was a member of the Data Safety Monitoring Committee for the European Carotid Surgery Trial-2. All reported declarations are unrelated to this research.

KWM has received consulting fees from Boehringer Ingelheim, Biogen, Abbvie and honoraria from Boehringer Ingelheim unrelated to the trial; trial support from Boehringer Ingelheim, the NIHR, the Stroke Association, Innovate UK and the British Heart Foundation unrelated to the trial. He was a member of the data monitoring committee for the ARREST trial, unrelated to this research.

AC has received research grants from 10.13039/100004319Pfizer and honoraria from BMS, Pfizer, AstraZeneca and Boeheringer Ingelheim unrelated to this trial.

MK has received honoraria from Astra Zeneca and research funding from the 10.13039/501100000274British Heart Foundation unrelated to this research.

AS holds a patent for the use of xanthine oxidase inhibition for the treatment of angina pectoris.

KD has received conference support from 10.13039/100004336Novartis and honoraria from Allegan unrelated to this research.

DD received payment for image analysis in this study and has received payment for image analysis from MicroTransponder Inc unrelated to this research.

LS is a member of the executive committee of the British and Irish Association of Stroke Physicians. She is a member of stroke specialist advisory committee of the Joint Royal College and Training Board in the UK.

DW has received consulting fees and honoraria from Bayer, Alnylam, Portola and NovoNordisk unrelated to this research. He is chair of the IDMC for the OXHARP trial. He is president-elect of British and Irish Association of Stroke Physicians. He is Chair of Association of British Neurologists Stroke Advisory Group. He serves on the Editorial Board of Practical Neurology, European Journal of Neurology and International Journal of Stroke. He is Chair of UK Stroke Forum. He is member of NICE AI in Stroke Diagnosis Guideline Committee. He is Chief Investigator for the OPTIMAS and Prohibit-ICH trials. He serves on the steering committee and co-investigator for LACI-2, TICH-3, RECAST-3. He serves on the steering committee and is co-investigator for RESTART, TICH-2.

The other authors declare they have no competing interests.
