# Supplementary material for: Xanthine oxidase inhibition and white matter hyperintensity progression following ischaemic stroke and transient ischaemic attack (XILO-FIST): a multicentre, double-blinded, randomised, placebo-controlled trial
Source: eClinicalMedicine. 2023 Feb 16;57:101863. doi: 10.1016/j.eclinm.2023.101863 (PMC9972492; doi:10.1016/j.eclinm.2023.101863)
Supplement: Supplementary material [file mmc1.docx]

**XILO-FIST supplementary data**

**Table of contents**

| **Trial Steering Committee and Independent Data Monitoring Committee members** | **Page 2** |
| --- | --- |
| **Appendix table 1. Detailed list of inclusion and exclusion criteria** | **Page 2** |
| **Appendix table 2. Summary of study procedures** | **Page 3** |
| **Appendix table 3. Summary of imaging sequences** | **Page 4** |
| **Appendix table 4. Summary of substantial protocol amendments** | **Page 4** |
| **Appendix table 5. Enrolment by site** | **Page 5** |
| **Appendix table 6A and 6B. Results of per protocol analysis for primary and secondary outcomes** | **Page 6** |
| **Appendix table 7. Results of subgroup analyses** | **Page 7** |
| **Appendix section 8. Trial protocol** |  |

**Trial Steering Committee members**

Prof Philip Bath (Chair)

Ms Shannon Amolis

Prof Jesse Dawson

Dr Kirsten Forbes

Dr Richard Francis

Prof Mark Kearney

Prof Kennedy Lees

Prof Alex McConnachie

Dr Marc Randall

Mr Charles Ross (Lay Member)

Prof Allan Struthers

**Independent Data Monitoring Committee members**

Prof Peter Sandercock (Chair)

Prof Gary Ford

Dr Chris Sutton

Dr William Whiteley

**Appendix table 1. Detailed list of inclusion and exclusion criteria**

| **Inclusion Criteria** | **Exclusion Criteria** |
| --- | --- |
| Ischaemic Stroke/ Ischaemic lesion on brain imaging in relevant anatomical territory in patients with transient ischaemic attack | Modified Rankin scale score of 5 |
| Age greater then 50 | Diagnosis of Dementia |
| Consent within one month of Stroke | Cognitive impairment deemed sufficient to compromise capacity or comply with the protocol |
|  | Dependent on daily help from others for basic activities prior to stroke |
|  | Significant co-morbidity or frailty likely to cause death within 24 months |
|  | Contra-Indication to or indication for administration of allopurinol |
|  | Concurrent azathioprine, 6-mercaptopurine therapy, other cytotoxic therapies, cyclosporin, theophylline and didanosine |
|  | Significant hepatic impairment |
|  | Estimated Glomerular Filtration Rate (eGFR) < 30 mls/min |
|  | Contraindication to MRI scanning |
|  | Women of childbearing potential |
|  | Prisoners |
|  | Active participation in another CTIMP or device trial or participation within the past month |
|  | eGFR < 60 and of Korean, Han Chinese or Thai descent |

**Appendix table 2. Summary of study procedures**

| **Activity** | **Run-In Phase** | | **Treatment Phase** | | | | | | |
| --- | --- | --- | --- | --- | --- | --- | --- | --- | --- |
|  | Day 0 | Week 4 | Week 0 | Week 4 | Week 13 | Week 26 | Week 52 | Week 78 | Week 104 |
| Review Eligibility | ✓ | ✓ |  |  |  |  |  |  |  |
| Informed Consent | ✓ |  |  |  |  |  |  |  |  |
| Optimise Preventative Therapy | ✓ |  |  |  |  |  |  |  |  |
| Clinical Evaluation * | ✓ | ✓ |  | ✓ | ✓ | ✓ | ✓ |  | ✓ |
| Safety Blood Tests # | ✓ | ✓ |  | ✓ | ✓ | ✓ | ✓ |  | ✓ |
| Blood for Uric Acid Level |  | ✓ |  |  |  |  |  |  | ✓ |
| Blood / Urine for Biobanking |  | ✓ |  |  |  |  |  |  | ✓ |
| ECG |  | ✓ |  |  |  |  |  |  | ✓ |
| Echocardiography |  | ✓ |  |  |  |  |  |  |  |
| Determine Cardiac Sub-study Eligibility |  | ✓ |  |  |  |  |  |  |  |
| MRI Brain (± carotid MRI) |  | ✓ |  |  |  |  |  |  | ✓ |
| ABPM |  | ✓ |  | ✓ |  |  |  |  | ✓ |
| Cardiac MRI ‡, n=100 |  | ✓ |  |  |  |  |  |  | ✓ |
| Detailed Cognitive Function Evaluation |  | ✓ |  |  |  |  |  |  | ✓ |
| Assessment of run-in completion |  | ✓ |  |  |  |  |  |  |  |
| Randomisation |  |  | ✓ |  |  |  |  |  |  |
| Dispense |  |  | ✓ | ✓ | ✓ | ✓ | ✓ | ✓ |  |
| Return / Count IMP |  |  |  | ✓ | ✓ | ✓ | ✓ | ✓ | ✓ |
| Adverse Event Review |  |  | ✓ | ✓ | ✓ | ✓ | ✓ | ✓ | ✓ |
| *includes measures of stroke severity at week 4, modified Rankin scale score and MoCA at week 52 and blood pressure at all visits except week 78 and weight at week 104). # includes FBC, U+E, LFTs. ‡ sub-study eligible participants only. IMP = investigational medicinal product. ABPM = ambulatory blood pressure monitoring. Note that the run in week 4 and treatment phase week 0 visits can take place concurrently. Participants will also be contacted by telephone at week 105, which marks the end of the study. | | | | | | | | | |

**Appendix table 3. Summary of imaging sequences**

| Scan | Sequence | Orientation | Time to echo | Repetition time | Inversion Time | Slice Thickness | Slice Gap | Matrix | Field of View | N Slices |
| --- | --- | --- | --- | --- | --- | --- | --- | --- | --- | --- |
| T1 weighted | TFL | SAG | 1.85 | 2000 | 900 | 1.0 | 50% | 256 * 100 | 255 | 176 |
| T2 weighted | SPC | TRA | 404 | 3000 | -- | 0.9 | -- | 256 * 100 | 230 | 176 |
| FLAIR | SPCIR | SAG | 397 | 5000 | 1800 | 1.0 | -- | 256 * 100 | 255 | 160 |
| DWI | RESOLVE | TRA | 62 | 4100 | -- | 4 | 30% | 224 * 100 | 220 | 27 |
| SWI | SWI_r | TRA | 20 | 24 | -- | 1.5 | 20% | 256 * 95 | 230 | 96 |
| Imaging sequences are those used at the Queen Elizabeth University and Glasgow Royal Infirmary sites. Other sites harmonised sequences according to these parameters. FLAIR = fluid attenuated inversion recovery. DWI = diffusion weighted imaging. SWI = susceptibility weighted imaging. | | | | | | | | | | |

**Appendix table 4. Summary of substantial amendments and protocol changes**

| **Protocol Version** | **Date** | **Summary of Changes** |
| --- | --- | --- |
| 1.0 | 24 Apr 2014 | N/A |
| 2.0 | 10 Jun 2014 | Clarifications of Section 6.12: Sample Size; Section 8.1.5: Record Retention; Section 8.16: Archiving; Format changes |
| 3.0 | 02 Dec 2014 | Minor revisions to Section 1.5: Summary of Risk Assessment; Section 3: Study Design; Section 4: Investigational Drug Information and Procurement; Section 5: Pharmacovigilance  Updates to Section 5.2 Recording and Reporting AEs/SAEs; Section 5.3: Unblinding for SUSAR Reporting; Section 5.4: Annual Safety Reports; Section 6.3: Cardiac Sub-Study Primary Endpoint  Renaming of Section 6.4: Sub-Analysis Primary Endpoint  Inclusion of Appendix 3: Expected Events and Appendix 4: Predefined Events of Special Interest |
| 3.1 | 08 Jan 2015 | Minor revision to Section 4.9: Concomitant Medicines |
| 3.2 | 21 Apr 2015 | Clarification of Section 5.2.2: Reporting  Revision of Appendix 1: Sample Collection for Biobanking |
| 3.3 | 24 Jul 2015 | Update to Section 3.4.1: Cardiac MRI Sub-Study |
| 4.0 | 17 Sep 2015 | Update to Section 3.3: Main Trial Exclusion Criteria  Inclusion of Summary of Changes |
| 4.1 | 02 Nov 2015 | Clarification regarding size of image adjudication panel (2 members with 3^rd^ reviewer available for consensus if needed). |
| 4.2 | 15 Jul 2016 | Addition of questionnaires |
| 4.3 | 06 Dec 2016 | Clarification of sample size required for carotid sub study.  Change to cardiac sub study eligibility |
| 4.4 | 23 Apr 2018 | Excluding all patients of Korean, Han Chinese or Thai descent unless negative HLA-B*5801 status is known |
| 4.5 | 03 Dec 2018 | Clarification of Image reviewers  Termination of cardiac sub study  Administrative changes  Reference to separate 7T MRI sub-study protocol |
| 4.6 | 31 July 2019 | Removal of secondary objective ‘to establish whether allopurinol reduces LVH after ischaemic stroke’ |
| 5.0 | 19 March 2020 | Change to time window for week 104 visit in response to COVID-19 pandemic  Change to allow week 78 visit to be conducted by telephone in response to COVID-19 pandemic |

**Appendix table 5. Enrolment by site**

| **Site name** | PI |
| --- | --- |
| **Enrolment of <15 participants** | |
| Western Infirmary, Glasgow, UK | Jesse Dawson |
| Wansbeck General Hospital, Wansbeck, UK | Christopher Price |
| Royal Victoria Hospital, Newcastle, UK | Alexander Dyker |
| North Tyneside General Hospital, North Shields, UK | Christopher Price |
| St Thomas’ Hospital, London, UK | Ajay Bhalla |
| Leeds General Infirmary, UK | Hassan Ahamad |
| Darent Valley Hospital, Dartford, UK | Imran Ashraf |
| Sunderland Royal Hospital, Sunderland, UK | Naweed Sattar |
| Broomfield Hospital, Chelmsford, UK | Ramanathan Kirthivasan |
| Royal United Hospital, Bath, UK | Louise Shaw |
| Southend University Hospital, Southend, UK | Paul Guyler |
| University College London, London, UK | David Werring |
| Luton and Dunstable Hospital, Luton, UK | Lakshmanan Sekaran |
| Altnagelvin Hospital, Londonderry, UK | Elizabeth Best |
| South West Acute Hospital, Enniskillen, UK | Breffni Keegan |
| Royal London Hospital, London, UK | Sageet Amlani |
| **Enrolment of 15-30 participants** | |
| Ninewells Hospital, Dundee, UK | Alex Doney |
| Aberdeen Royal Infirmary, Aberdeen, UK | Mary-Joan Macleod |
| Nottingham University Hospital, Nottingham, UK | Ganesh Subramanian |
| **Enrolment of > 30 participants** | |
| Queen Elizabeth University Hospital, Glasgow, UK | Jesse Dawson |
| Glasgow Royal Infirmary, Glasgow, UK | Christine McAlpine |
| University Hospital Monklands, Airdrie, UK | Mark Barber |

**Appendix table 6A and 6B. Results of per protocol analysis for primary and secondary outcomes**

**Table 6a**

| **Outcome** | **Between group difference** | **95% confidence interval** | **P value** |
| --- | --- | --- | --- |
| RPS | -0.28 | -0.65 to 0.09 | 0.14 |
| WMH volume (log) | 0.00 | -0.06 to 0.06 | 1.00 |
| Schmidt progression score | OR 1.10 | 0.69 to 1.73 | 0.69 |
| Scheltens score | -0.76 | -1.43 to -0.08 | 0.03 |
| Fazekas score | -0.07 | -0.21 to 0.06 | 0.29 |
| New infarction | OR 1.40 | 0.72 to 2.71 | 0.31 |

**Table 6B**

| **Outcome** | **Between group difference** | **95% confidence interval** | **P value** |
| --- | --- | --- | --- |
| ABPM SBP change at week 4 | -3.23 | -5.63 to -0.82 | 0.009 |
| ABPM SBP change at week 104 | -2.38 | -5.74 to 0.99 | 0.17 |
| ABPM DBP change at week 4 | -1.25 | -2.72 to 0.21 | 0.09 |
| ABPM DBP change at week 104 | -1.20 | -3.19 to 0.79 | 0.24 |
| All blood pressure values are in mmHg. SBP = systolic blood pressure. DBP = diastolic blood pressure. | | | |

**Appendix table 7. Results of subgroup analyses**

**WHETHER PARTICIPATION WAS COMPLETED BEFORE THE INTRODUCTION OF COVID RESTRICTIONS**

**Completed study prior to 16^th^ March 2020**

*Intention to treat analyses*

| **Outcome** | **Between group difference** | **95% CI** | **P value** |
| --- | --- | --- | --- |
| RPS | -0.15 | -0.55 to 0.25 | 0.46 |
| WMH volume (log) | -0.003 | -0.10 to 0.10 | 0.96 |

| **Outcome** | **Between group difference** | **95% CI** | **P value** |
| --- | --- | --- | --- |
| ABPM SBP week 4 | -3.28 | -5.67 to -0.89 | 0.007 |
| ABPM SBP week 104 | -2.84 | -5.88 to 0.20 | 0.07 |
| ABPM DBP week 4 | -1.08 | -2.49 to 0.33 | 0.13 |
| ABPM DBP week 104 | -0.85 | -2.67 to 0.96 | 0.36 |
| All blood pressure values are in mmHg. SBP = systolic blood pressure. DBP = diastolic blood pressure. | | | |

**Completed study on or after 16^th^ March 2020**

*Intention to treat analyses*

| **Outcome** | **Between group difference** | **95% CI** | **P value** |
| --- | --- | --- | --- |
| RPS | -0.20 | -0.92 to 0.52 | 0.58 |
| WMH volume (log) | 0.17 | -0.03 to 0.34 | 0.09 |

| **Outcome** | **Between group difference** | **95% CI** | **P value** |
| --- | --- | --- | --- |
| ABPM SBP week 4 | -4.76 | -11.74 to 2.22 | 0.18 |
| ABPM SBP week 104 | n/a | n/a | n/a |
| ABPM DBP week 4 | -2.15 | -5.80 to 1.50 | 0.24 |
| ABPM DBP week 104 | n/a | n/a | n/a |
| All blood pressure values are in mmHg. SBP = systolic blood pressure. DBP = diastolic blood pressure. | | | |

**AGE DEFINED BY THE MEDIAN**

**Below median age (65 years)**

*Intention to treat analyses*

| **Outcome** | **Between group difference** | **95% CI** | **P value** |
| --- | --- | --- | --- |
| RPS | -0.21 | -0.65 to 0.23 | 0.34 |
| WMH volume (log) | 0.0 | -0.14 to 0.14 | 1.00 |

| **Outcome** | **Between group difference** | **95% CI** | **P value** |
| --- | --- | --- | --- |
| ABPM SBP week 4 | -1.85 | -5.06 to 1.36 | 0.26 |
| ABPM SBP week 104 | -4.08 | -8.55 to 0.39 | 0.07 |
| ABPM DBP week 4 | -0.33 | -2.07 to 1.41 | 0.71 |
| ABPM DBP week 104 | -1.55 | -3.97 to 0.88 | 0.21 |
| All blood pressure values are in mmHg. SBP = systolic blood pressure. DBP = diastolic blood pressure. | | | |

**Above median age (65 years)**

| **Outcome** | **Between group difference** | **95% CI** | **P value** |
| --- | --- | --- | --- |
| RPS | -0.12 | -0.67 to 0.44 | 0.68 |
| WMH volume (log) | 0.04 | -0.08 to 0.15 | 0.50 |

| **Outcome** | **Between group difference** | **95% CI** | **P value** |
| --- | --- | --- | --- |
| ABPM SBP week 4 | -4.43 | -7.51 to -1.34 | 0.005 |
| ABPM SBP week 104 | -0.13 | -4.79 to 4.53 | 0.96 |
| ABPM DBP week 4 | -1.83 | -3.80 to 0.15 | 0.07 |
| ABPM DBP week 104 | 0.91 | -2.12 to 3.93 | 0.55 |
| All blood pressure values are in mmHg. SBP = systolic blood pressure. DBP = diastolic blood pressure. | | | |

**BASELINE URIC ACID LEVEL DEFINED BY THE MEDIAN (330 μmol/l)**

**Baseline uric acid below median level**

| **Outcome** | **Between group difference** | **95% CI** | **P value** |
| --- | --- | --- | --- |
| RPS | 0.16 | -0.44 to 0.76 | 0.60 |
| WMH volume (log) | 0.03 | -0.11 to 0.16 | 0.67 |

| **Outcome** | **Between group difference** | **95% CI** | **P value** |
| --- | --- | --- | --- |
| ABPM SBP week 4 | -1.99 | -5.90 to 1.92 | 0.32 |
| ABPM SBP week 104 | -3.95 | -9.19 to 1.30 | 0.14 |
| ABPM DBP week 4 | -0.53 | -2.52 to 1.45 | 0.60 |
| ABPM DBP week 104 | -0.12 | -2.75 to 2.51 | 0.93 |
| All blood pressure values are in mmHg. SBP = systolic blood pressure. DBP = diastolic blood pressure. | | | |

**Baseline uric acid above median level**

| **Outcome** | **Between group difference** | **95% CI** | **P value** |
| --- | --- | --- | --- |
| RPS | 0.02 | -0.57 to 0.60 | 0.96 |
| WMH volume (log) | -0.03 | -0.20 to 0.14 | 0.71 |

| **Outcome** | **Between group difference** | **95% CI** | **P value** |
| --- | --- | --- | --- |
| ABPM SBP week 4 | -4.91 | -8.79 to -1.02 | 0.01 |
| ABPM SBP week 104 | 0.61 | -5.01 to 6.23 | 0.83 |
| ABPM DBP week 4 | -1.11 | -3.70 to 1.47 | 0.40 |
| ABPM DBP week 104 | -0.32 | -3.93 to 3.29 | 0.86 |
| All blood pressure values are in mmHg. SBP = systolic blood pressure. DBP = diastolic blood pressure. | | | |

| 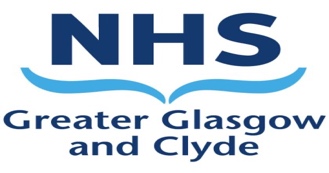 | **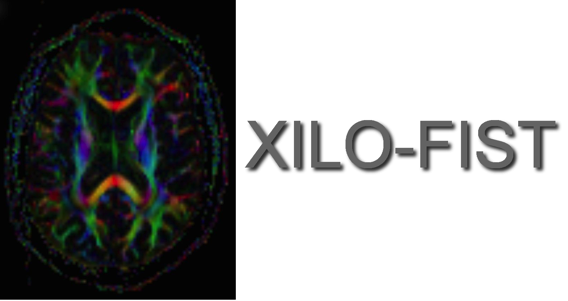** | | 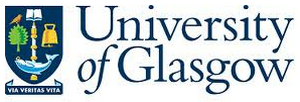 |
| --- | --- | --- | --- |
|  | | | |
|  | | | |
| **Xanthine Oxidase Inhibition for Improvement of Long-Term Outcomes Following Ischaemic Stroke and Transient Ischaemic Attack** | | | |
|  | | | |
|  | | | |
|  | |  | |
| *Running Title* | | XILO-FIST | |
|  | |  | |
|  | |  | |
| *Lay Title* | | Allopurinol, brain lesions, heart size and blood pressure after ischaemic stroke | |
|  | |  | |
|  | |  | |
| *Protocol Version* | | 5.0 | |
|  | |  | |
|  | |  | |
| *Protocol Date* | | 19^th^ March 2020 | |
|  | |  | |
|  | |  | |
| *EudraCT Number* | | 2013-004235-77 | |
|  | |  | |
|  | |  | |
| *REC Reference Number* | | 14/WS/0113 | |
|  | |  | |
|  | |  | |
| *Sponsor Protocol Number* | | GN12MT494 | |
|  | |  | |
|  | |  | |
| *Sponsor* | | NHS Greater Glasgow and Clyde  University of Glasgow | |
|  | |  | |
|  | |  | |
| *Funder* | | Joint Stroke Association and British Heart Foundation Programme Grant (TSA BHF 2013/01) | |
|  | |  | |
|  | | | |
|  | | | |
| This study will be performed according to the Research Governance Framework for Health and Community Care (Second edition, 2006) and The Medicines for Human Use(Clinical Trials) Regulations, 2004 SI 2004:1031 (as amended) and WORLD MEDICAL ASSOCIATION DECLARATION OF HELSINKI Ethical Principles for Medical Research Involving Human Subjects 1964 (as amended). | | | |
|  | | | |
|  | | | |
|  | | | |
| **CONFIDENTIAL** | | | |
|  | | | |

|  | | |
| --- | --- | --- |
| **SUMMARY OF CHANGES** | | |
|  | | |
| **Protocol Version** | **Date** | **Summary of Changes** |
| 1.0 | 24 Apr 2014 | N/A |
| 2.0 | 10 Jun 2014 | Clarifications of Section 6.12: Sample Size; Section 8.1.5: Record Retention; Section 8.16: Archiving; Format changes |
| 3.0 | 02 Dec 2014 | Minor revisions to Section 1.5: Summary of Risk Assessment; Section 3: Study Design; Section 4: Investigational Drug Information and Procurement; Section 5: Pharmacovigilance  Updates to Section 5.2 Recording and Reporting AEs/SAEs; Section 5.3: Unblinding for SUSAR Reporting; Section 5.4: Annual Safety Reports; Section 6.3: Cardiac Sub-Study Primary Endpoint  Renaming of Section 6.4: Sub-Analysis Primary Endpoint  Inclusion of Appendix 3: Expected Events and Appendix 4: Predefined Events of Special Interest |
| 3.1 | 08 Jan 2015 | Minor revision to Section 4.9: Concomitant Medicines |
| 3.2 | 21 Apr 2015 | Clarification of Section 5.2.2: Reporting  Revision of Appendix 1: Sample Collection for Biobanking |
| 3.3 | 24 Jul 2015 | Update to Section 3.4.1: Cardiac MRI Sub-Study |
| 4.0 | 17 Sep 2015 | Update to Section 3.3: Main Trial Exclusion Criteria  Inclusion of Summary of Changes |
| 4.1 | 02 Nov 2015 | Clarification regarding size of image adjudication panel (2 members with 3^rd^ reviewer available for consensus if needed). |
| 4.2 | 15 Jul 2016 | Addition of questionnaires |
| 4.3 | 06 Dec 2016 | Clarification of sample size required for carotid sub study.  Change to cardiac sub study eligibility |
| 4.4 | 23 Apr 2018 | Excluding all patients of Korean, Han Chinese or Thai descent unless negative HLA-B*5801 status is known |
| 4.5 | 03 Dec 2018 | Clarification of Image reviewers  Termination of cardiac sub study  Administrative changes  Reference to separate 7T MRI sub-study protocol |
| 4.6 | 31 July 2019 | Removal of secondary objective ‘to establish whether allopurinol reduces LVH after ischaemic stroke’ |
| 5.0 | 19 March 2020 | Change to time window for week 104 visit in response to COVID-19 pandemic  Change to allow week 78 visit to be conducted by telephone in response to COVID-19 pandemic |

|  | |
| --- | --- |
| **CHIEF INVESTIGATOR** | |
|  | |
|  | |
| **Prof Jesse DAWSON** | |
|  | |
| Professor of Stroke Medicine | |
|  | |
| College of Medicine, Veterinary & Life Sciences | |
|  | |
| Institute of Cardiovascular and Medical Sciences | |
|  | |
| Queen Elizabeth University Hospital | |
|  | |
| 1345 Govan Road | |
|  | |
| Glasgow | |
|  | |
| G51 4TF | |
|  | |
| T: 0141 451 5868 | |
|  | |
| E: [jesse.dawson@glasgow.ac.uk](mailto:jesse.dawson@glasgow.ac.uk) | |
|  | |
|  | |
|  | |
| **CO-GRANT HOLDERS** | |
|  | |
|  |  |
| **Dr Niall BROOMFIELD** | **Dr Krishna DANI** |
|  |  |
| Honorary Clinical Teacher | Clinical Lecturer (Stroke and Brain Imaging) |
|  |  |
| Psychological Medicine | Institute of Neuroscience and Psychology |
|  |  |
| Primary Services HQ | R24, Level 1 |
|  |  |
| Gartnavel Royal Hospital | Neurology Block |
|  |  |
| Glasgow | Queen Elizabeth University Hospital |
|  |  |
| G12 0XH | Glasgow |
|  |  |
| T: 0141 211 2134 | G51 4TF |
|  |  |
| E: [niall.broomfield@glasgow.ac.uk](mailto:niall.broomfield@glasgow.ac.uk) | T: 0141 211 7016 |
|  |  |
|  | E: [krishna.dani@glasgow.ac.uk](mailto:krishna.dani@glasgow.ac.uk) |
|  |  |
|  |  |
|  |  |
| **Dr Alex DONEY** | **Dr Kirsten FORBES** |
|  |  |
| Consultant Physician & Hon. Senior Lecturer | Honorary Clinical Senior Lecturer |
|  |  |
| Ninewells Hospital and Medical School | Department of Neurological Sciences |
|  |  |
| Dundee | Queen Elizabeth University Hospital |
|  |  |
| DD1 9SY | 1345 Govan Road |
|  |  |
| T: 01382 740 425 | Glasgow |
|  |  |
| E: [alex.doney@nhs.net](mailto:alex.doney@nhs.net) | G51 4TF |
|  |  |
|  | T: 0141 201 2141 |
|  |  |
|  | E: [kirsten.forbes@glasgow.ac.uk](mailto:kirsten.forbes@glasgow.ac.uk) |
|  |  |
|  |  |
|  | |

|  | |
| --- | --- |
| **CO-GRANT HOLDERS (cont.)** | |
|  | |
|  |  |
| **Professor Graeme HOUSTON** | **Professor Kennedy LEES** |
|  |  |
| Medical Research Institute | Professor of Cerebrovascular Medicine |
|  |  |
| Cardiovascular and Lung Biology | College of Medicine, Veterinary and Life Sciences |
|  |  |
| Level 6 | Institute of Cardiovascular and Medical Sciences |
|  |  |
| Ninewells Hospital and Medical School | Queen Elizabeth University Hospital |
|  |  |
| Dundee | 1345 Govan Road |
|  |  |
| DD1 9SY | Glasgow |
|  |  |
| T: 01382 632 651 | G51 4TF |
|  |  |
| E: [ghouston@nhs.net](mailto:ghouston@nhs.net) | T: 0141 330 4448 |
|  |  |
|  | E: [kennedy.lees@glasgow.ac.uk](mailto:kennedy.lees@glasgow.ac.uk) |
|  |  |
|  |  |
|  |  |
| **Dr John McLEAN** | **Professor Keith MUIR** |
|  |  |
| Institute of Neuroscience and Psychology | SINAPSE Chair of Clinical Imaging (Stroke and Brain Imaging) |
|  |  |
| Neurology Block | Institute of Neuroscience and Psychology |
|  |  |
| Queen Elizabeth University Hospital | R139, Level 1 |
|  |  |
| Glasgow | Neurology Block |
|  |  |
| G51 4TF | Queen Elizabeth University Hospital |
|  |  |
| E: [john.mclean@nhs.net](mailto:john.mclean@nhs.net) | Glasgow |
|  |  |
|  | G51 4TF |
|  |  |
|  | T: 0141 201 2502 |
|  |  |
|  | E: [keith.muir@glasgow.ac.uk](mailto:keith.muir@glasgow.ac.uk) |
|  |  |
|  |  |
|  |  |
| **Dr Terry QUINN** | **Professor Allan STRUTHERS** |
|  |  |
| Clinical Senior Lecturer | University of Dundee |
|  |  |
| Academic Geriatric Medicine | Department of Clinical Pharmacology and Therapeutics |
|  |  |
| Fourth Floor, Walton Building | Ninewells Hospital and Medical School |
|  |  |
| Glasgow Royal Infirmary | Dundee |
|  |  |
| G4 0SF | DD1 9SY |
|  |  |
| T: 0141 211 4976 | T: 01382 633 178 |
|  |  |
| E: [terry.quinn@glasgow.ac.uk](mailto:terry.quinn@glasgow.ac.uk) | E: [a.d.struthers@dundee.ac.uk](mailto:a.d.struthers@dundee.ac.uk) |
|  |  |
|  |  |
|  |  |

|  | |
| --- | --- |
| **CO-GRANT HOLDERS (cont.)** | |
|  | |
|  |  |
| **Professor Matthew WALTERS** |  |
|  |  |
| Professor in Clinical Pharmacology |  |
|  |  |
| College of Medicine, Veterinary and Life Sciences |  |
|  |  |
| Institute of Cardiovascular and Medical Sciences |  |
|  |  |
| Queen Elizabeth University Hospital |  |
|  |  |
| 1345 Govan Road |  |
|  |  |
| Glasgow |  |
|  |  |
| G51 4TF |  |
|  |  |
| T: 0141 330 5691 |  |
|  |  |
| E: [matthew.walters@glasgow.ac.uk](mailto:matthew.walters@glasgow.ac.uk) |  |
|  |  |
|  | |
|  | |
| **TRIAL STATISTICIAN** | |
|  | |
|  |  |
| **Dr Alex McCONNACHIE** |  |
|  |  |
| Assistant Director of Biostatistics |  |
|  |  |
| Robertson Centre for Biostatistics |  |
|  |  |
| University of Glasgow |  |
|  |  |
| Boyd Orr Building |  |
|  |  |
| University Avenue |  |
|  |  |
| G12 8QQ |  |
|  |  |
| T: 0141 330 4744 |  |
|  |  |
| E: [alex.mcconachie@glasgow.ac.uk](mailto:alex.mcconachie@glasgow.ac.uk) |  |
|  |  |
|  | |
|  | |
| **DATA CENTRE** | |
|  | |
|  |  |
| **Robertson Centre for Biostatistics** |  |
|  |  |
| University of Glasgow |  |
|  |  |
| Boyd Orr Building |  |
|  |  |
| University Avenue |  |
|  |  |
| G12 8QQ |  |
|  |  |
| T: 0141 330 4744 |  |
|  |  |

|  | |
| --- | --- |
| **Key Collaborators** | |
|  | |
|  |  |
| **Dr David DICKIE** |  |
|  |  |
| Stroke Association Research Fellow |  |
|  |  |
| College of Medicine, Veterinary and Life Sciences |  |
|  |  |
| Institute of Cardiovascular and Medical Sciences |  |
|  |  |
| Queen Elizabeth University Hospital |  |
| 1345 Govan Road |  |
|  |  |
| Glasgow |  |
| G51 4TF |  |
| [David.dickie@glasgow.ac.uk](mailto:David.dickie@glasgow.ac.uk) |  |
|  |  |

|  | |
| --- | --- |
| **CHAIR OF DATA SAFETY MONITORING COMMITTEE** | |
|  | |
|  |  |
| **Professor Peter SANDERCOCK** |  |
|  |  |
| Professor of Medical Neurology |  |
|  |  |
| Bramwell Dott Building |  |
|  |  |
| Department of Clinical Neurosciences |  |
|  |  |
| Western General Hospital |  |
|  |  |
| Edinburgh |  |
|  |  |
| EH4 2XU |  |
|  |  |
| T: 0131 537 2927 |  |
|  |  |
| E: [peter.sandercock@ed.ac.uk](mailto:peter.sandercock@ed.ac.uk) |  |
|  |  |
|  | |
|  | |
| **CHAIR OF TRIAL STEERING COMMITTEE** | |
|  | |
|  |  |
| **Professor Philip BATH** |  |
|  |  |
| Stroke Association Professor of Stroke Medicine |  |
|  |  |
| Room B51 Clinical Sciences Building |  |
|  |  |
| Nottingham City Hospital |  |
|  |  |
| Hucknall Road |  |
|  |  |
| Nottingham |  |
|  |  |
| NG5 1PB |  |
|  |  |
| T: 0115 823 1765 |  |
|  |  |
| E: [philip.bath@nottingham.ac.uk](mailto:philip.bath@nottingham.ac.uk) |  |
|  |  |
|  | |
|  | |
| **PHARMACY** | |
|  | |
|  |  |
| **Dr Elizabeth DOUGLAS** |  |
|  |  |
| Clinical Trials Pharmacist |  |
|  |  |
| NHS Greater Glasgow and Clyde |  |
|  |  |
| Clinical Research & Development |  |
|  |  |
| 1^st^ Floor, ward 11 |  |
|  |  |
| Dykebar Hospital |  |
|  |  |
| Grahamston Road |  |
| Paisley |  |
| PA2 7DE |  |
|  |  |
| T: 0141 314 4073 |  |
|  |  |
| E: [elizabeth.douglas@ggc.scot.nhs.uk](mailto:elizabeth.douglas@ggc.scot.nhs.uk) |  |
|  |  |

|  | |
| --- | --- |
| **SPONSOR** | |
|  | |
|  | |
| This clinical trial is co-sponsored by NHS Greater Glasgow and Clyde* and University of Glasgow  **Sponsor’s Representative** | |
|  | |
| **Contact:** |  |
|  |  |
| **Dr Maureen TRAVERS** |  |
|  |  |
| Academic Research Co-ordinator |  |
|  |  |
| NHS Greater Glasgow and Clyde |  |
|  |  |
| Clinical Research & Development |  |
|  |  |
| 1^st^ Floor, Ward 11 |  |
|  |  |
| Dykebar Hospital |  |
|  |  |
| Grahamston Road |  |
| Paisley |  |
|  |  |
| PA2 7DE |  |
|  |  |
| T: 0141 314 4012 |  |
|  |  |
| E: [maureen.travers@ggc.scot.nhs.uk](mailto:maureen.travers@ggc.scot.nhs.uk) |  |
|  |  |
|  | |
|  | |
| **FUNDING BODY** | |
|  | |
|  | |
| Funded via a Stroke Association / British Heart Foundation Joint Programme Grant (TSA BHF 2013/01). | |
|  | |
| **Administered by:** |  |
|  |  |
| **The Stroke Association** |  |
|  |  |
| Research Department |  |
|  |  |
| Stroke House |  |
|  |  |
| 240 City Road |  |
|  |  |
| London |  |
|  |  |
| EC1V 2PR |  |
|  |  |

|  | |
| --- | --- |
| **X**anthine oxidase **I**nhibition for improvement of **L**ong-term **O**utcomes **F**ollowing **I**schaemic **S**troke and **T**ransient ischaemic attack (**XILO-FIST**) | |
|  | |
|  | |
|  |  |
| *Chief Investigator:* | **Dr Jesse DAWSON** |
|  |  |
|  | Professor of Stroke Medicine |
|  |  |
|  | College of Medicine, Veterinary & Life Sciences |
|  |  |
|  | Institute of Cardiovascular and Medical Sciences |
|  |  |
|  | Queen Elizabeth University Hospital |
|  |  |
|  | Room M0.05 |
|  |  |
|  | Glasgow |
|  |  |
|  | G51 4TF |
|  |  |
| *Signature:* |  |
|  |  |
| *Date:* | 19 March 2020 |
|  |  |
|  |  |
|  |  |
| *Sponsor Representative:* | **Dr Maureen Travers** |
|  |  |
|  | Academic Research Co-ordinator |
|  |  |
|  | NHS Greater Glasgow and Clyde |
|  |  |
|  | Clinical Research & Development |
|  |  |
|  | 2nd Floor |
|  |  |
|  | West Glasgow Ambulatory Care Hospital |
|  |  |
|  | Dalnair Street |
|  |  |
|  | Glasgow |
|  |  |
|  | G3 8SW |
|  |  |
| *Signature:* |  |
|  |  |
| *Date:* |  |
|  |  |

|  |
| --- |
| **TABLE OF CONTENTS** |
|  |
|  |
|  |
| [LIST OF ABBREVATIONS 13](#_Toc418234385)  [STUDY SYNOPSIS 15](#_Toc418234386)  [1 INTRODUCTION 18](#_Toc418234387)  [1.1 BACKGROUND 18](#_Toc418234388)  [1.2 STUDY HYPOTHESIS 19](#_Toc418234389)  [1.3 PILOT DATA TO SUPPORT BENEFICIAL EFFECTS OF ALLOPURINOL / SYSTEMATIC REVIEW AND META-ANALYSIS 19](#_Toc418234390)  [1.4 TOLERABILITY OF ALLOPURINOL IN PATIENTS WITH RECENT STROKE 20](#_Toc418234391)  [1.5 SUMMARY OF RISK ASSESSMENT 20](#_Toc418234392)  [2 TRIAL OBJECTIVES AND PURPOSE OF CLINICAL TRIAL 21](#_Toc418234393)  [3 STUDY DESIGN 22](#_Toc418234394)  [3.1 STUDY POPULATION 22](#_Toc418234395)  [3.2 MAIN TRIAL INCLUSION CRITERIA 22](#_Toc418234396)  [3.3 MAIN TRIAL EXCLUSION CRITERIA 23](#_Toc418234397)  [3.4 ADDITIONAL SUB-STUDY INCLUSION CRITERIA 24](#_Toc418234398)  [3.4.1 CARDIAC MRI SUB-STUDY 24](#_Toc418234399)  [3.4.2 CAROTID MRI SUB-STUDY 24](#_Toc418234400)  [3.5 IDENTIFICATION OF PARTICIPANTS AND CONSENT 24](#_Toc418234401)  [3.6 VISIT SCHEDULE 25](#_Toc418234402)  [3.6.1 RUN-IN PHASE 25](#_Toc418234403)  [3.6.2 TREATMENT PHASE 26](#_Toc418234404)  [3.7 CARDIAC SUB-STUDY VISIT SCHEDULE 27](#_Toc418234405)  [3.8 SUB-ANALYSIS VISIT SCHEDULE 27](#_Toc418234406)  [3.9 BRAIN MRI IMAGING PROTOCOL 28](#_Toc418234407)  [3.10 CAROTID MRI IMAGING PROTOCOLS 28](#_Toc418234408)  [3.11 CARDIAC MRI IMAGING PROTOCOLS 29](#_Toc418234409)  [3.12 AMBULATORY BLOOD PRESSURE MONITORING 29](#_Toc418234410)  [3.13 CLINIC BLOOD PRESSURE MEASUREMENT 29](#_Toc418234411)  [3.14 BRAIN MRI UPLOAD AND DISTRIBUTION AND REVIEW 30](#_Toc418234412)  [3.14.1 MRI IMAGE UPLOAD 30](#_Toc418234413)  [3.14.2 INITIAL QUALITY REVIEW 30](#_Toc418234414)  [3.14.3 BASELINE SCAN REVIEW 30](#_Toc418234415)  [3.14.4 FOLLOW-UP SCAN REVIEW 30](#_Toc418234416)  [3.14.5 SIDE BY SIDE SCAN REVIEW 30](#_Toc418234417)  [3.15 BRAIN MRI RATER TRAINING 31](#_Toc418234418)  [3.16 BRAIN MRI IMAGE ANALYSIS PROTOCOLS 31](#_Toc418234419)  [3.17 CAROTID MRI UPLOAD AND DISTRIBUTION 31](#_Toc418234420)  [3.18 CAROTID MRI IMAGE ANALYSIS 31](#_Toc418234421)  [3.19 CARDIAC MRI UPLOAD AND DISTRIBUTION 31](#_Toc418234422)  [3.20 CARDIAC MRI IMAGE ANALYSIS 32](#_Toc418234423)  [3.21 ABPM INTERPRETATION 32](#_Toc418234424)  [3.22 SAFETY BLOOD TESTING / VENEPUNCTURE 33](#_Toc418234425)  [3.23 BIOBANKING 33](#_Toc418234426)  [3.24 NIHSS AND MRS CERTIFICATION PROCEDURE 34](#_Toc418234427)  [3.25 COGNITIVE TESTING 36](#_Toc418234428)  [3.26 QUALITY OF LIFE ASSESSMENT 36](#_Toc418234429)  [4 INVESTIGATIONAL DRUG INFORMATION AND PROCUREMENT 37](#_Toc418234430)  [4.1 ALLOPURINOL (300 mg TWICE DAILY) 37](#_Toc418234431)  [4.1.1 RATIONALE FOR CHOSEN DOSE 37](#_Toc418234432)  [4.2 STUDY INTERVENTION 37](#_Toc418234433)  [4.3 PATIENT ADVICE 38](#_Toc418234434)  [4.4 FORMULATION AND SOURCE OF DRUG 38](#_Toc418234435)  [4.5 STORAGE AND STABILITY 38](#_Toc418234436)  [4.6 DRUG PROCUREMENT 38](#_Toc418234437)  [4.6.1 DRUG ORDERING 38](#_Toc418234438)  [4.6.2 DRUG ACCOUNTABILITY 39](#_Toc418234439)  [4.7 DESTRUCTION OF UNUSED DRUG 39](#_Toc418234440)  [4.8 EMERGENCY UNBLINDING OF TREATMENT ALLOCATION 39](#_Toc418234441)  [4.9 CONCOMITANT MEDICINES 40](#_Toc418234442)  [4.10 DISPENSING SCHEDULE 40](#_Toc418234443)  [4.11 PROCEDURES FOR SAFETY MONITORING DURING THE TRIAL 40](#_Toc418234444)  [4.12 CRITERIA FOR WITHDRAWAL OF PARTICIPANTS ON SAFETY GROUNDS AND WITHDRAWAL PROCESS 40](#_Toc418234445)  [4.13 PROCEDURE FOR RESTARTING TREATMENT FOLLOWING TREATMENT INTERRUPTION 41](#_Toc418234446)  [4.14 MAINTENANCE OF TRIAL TREATMENT RANDOMISATION CODES AND PROCEDURES FOR UNBLINING 41](#_Toc418234447)  [5 PHARMACOVIGILANCE 42](#_Toc418234448)  [5.1 DEFINITIONS OF ADVERSE EVENTS 42](#_Toc418234449)  [5.2 RECORDING AND REPORTING AEs/SAEs 43](#_Toc418234450)  [5.2.1 RECORDING 43](#_Toc418234451)  [5.2.2 REPORTING 45](#_Toc418234452)  [5.3 UNBLINDING FOR SUSAR REPORTING 45](#_Toc418234453)  [5.4 ANNUAL SAFETY REPORTS 45](#_Toc418234454)  [6 STATISTICS AND DATA ANALYSIS 47](#_Toc418234455)  [6.1 PRIMARY ENDPOINT 47](#_Toc418234456)  [6.2 SECONDARY ENDPOINT(S) 47](#_Toc418234457)  [6.3 CARDIAC SUB-STUDY PRIMARY ENDPOINT 47](#_Toc418234458)  [6.3.1 CARDIAC SUB-STUDY SECONDARY ENDPOINT(S) 47](#_Toc418234459)  [6.4 SUB-ANALYSIS PRIMARY ENDPOINT 48](#_Toc418234460)  [6.4.1 SUB-ANALYSIS SECONDARY ENDPOINT(S) 48](#_Toc418234461)  [6.5 TIME POINTS FOR ENDPOINT EVALUATION 48](#_Toc418234462)  [6.6 STATISTICAL ANALYSIS PLAN 48](#_Toc418234463)  [6.7 GENERAL CONSIDERATIONS 48](#_Toc418234464)  [6.8 EFFICACY ANALYSES 49](#_Toc418234465)  [6.8.1 PRIMARY ENDPOINT 49](#_Toc418234466)  [6.8.2 SECONDARY ENDPOINT 49](#_Toc418234467)  [6.9 PLANNED SUB-GROUP ANALYSIS 49](#_Toc418234468)  [6.10 SAFETY ANALYSES 49](#_Toc418234469)  [6.11 SOFTWARE FOR STATISTICAL ANALYSIS 50](#_Toc418234470)  [6.12 SAMPLE SIZE 50](#_Toc418234471)  [6.13 LEVEL OF SIGNIFICANCE TO BE USED 51](#_Toc418234472)  [6.14 CRITERIA FOR TERMINATION OF TRIAL 51](#_Toc418234473)  [6.15 PROCEDURES FOR ACCOUNTING FOR MISSING DATA 51](#_Toc418234474)  [6.16 PROCEDURES FOR REPORTING AND DEVIATIONS FROM THE ORIGINAL STATISTICAL PLAN 51](#_Toc418234475)  [6.17 SELECTION OF PARTICIPANTS TO BE INCLUDED IN THE ANALYSES 52](#_Toc418234476)  [7 TRIAL CLOSURE / DEFINITION OF END OF TRIAL 53](#_Toc418234477)  [8 SOURCE DATA / DOCUMENTS 54](#_Toc418234478)  [8.1 DATA HANDLING AND RECORD KEEPING 55](#_Toc418234479)  [8.1.1 COMPLETION OF ECRF 55](#_Toc418234480)  [8.1.2 DATA VALIDATION 55](#_Toc418234481)  [8.1.3 DATA SECURITY 55](#_Toc418234482)  [8.1.4 DATABASE SOFTWARE 55](#_Toc418234483)  [8.1.5 RECORD RETENTION 55](#_Toc418234484)  [8.1.6 ARCHIVING 55](#_Toc418234485)  [9 TRIAL MANAGEMENT 56](#_Toc418234486)  [9.1 ROUTINE MANAGEMENT OF TRIAL 56](#_Toc418234487)  [9.2 TRIAL STEERING COMMITTEE (TSC) 56](#_Toc418234488)  [9.3 INDEPENDENT DATA MONITORING COMMITTEE (IDMC) 56](#_Toc418234489)  [9.4 TRIAL WRITING COMMITTEE 56](#_Toc418234490)  [10 STUDY MONITORING 57](#_Toc418234491)  [11 PROTOCOL AMENDMENTS 58](#_Toc418234492)  [12 ETHICAL CONSIDERATIONS 59](#_Toc418234493)  [12.1 ETHICAL CONDUCT OF STUDY 59](#_Toc418234494)  [12.2 INFORMED CONSENT 59](#_Toc418234495)  [13 INSURANCE AND INDEMNITY 60](#_Toc418234496)  [14 FUNDING 61](#_Toc418234497)  [15 CO-SPONSOR RESPONSIBILITIES (NHS GREATER GLASGOW AND CLYDE / UNIVERSITY OF GLASGOW) 62](#_Toc418234498)  [16 ANNUAL REPORTS 63](#_Toc418234499)  [17 DISSEMINATION OF FINDINGS 64](#_Toc418234500)  [18 REFERENCES 65](#_Toc418234501)  [APPENDIX 1. 68](#_Toc418234502)  [APPENDIX 2. 69](#_Toc418234503)  [APPENDIX 3. 72](#_Toc418234504) |
|  |

|  | |
| --- | --- |
| **LIST OF ABBREVATIONS** | |
|  | |
|  | |
|  |  |
| 1.5 or 3T | 1.5 or 3 Tesla |
|  |  |
|  |  |
| ABPM | Ambulatory Blood Pressure Monitor |
|  |  |
|  |  |
| AE | Adverse Event |
|  |  |
|  |  |
| AR | Adverse Reaction |
|  |  |
|  |  |
| BP | Blood Pressure |
|  |  |
|  |  |
| CI | Chief Investigator |
|  |  |
|  |  |
| CMR | Cardiac Magnetic Resonance |
|  |  |
|  |  |
| CT | Computed Tomography |
|  |  |
|  |  |
| CV | Cardiovascular |
|  |  |
|  |  |
| IDMC | Data Safety Monitoring Committee |
|  |  |
|  |  |
| DVD | Digital Versatile Disc |
|  |  |
|  |  |
| ECG | Electrocardiogram |
|  |  |
|  |  |
| eCRF | Electronic Case report form |
|  |  |
|  |  |
| eGFR | Estimated Glomerular Filtration Rate |
|  |  |
|  |  |
| FBC | Full Blood count |
|  |  |
|  |  |
| GCP | Good Clinical Practice |
|  |  |
|  |  |
| GP | General Practitioner |
|  |  |
|  |  |
| IMT | Intima Media Thickness |
|  |  |
|  |  |
| IQCODE | Informant Questionnaire for Cognitive Decline in the Elderly |
|  |  |
|  |  |
| IVRS | Interactive Voice Response System |
|  |  |
|  |  |
| LFT | Liver Function Tests |
|  |  |
|  |  |
| LVH | Left Ventricular Hypertrophy |
|  |  |
|  |  |
| LVM | Left Ventricular Mass |
|  |  |
|  |  |
| MHRA | Medicines and Healthcare Regulatory Authority |
|  |  |
|  |  |
| MoCA | Montreal Cognitive Assessment |
|  |  |
|  |  |
| MRI | Magnetic Resonance Imaging |
|  |  |
|  |  |
| mRS | Modified Rankin scale |
|  |  |
|  |  |
| NIHSS | National Institute of Health Stroke Scale |
|  |  |
|  |  |
| PI | Principle Investigator |
|  |  |

|  | |
| --- | --- |
| **LIST OF ABBREVATIONS (cont.)** | |
|  | |
|  | |
|  |  |
| PV | Pharmacovigilance |
|  |  |
|  |  |
| REC | Research Ethics Committee |
|  |  |
|  |  |
| SAE | Serious Adverse Event |
|  |  |
|  |  |
| SAR | Serious Adverse Reaction |
|  |  |
|  |  |
| SBI | Silent Brain Infarction |
|  |  |
|  |  |
| SmPC | Summary of Product Characteristics |
|  |  |
|  |  |
| SOP | Standard Operating Procedures |
|  |  |
|  |  |
| SRN | Stroke Research Network |
|  |  |
|  |  |
| SSAR | Serious Suspected Adverse Reaction |
|  |  |
|  |  |
| SUSAR | Suspected Unexpected Serious Adverse Reaction |
|  |  |
|  |  |
| TIA | Transient Ischaemic Attack |
|  |  |
|  |  |
| TSC | Trial Steering Committee |
|  |  |
|  |  |
| U+E | Urea and Electrolytes |
|  |  |
|  |  |
| UA | Uric Acid |
|  |  |
|  |  |
| ULN | Upper Limit of Normal |
|  |  |
|  |  |
| WMH | White Matter hyper-intensities |
|  |  |

|  | |
| --- | --- |
| **STUDY SYNOPSIS** | |
|  | |
|  | |
|  |  |
| **Title of Study** | Xanthine Oxidase Inhibition for Improvement of Long-Term Outcomes Following Ischaemic Stroke and Transient Ischaemic Attack (XILO-FIST) |
|  |  |
|  |  |
| *Study Centre* | NHS Greater Glasgow and Clyde, NHS Tayside and associated hospitals of the Managed Clinical Network for Stroke or Stroke Research Network. |
|  |  |
|  |  |
| *Duration of Study* | 5 years |
|  |  |
|  |  |
| *Objectives* | To evaluate the effect of allopurinol 300 mg twice daily on white matter hyper-intensity (WMH) progressionand arterial blood pressure (BP) after ischaemic stroke. |
|  |  |
|  |  |
| *Primary Objective* | To establish whether a two year course of allopurinol 300 mg twice daily reduces WMH progression after ischaemic stroke. |
|  |  |
|  |  |
| *Secondary Objective* | To establish whether allopurinol reduces BP after ischaemic stroke.  To establish whether allopurinol reduces cognitive decline after ischaemic stroke.  To evaluate whether allopurinol reduces vascular event rate after ischaemic stroke. |
|  |  |
|  |  |
| *Main Study Endpoints* | WMH progression. |
|  |  |
|  |  |
|  |  |
|  |  |
|  |  |
|  | Mean day time systolic blood pressure. |
|  |  |
|  |  |
|  | Others include measures of cognition and vascular event rate.  . |
|  |  |
|  |  |
| *Rationale* | A short course of allopurinol has been shown to improve measures of vascular health in a variety of subgroups and after stroke. It is unclear whether these benefits are prolonged and whether they translate into benefit on robust surrogate markers of risk or on clinical endpoints. |
|  |  |
|  |  |
| *Methodology* | Randomised double blind placebo controlled clinical trial, with 3 nested sub-studies. |
|  |  |
|  |  |
| *Sample Size* | 232 people per group (allopurinol vs. placebo) |
|  |  |
|  |  |
| *Screening* | Case note review of in-patient / outpatient attendees to the Acute Stroke Service by clinicians. A full log will be maintained. |
|  |  |
|  |  |
| *Randomisation* | Subjects will be allocated 1:1 to allopurinol 300 mg twice daily or placebo using a mixed randomisation and minimisation algorithm, performed via web portal with telephone backup |
|  |  |

|  | | | |
| --- | --- | --- | --- |
| **STUDY SYNOPSIS (cont.)** | | | |
|  | | | |
|  | | | |
|  | |  | |
| *Inclusion Criteria* | | 1. Ischaemic Stroke. 2. Age greater than 50 years. 3. Ischaemic lesion on brain imaging in relevant anatomical territory in patients with transient ischaemic attack. 4. Consent within one month of stroke. | |
|  | |  | |
|  | |  | |
| *Exclusion Criteria* | | 1. Modified Rankin scale score of 5 (at end of the possible enrolment window of one month after stroke). 2. Diagnosis of dementia (defined as a documented diagnosis or a screening IQCODE (Informant Questionnaire for Cognitive Decline in the Elderly) score of 3.6 or more). 3. Cognitive impairment deemed sufficient to compromise capacity to consent or to comply with the protocol (in the opinion of the local investigator). 4. Dependent on daily help from others for basic or instrumental activities of daily living prior to stroke (defined as assistance needed with toileting, walking or dressing). 5. Significant co-morbidity or frailty likely to cause death within 24 months or likely to make adherence to study protocol difficult for participant (in the opinion of the local investigator). 6. Contra-indication to or indication for administration of allopurinol (as detailed in Summary of Product Characteristics on the XILO-FIST web portal an in trial master file). 7. Concurrent azathioprine, 6-mercaptopurine therapy, other cytotoxic therapies, cyclosporin, theophylline and didanosine. 8. Significant hepatic impairment (defined as serum bilirubin, AST or ALT greater than three times upper limit of normal (ULN)). 9. Estimated Glomerular Filtration Rate < 30 mls/min 10. Contraindication to MRI scanning. 11. Women of childbearing potential 12. Prisoners. 13. Active participation in another CTIMP or device trial or participation within the past month. 14. Korean, Han Chinese or Thai descent unless negative HLA-B*5801 status is known | |
|  | |  | |
|  | |  | |
| *Product, Dose, Modes of Administration* | | Allopurinol (generic) 300 mg twice daily orally. | |
|  | |  | |
|  | |  | |
| *Duration of Treatment* | | 2 years (104 weeks). | |
|  | |  | |
|  | |  | |
| *Statistical Analysis* | | A generalized linear regression model, with appropriate link and variance function, will be used to model WMH progression in relation to treatment, variables used in the minimisation algorithm, and other baseline characteristics found to be associated with WMH progression during blinded analysis. | |
|  | |  | |
|  | |  | |
| *Sub-study Details* | | One left ventricular hypertrophy sub-study and one carotid plaque sub-analysis will be nested within the main trial. | |
|  | |  | |

| **DETAILED PARTICIPANT SCHEDULE** | | | | | | | | | |
| --- | --- | --- | --- | --- | --- | --- | --- | --- | --- |
| **Activity** | **Run-In Phase** | | **Treatment Phase** | | | | | | |
|  | Day 0 | Week 4 | Week 0 | Week 4 | Week 13 | Week 26 | Week 52 | Week 78 | Week 104 |
| Review Eligibility | ✓ | ✓ |  |  |  |  |  |  |  |
| Informed Consent | ✓ |  |  |  |  |  |  |  |  |
| Optimise Preventative Therapy | ✓ |  |  |  |  |  |  |  |  |
| Clinical Evaluation * | ✓ | ✓ |  | ✓ | ✓ | ✓ | ✓ |  | ✓ |
| Safety Blood Tests # | ✓ | ✓ |  | ✓ | ✓ | ✓ | ✓ |  | ✓ |
| Blood for Uric Acid Level |  | ✓ |  |  |  |  |  |  | ✓ |
| Blood / Urine for Biobanking |  | ✓ |  |  |  |  |  |  | ✓ |
| ECG |  | ✓ |  |  |  |  |  |  | ✓ |
| Echocardiography ‡ |  | ✓ |  |  |  |  |  |  |  |
| Determine Cardiac Sub-study Eligibility |  | ✓ |  |  |  |  |  |  |  |
| MRI Brain (± carotid MRI) |  | ✓ |  |  |  |  |  |  | ✓ |
| ABPM |  | ✓ |  | ✓ |  |  |  |  | ✓ |
| Cardiac MRI ‡, n=100 |  | ✓ |  |  |  |  |  |  | ✓ |
| Detailed Cognitive Function Evaluation |  | ✓ |  |  |  |  |  |  | ✓ |
| Assessment of run in completion |  | ✓ |  |  |  |  |  |  |  |
| Randomisation |  |  | ✓ |  |  |  |  |  |  |
| Dispense |  |  | ✓ | ✓ | ✓ | ✓ | ✓ | ✓ |  |
| Return / Count IMP |  |  |  | ✓ | ✓ | ✓ | ✓ | ✓ | ✓ |
| Adverse Event Review |  |  | ✓ | ✓ | ✓ | ✓ | ✓ | ✓ | ✓ |
| *includes measures of stroke severity at week 4, modified Rankin scale score and MoCA at week 52 and blood pressure at all visits except week 78 and weight at week 104).  # includes FBC, U+E, LFTs. ‡ sub-study eligible participants only (echo may not be needed if ECG eligible). IMP = investigational medicinal product. ABPM = ambulatory blood pressure monitoring.  Note that the run-in week 4 and treatment phase week 0 visits can take place concurrently.  Participants will also be contacted by telephone at week 105, which marks the end of the study. | | | | | | | | | |

|  |
| --- |
| **INTRODUCTION** |
|  |
|  |
|  |
| **BACKGROUND** |
|  |
|  |
| New strategies are needed to improve long-term outcomes after ischaemic stroke or transient ischaemic attack (TIA). Approximately 13% of participants suffered recurrent stroke in recent secondary preventative trials^^[[1]](#endnote-1)^^, 40% of patients with TIA experience recurrent cardiovascular (CV) events during long-term follow up and there is an additional substantial burden from incident post-stroke dementia (~ 10% after first stroke and higher still after recurrent events)^^[[2]](#endnote-2)^^, cognitive decline (over 30%)^^[[3]](#endnote-3)^^ and decline in physical function. Improving these outcomes is a recognised priority area for stroke research (as identified by stroke survivors through the recent James Lind Alliance priority setting workshops^^[[4]](#endnote-4)^^).  Such adverse outcomes are particularly common in those with brain white matter hyper-intensities (WMH) on brain magnetic resonance imaging (MRI)^^[[5]](#endnote-5)^^. WMH are seen in as many as 90% of patients with ischaemic stroke^^[[6]](#endnote-6)^,^^^[[7]](#endnote-7)^^, are at least moderately severe in 50%^6^ and such ‘severe’ WMH are associated with substantially higher stroke recurrence rates (43% in one study)^6^, death^^[[8]](#endnote-8)^^ and increased cognitive and physical decline. The burden of WMH increases during longitudinal follow up and this is associated with increased incident stroke, dementia and cognitive decline^5^. In the longitudinal population based Rotterdam scan study, 39% of elderly participants had WMH progression (over a mean period of 3.4 years)^^[[9]](#endnote-9)^^, as did 50% in the recent PROFeSS MRI sub-study (over 2 years)^7^ and 74% (over 3 years) in the Leukoariosis and Disability study (LADIS)^^[[10]](#endnote-10)^^.Similarly, silent brain infarction (SBI) is also associated with recurrent stroke and 14% developed incident infarcts on brain MRI in the Rotterdam scan study^9^. Thus, treatments that reduce WMH progression and incident silent brain infarction could have potentially profound effects on a variety of outcomes after stroke including cognition, functional outcome and recurrent stroke.  The pathological basis for WMH development and progression is poorly understood. Post mortem studies show presence of varied pathologies including demyelination, infarction, arteriosclerosis and breakdown of the blood-brain barrier. Key risk factors for development and progression of WMH are age, arterial hypertension and previous stroke^9^ and associations with other cardiovascular risk factors and left ventricular hypertrophy (LVH) have been demonstrated^^[[11]](#endnote-11)^^. Blood pressure (BP) lowering reduces WMH progression, as demonstrated by the PROGRESS MRI sub-study^^[[12]](#endnote-12)^^. In the PROFeSS MRI sub-study WMH progression was unaffected by the angiotensin receptor blocker telmisartan^7^ but unlike PROGRESS, there was no significant difference in BP between groups. In addition, WMH are less clearly related to hypertension in older patients with established cardiovascular disease^^[[13]](#endnote-13)^^ meaning that novel strategies which reduce WMH progression and SBI would be particularly promising in this group.  The association between WMH and LVH is of particular interest; it appears independent of arterial BP^^[[14]](#endnote-14)^,^[[15]](#endnote-15)^^ and may be mediated by aortic stiffness^^[[16]](#endnote-16)^^. There are additional potential mechanisms for this association (e.g., LVH is the strongest predictor of left atrial appendage thrombi, stronger than any left atrial parameter)^^[[17]](#endnote-17)^^. Regression of LVH is associated with reduced risk of stroke. In a recent meta-analysis of 14 studies in 12,809 patients, LVH regression was independently associated with a 25% reduction in future strokes, whereas the composite endpoint of CV events/mortality was only 15% lower^^[[18]](#endnote-18)^^. Similar findings were seen in the LIFE echo sub-study which utilised measures of left ventricular mass (LVM)^^[[19]](#endnote-19)^^. LVH regression is thus a promising therapeutic target in devising new ways to prevent strokes, especially if the same treatment were found to reduce WMH. |
|  |

|  |
| --- |
| **STUDY HYPOTHESIS** |
|  |
|  |
| We hypothesise that allopurinol will improve long-term outcomes after ischaemic stroke by (in comparison to placebo) 1) reducing progression of WMH and SBI (measured using MRI), that are associated with cognitive decline and recurrent stroke, 2) cause LVH regression through a BP-independent mechanism, which is associated with stroke risk, 3) will reduce arterial BP, which is associated with stroke risk and cognitive decline and 4) reduce carotid artery plaque volume. |
|  |
|  |
|  |
| **PILOT DATA TO SUPPORT BENEFICIAL EFFECTS OF ALLOPURINOL / SYSTEMATIC REVIEW AND META-ANALYSIS** |
|  |
|  |
| Allopurinol, a drug commonly prescribed for the prophylaxis of gout, inhibits activity of xanthine oxidase leading to reduction in both serum uric acid (UA) and oxidative stress via reduced superoxide anion production. Higher serum UA is associated with increased risk of CV disease^^[[20]](#endnote-20)^^, with adverse outcomes after ischaemic stroke^^[[21]](#endnote-21)^,^[[22]](#endnote-22)^^ and with cognitive impairment^^[[23]](#endnote-23)^^. Allopurinol reduces UA by as much as 60% in patients with cardiovascular disease, with acceptable safety^^[[24]](#endnote-24)^,^^^[[25]](#endnote-25)^^ and may deliver benefits independent of uric acid reduction^^[[26]](#endnote-26)^^. The Glasgow group have shown that allopurinol 300 mg per day improves cerebral nitric oxide bioavailability in patients with type 2 diabetes^^[[27]](#endnote-27)^^, reduces markers of inflammation after ischaemic stroke^25^ and have just completed a one year pilot randomised double blind placebo-controlled study of allopurinol 300 mg per day in 80 patients with ischaemic stroke. The primary endpoint was change in carotid intima-media thickness (IMT) at one year, an accepted surrogate marker of large artery disease-associated risk, and secondary endpoints included measures of arterial stiffness and endothelial function. There were no safety concerns. A significant difference in the change in IMT at one year (between group difference of 0.09 mm, 95% CI 0.15 to 0.02) and a reduction in central aortic systolic BP at one year (6.6 mmHg, 95% CI -13.0 to -0.3, measured using applanation tonometry) were found following allopurinol**.** This study provides evidence of a sustained effect of allopurinol after ischaemic stroke. Peripheral blood pressure was also lowered. Researchers from the University of Dundee have shown that a 300 mg twice daily dose of allopurinol may be more effective than 300 mg once daily ^26^ and that this dose causes regression of LVH in patients with diabetes, renal impairment^^[[28]](#endnote-28)^^ and angina and that it reduces myocardial ischaemia in patients with angina^^[[29]](#endnote-29)^^.They have also shown that allopurinol consistently reduces augmentation index as a measure of LV afterload, including after stroke^^[[30]](#endnote-30)^^. While allopurinol has been reported to lower arterial BP^^[[31]](#endnote-31)^^ in adolescents and by 3.3 / 1.3 mmHg in a recent systematic review and meta-analysis of small studies^^[[32]](#endnote-32)^^, no effect on BP has been clearly demonstrated in adults where no trial has been specifically designed to test this hypothesis. In our systematic review and meta-analysis^^[[33]](#endnote-33)^^ of 38 studies, allopurinol significantly improved a variety of measures including brachial artery flow mediated dilatation, a measure of endothelial function.  In summary, allopurinol has been shown to have effects on a measure of pre-atherosclerosis (IMT), large artery function and afterload, small vessel function within the brain, myocardial ischaemia, peripheral arterial function and on left ventricular hypertrophy and may reduce arterial blood pressure. |
|  |

|  |
| --- |
| **TOLERABILITY OF ALLOPURINOL IN PATIENTS WITH RECENT STROKE** |
|  |
|  |
| Allopurinol is generally well tolerated and has been in use for nearly half a century. Side-effects typically comprise gastro-intestinal upset (uncommon) and rashes (in ~ 2% of patients). Hypersensitivity reactions rarely occur (less than 1 in 1000 cases)^^[[34]](#endnote-34)^^ and Stevens Johnson syndrome, although reported, is estimated to affect 0.008% of allopurinol users per year^^[[35]](#endnote-35)^^.  We have demonstrated the safety and tolerability of allopurinol daily in clinical trials of patients with recent ischaemic stroke. In our first study we randomised 50 such patients to receive either allopurinol 300 mg or 100 mg, or placebo for six weeks. No serious adverse events (SAEs) occurred^25^. In our second study we also found no SAEs attributable to therapy^26^. We have most recently completed a one year pilot randomised double blind placebo-controlled study of allopurinol 300 mg per day in 80 patients with ischaemic stroke. There were no treatment related SAEs.  A dose of 600 mg daily has been studied in numerous recent trials in patients with ischaemic heart disease and patients at high cardiovascular risk. This dose appears to have greater cardiovascular benefits than lower doses^26,29^. In these trials there were no safety concerns, even though some had renal dysfunction. The risk of severe hypersensitivity reactions seems to be confined to the first weeks of treatment. We have therefore chose to study a lower dose initially, to instruct participants to stop medication immediately if they develop a rash and will not up-titrate participants whose renal function is poor (defined as estimated glomerular filtration rate (eGFR)<60 mL/min) or when there are concerns regarding hepatic function. |
|  |
|  |
|  |
| **SUMMARY OF RISK ASSESSMENT** |
|  |
|  |
| As outlined above, there is sufficient data to warrant further clinical trials of allopurinol use after stroke. We anticipate a small number of cases of rash and have incorporated stringent criteria and participant monitoring to reduce the risk of encountering more serious adverse effects.  It has also not been possible to obtain an exact active-placebo tablet match but the objective nature of the primary end-points and the use of study specific arrangements to maintain blinding address this potential risk. We believe that available data show the risk:benefit ratio is in favour of further evaluation.    A separate risk assessment of the trial will be conducted by the sponsor. |
|  |

|  |
| --- |
| **TRIAL OBJECTIVES AND PURPOSE OF CLINICAL TRIAL** |
|  |
|  |
|  |
| This trial programme brings together two groups who have been investigating xanthine oxidase inhibition for the treatment of cardiac disease and stroke. It will establish whether, after ischaemic stroke, allopurinol; 1) reduces WMH progression and silent brain infarction; 2) induces regression of LVH (via a sub-study); and 3) lowers arterial BP. It will also (4) assess clinical outcomes (measures of cognition and vascular event rate) which will aid design of future definitive clinical endpoint studies. The study will definitively establish whether allopurinol has beneficial effects on three powerful surrogate endpoints for adverse outcomes after stroke and thereby allow a pivotal large scale clinical endpoint study to be designed, funded and implemented. |
|  |

|  |
| --- |
| **STUDY DESIGN** |
|  |
|  |
|  |
| This study will be performed according to the Research Governance Framework for Health and Community Care (Second edition, 2006) and The Medicines for Human Use (Clinical Trials) Regulations, 2004 SI 2004:1031 (as amended). All investigators and key trial personnel will undergo Good Clinical Practice (GCP) training every 2 years.  The trial design is detailed below and is summarised in the flow chart and in table 1. The trial is a multi-centre randomised, double-blind placebo controlled study to investigate whether two years allopurinol 300 mg twice daily leads to a reduction in WMH progression rate in 464 participants with recent stroke. The trial will incorporate ambulatory BP monitoring and cardiac sub-studies. It will involve major stroke centres in NHS GGC, Tayside and sites of the UK stroke research network (SRN). |
|  |
|  |
|  |
| **STUDY POPULATION** |
|  |
|  |
| The study will involve participants aged over 50 years with an ischaemic stroke who meet the inclusion criteria below and who have none of the specified exclusion criteria. All will give full informed consent. |
|  |
|  |
|  |
| **MAIN TRIAL INCLUSION CRITERIA** |
|  |
|  |
| 1. Ischaemic Stroke/ Ischaemic lesion on brain imaging in relevant anatomical territory in patients with transient ischaemic attack 2. Age greater than 50 years. 3. Consent within one month of stroke.   *Ischaemic stroke will be diagnosed by a stroke specialist and defined as a focal neurological event lasting more than 24 hours or symptoms lasting less than 24 hours with positive diffusion weighted imaging or a corresponding lesion on CT. This inclusion criterion will be verified by local investigators following image review and this will be recorded in the patient record and on the electronic case report form (eCRF).* |
|  |

|  |
| --- |
| **MAIN TRIAL EXCLUSION CRITERIA** |
|  |
|  |
| 1. Modified Rankin scale score of 5(at end of the possible enrolment window of one month after stroke). 2. Diagnosis of dementia (defined as a documented diagnosis or a screening IQCODE (Informant Questionnaire for Cognitive Decline in the Elderly) score of 3.6 or more). 3. Cognitive impairment deemed sufficient to compromise capacity to consent or to comply with the protocol (in the opinion of the local investigator). 4. Dependent on daily help from others for basic or instrumental activities of daily living prior to stroke (defined as assistance needed with toileting, walking or dressing). 5. Significant co-morbidity or frailty likely to cause death within 24 months or likely to make adherence to study protocol difficult for participant (in the opinion of the local investigator). 6. Contra-indication to or indication for administration of allopurinol or febuxostat (as detailed in the reference Summary of Product Characteristics on the XILO-FIST web portal and in trial master file) or placebo ingredients (hereditary galactose intolerances). 7. Concurrent azathioprine, 6-mercaptopurine therapy, other cytotoxic therapies, cyclosporin, theophylline and didanosine. 8. Significant hepatic impairment (defined as serum bilirubin, AST or ALT greater than three times upper limit of normal (ULN)). 9. eGFR < 30 mL/min 10. Contraindication to MRI scanning. 11. Women of childbearing potential. 12. Prisoners. 13. Active participation in another CTIMP or device trial or participation within the past month. 14. Korean, Han Chinese or Thai descent unless negative HLA-B*5801 status is known.   *Women of non-childbearing potential are defined as those who have no uterus, ligation of the fallopian tubes, or permanent cessation of ovarian function due to ovarian failure or surgical removal of the ovaries. A woman is also presumed to be infertile due to natural causes if she has been amenorrheic for greater than 12 months and has an FSH greater than 40 IU/L.* |
|  |

|  |
| --- |
| **ADDITIONAL SUB-STUDY INCLUSION CRITERIA** |
|  |
|  |
|  |
| **CARDIAC MRI SUB-STUDY (Terminated)** |
|  |
|  |
| In addition to meeting all above criteria participants must meet the following inclusion criteria:   1. Sinus rhythm on 12 lead ECG 2. Presence of mild, moderate or severe LVH defined as one or more of;    1. LVH on ECH on either the Sokolow-Lyon or Cornell voltage criteria    2. Posterior or septal wall thickness of >11mm    3. Increased LV mass defined as baseline LVM index of > 115g/m^2^ (men) or > 95g/m^2^ (women) OR LV mass >162g (women) or >224g (men) on screening echocardiography.   A maximum of 100 participants will be recruited to this sub-study. Recruitment to this sub study will stop when recruitment to the main study finishes. |
|  |
|  |
|  |
| **CAROTID MRI SUB-STUDY** |
|  |
|  |
| There are no further eligibility criteria for this study. To be included, sites must have access to required coils for carotid MRI. |
|  |
|  |
|  |
| **IDENTIFICATION OF PARTICIPANTS AND CONSENT** |
|  |
|  |
| Potential participants will be identified (by case note review by a member of the clinical team or by their attending Doctor) whilst in-patients in an acute stroke unit or in a cerebrovascular out-patient clinic. Following identification, potential participants will be approached in person and asked whether they would wish to consider taking part in the trial. Those who are willing to hear more will be given a participant information sheet and a date (at least 24 hours later) arranged for further discussion with a member of the research team. Eligibility will be confirmed by a medically qualified investigator.  At this second meeting, subjects will be asked if they have any questions and those who wish to participate will be asked to sign the consent form. Two copies will be signed (one each for the participant and the site file) and a copy of the signed consent form will be inserted into the patient’s record.  Consent will be taken by one of the investigators or by a study research nurse (in which case it will be countersigned by an investigator).  Consent for the cardiac sub-study will be given separately.  If the week 104 visit is expected to be delayed by more than 2 weeks due to restrictions required to combat the Covid-19 pandemic, the participant will be asked whether they are willing to remain on IMP and remain in the study for a maximum of 6-months to allow completion of the week 104 visit. Given the restrictions are most likely to involve a need to reduce person to person contact, on balance of risk, it is not in the participants interest to seek face to face consent and record this in writing. We will ask participants to confirm ongoing participation by telephone or email. This will be documented in their case record. |
|  |

|  |
| --- |
| **VISIT SCHEDULE** |
|  |
|  |
|  |
| The study will comprise a 4-week run in phase and a 104 week treatment phase. Each visit has a ± 7 day window either side of the scheduled date during which it can be completed. From March 2020 the week 104 visit can be performed 3 months earlier than week 104 and up-to 6 months later if required due to restrictions required to combat the Covid-19 pandemic. |
|  |
|  |
|  |
| **RUN-IN PHASE** |
|  |
|  |
| This will comprise a day 0 enrolment visit and a week 4 baseline assessment visit. The aim of this period is to optimise secondary preventative treatments, determine sub-study eligibility and complete baseline assessments. In order to successfully complete the run in phase participants must have had a medication review, completed baseline data collection and tolerated brain MRI. Those who do not successfully complete this visit will be classed as screen failures. |
|  |
|  |
|  |
| Enrolment Visit (Day 0) |
|  |
|  |
| This will involve review of eligibility and consent, a clinical assessment (including measurement of BP, National Institute of Health Stroke Scale (NIHSS) score, modified Rankin Scale (mRS) score), safety blood tests, a management plan for optimisation of preventative treatments and scheduling of baseline assessment tests.  Optimisation of preventative treatments will include ensuring participants are (or it has at least it has been attempted);1) taking optimal anti-platelet therapy (clopidogrel 75 mg per day or aspirin and dipyridamole in combination) or anticoagulation therapy if indicated, 2) taking cholesterol lowering therapy at a dose at least equivalent to simvastatin 40 mg daily or to a total cholesterol level of <5 mmol/L and 3) have a blood pressure of <140/85 mmHg or if not be taking at least two blood pressure lowering drugs (BP will be measured by specialists nurses and be defined as the mean of 3 readings and a target of 130/80 mmHg will be used for patients with diabetes). This will be performed in conjunction with the G.P. |
|  |
|  |
|  |
| Baseline Assessment Visit (Week 4) |
|  |
|  |
| This will include a clinical evaluation (measurement of BP), blood tests for safety and Biobanking, a blood test for serum uric acid (centrally analysed) a urine sample for Biobanking, echocardiography (if not already performed and if site is taking part in the cardiac sub-study), an ABPM, electrocardiography (ECG), brain MRI, assessment of cognitive function and assessment of eligibility for the cardiac sub-study. Participants who are eligible and willing to enter the cardiac sub-study will undergo an additional cardiac MRI scan. The first 90 participants at selected sites will also undergo carotid plaque MRI (as part of the brain MRI scan).  If a participant does not complete the run in phase, they will be classed as a screen failure. A participant can take part in the main study and fail the sub-study screening (e.g. have LV mass less than required for cardiac sub-study). |
|  |

|  |
| --- |
| **TREATMENT PHASE** |
|  |
|  |
|  |
| Randomisation (Week 0 Visit) |
|  |
|  |
| This is likely to occur concurrently with the run in phase week 4 visit. Participants who successfully complete the run in phase will be randomised (1:1) to receive either allopurinol 300mg twice daily (300 mg once daily for first month) or placebo orally for 104 weeks. Randomisation marks the start of the treatment phase and participants will then be issued with their study medication. |
|  |
|  |
|  |
| Week 4 Visit |
|  |
|  |
| Participants will attend their local study facility. Clinic blood pressure will be measured and blood will be drawn for an interim safety check. A checklist will be completed to identify any adverse events or recurrent vascular events. An ABPM will be performed where patients have tolerated the baseline ABPM. IMP will be dispensed and a capsule count performed. |
|  |
|  |
|  |
| Week 13 Visit |
|  |
|  |
| Participants will attend their local study facility. Clinic blood pressure will be measured and blood will be drawn for an interim safety check. A checklist will be completed to identify any adverse events or recurrent vascular events. IMP will be dispensed and an IMP count performed. |
|  |
|  |
|  |
| Week 26 Visit |
|  |
|  |
| Participants will attend their local study facility. Clinic blood pressure will be measured and blood will be drawn for an interim safety check. A checklist will be completed to identify any adverse events or recurrent vascular events. IMP will be dispensed and an IMP count performed. |
|  |
|  |
|  |
| Week 52 Visit |
|  |
|  |
| Participants will attend their local study facility. Clinic blood pressure will be measured and blood will be drawn for an interim safety check.. A checklist will be completed to identify any adverse events or recurrent vascular events. A mRS score and Montreal Cognitive Assessment will also be performed. IMP will be dispensed and an IMP count performed. |
|  |
|  |
|  |
| Week 78 Visit |
|  |
|  |
| Participants will either attend their local study facility. A checklist will be completed to identify any adverse events or recurrent vascular events. IMP will be dispensed and an IMP count performed. If required, from March 2020 this visit can be performed by telephone and IMP sent to participants if required due to restrictions required to combat the Covid-19 pandemic. |
|  |

|  |
| --- |
| Week 104 Visit |
|  |
|  |
| Participants will attend their local study facility. Blood pressure will be measured and blood will be drawn for a final safety check and serum uric acid (centrally analysed). A checklist will be completed to identify any adverse events or recurrent vascular events. An IMP count will be performed.  A mRS score will be assigned. Blood tests for Biobanking, a urine sample for Biobanking, electrocardiography (ECG), brain MRI (± carotid MRI) and assessment of cognitive function will be performed. An ABPM will be performed where patients have tolerated the baseline ABPM. Participants in the cardiac sub-study will undergo an additional cardiac MRI.  NOTE: the Cardiac sub-study did not recruit enough participants to determine the secondary endpoint therefore Participants in the cardiac sub study will NOT undergo an additional cardiac MRI at this visit.  Please refer to 7T MRI sub study Protocol for additional information regarding recruitment to and assessment of participants to this sub-study i.e a 7T MRI scan carried out following the assessments relating to main study described above.  From March 2020 the week 104 visit can be performed 3 months earlier than week 104 and up-to 6 months later if required due to restrictions required to combat the Covid-19 pandemic. If it is anticipated that the week 104 visit will be performed more than 2-weeks after week 104, more IMP should be dispensed and sent to the participant. This can be done for a maximum of 6 months at which point the week 104 visit must be performed and IMP will cease. If the visit cannot be conducted in full due to these restrictions (ie, if face to face contact is not possible), the participant should be telephoned to confirm vital status and to screen for adverse and serious adverse events. |
|  |
|  |
|  |
| Week 105 Telephone Call |
|  |
|  |
| Participants will be contacted by telephone and will be assessed for the presence of any adverse events. Any findings will be followed up accordingly.  This telephone call marks the end of participation in the study. |
|  |
|  |
|  |
| **CARDIAC SUB-STUDY VISIT SCHEDULE** |
|  |
|  |
| Participants will undergo additional cardiac 3T MRI using our validated and previously published imaging protocols (28) (further details below) at baseline. This will however not be repeated at two years due to insufficient numbers recruited to assess the endpoint of this study i.e. to determine whether allopurinol reduces LVH after ischaemic stroke. |
|  |
|  |
|  |
| **SUB-ANALYSIS VISIT SCHEDULE** |
|  |
|  |
| Participants will undergo additional carotid MRI sequences during their brain MRI (further details below). This will be performed at baseline and repeated at week 104. |
|  |

|  |
| --- |
| **BRAIN MRI IMAGING PROTOCOL** |
|  |
|  |
| A detailed imaging and image analysis protocol will be agreed during the trial start-up phase and will be detailed in a separate document. This will ensure sequences are harmonised across a common standard, give detailed information on acquisition parameters, instructions for radiographers, confirm details of anonymisation and coding of participant data, outline required quality assurance procedures and confirm exact details of the upload and distribution process.  All participants should be scanned on the same scanner at the baseline and follow-up visit. In the rare instance where this is not possible, scans should still be performed but this will be noted in the eCRF.  Brain MRI will be performed using 1.5 or 3T MRI on all sites. There will be a core imaging battery required for all participants. This will entail 3D-T1 weighted imaging, T2-weighted imaging and fluid attenuation inversion recovery (FLAIR), diffusion weighted imaging and susceptibility weighted imaging (or T2* gradient echo sequences where this is not available).Typical sequence parameters are; T2- weighted imaging: TR = 6100ms , TE = 103ms , flip angle = 150 degrees, slice thickness = 3mm, number of averages = 1, voxel size = 0.6x0.5x3.0mm, field of view = 220mm. FLAIR imaging:TI = 2500ms, TR =9000ms , TE =94ms , flip angle = 150degrees, slice thickness = 4mm, number of averages = 1, voxel size = 0.9x0.9x4mm, field of view = 230mm. 3D-T1 weighted imaging: TR = 1900ms , TE = 2.45ms, flip angle = 9 degrees, slice thickness = 1mm, number of averages = 1, voxel size = 1x1x1mm, field of view = 250mm. Susceptibility weighted imaging: TR =27ms, TE =20ms, flip angle = 15mm slice thickness = 1.5mm, number of averages = 1, voxel size = 0.9x0.9x1.5mm, field of view = 230mm.  An extended imaging battery can be performed and this will incorporate diffusion tensor imaging, arterial spin labelling imaging and quantitative T2 imaging. This can be done by any site but is not a mandatory part of the trial protocol. |
|  |
|  |
|  |
| **CAROTID MRI IMAGING PROTOCOLS** |
|  |
|  |
| Carotid imaging will be performed where sites have the capability and participants will undergo carotid MRI. This will include time of flight carotid angiography, black blood T1, black blood T2 and black blood proton density imaging of the carotid arteries. This will be performed during the main brain imaging session at baseline and 2 years (total (brain and carotid) image acquisition time ~ 55 minutes for these participants). All participants should be scanned on the same scanner at the baseline and follow-up visit. In the rare instance where this is not possible, scans should still be performed. |
|  |

|  |
| --- |
| **CARDIAC MRI IMAGING PROTOCOLS** |
|  |
|  |
| Baseline CMR examinations will be performed on 3T scanners using dedicated phased array cardiac coils in the cardiac sub-study participants. A detailed imaging and image analysis protocol will be agreed during the trial start-up phase and will be detailed in a separate document. This will ensure sequences are harmonised across a common standard, give detailed information on acquisition parameters, instructions for radiographers, confirm details of anonymisation and coding of participant data, outline required quality assurance procedures and confirm exact details of the upload and distribution process.  Following localiser acquisitions, and two-chamber and four-chamber cine gradient echo sequences , short-axis plane images of the left atrium and left ventricle will be acquired to the apex using a combination of body matrix and spine matrix RF coils and a 2D breath-hold segmented TrueFisp CINE sequence with prospective cardiac gating. The imaging parameters will be in the same range on each scanner, (ie TR 39.06 ms, TE 1.2 ms and flip angle 60u) and the scan time for each acquisition minimised by use of a parallel imaging acceleration factor of two. A series of 6-mm thick images (with 1.5-mm slice gap) covering the entire left atrium and ventricle will be acquired for each subject with an in-plane pixel matrix of 1286x192 over an optimal (patient-size-dependent) field of view ranging from 340–450 mm. In addition, with little additional scanning time we will assess aortic vascular compliance and pulse wave velocity using cine transverse thoracic aortic gated high resolution steady state free precession acquisitions. The imaging parameters include slice thickness=8 mm, TR=7 ms, TE=4 ms, no. averages=1, no. phases=128, velocity encoding= 150 cm/s, bandwidth/pixel=340 Hz, flip angle=15°, field of view (FOV)=320 × 320 mm2, matrix=256 × 256, imaging with time ≈2 min (depending on heart rate). |
|  |
|  |
|  |
| **AMBULATORY BLOOD PRESSURE MONITORING** |
|  |
|  |
| This will be performed at baseline, week 4 and at week 104where tolerated. The British Hypertension Society Standard Operating Procedures, checklists and patient information leaflets and diaries will be used (these are shown in the ABPM accompanying documentation and at http://www.bhsoc.org/resources/abpm/).  Participants will undergo ambulatory blood pressure monitoring for 24 hours using the SpaceLabs Ultralight Ambulatory Blood Pressure Monitor. This will be set to take readings every 30 minutes during daytime (0800 h–2159 h) and every 60 minutes during night-time (2200 h–0759 h). |
|  |
|  |
|  |
| **CLINIC BLOOD PRESSURE MEASUREMENT** |
|  |
|  |
| Clinic blood pressures will be measured using an oscillometric device after 5 minutes rest (in the sitting position). Three readings will be taken and the mean of the second and third will be used. At the first visit it will be checked in both arms and the arm with the highest reading will be used thereafter. |
|  |

|  |
| --- |
| **BRAIN MRI UPLOAD AND DISTRIBUTION AND REVIEW** |
|  |
|  |
|  |
| **MRI IMAGE UPLOAD** |
|  |
|  |
| Brain MRI scans (in DICOM and anonymised format) will be uploaded via the eCRF. The study Chief Investigator (JD), Image Reviewer (DD) and/or delegate will be notified by email when an MRI is uploaded. Where upload has been attempted and has been unsuccessful, the local site and JD / DD and/or delegate will be notified by email that a further attempt is needed. In the event of digital upload not being possible, anonymised DICOM files will be sent via DVD and will then be uploaded via the eCRF by the co-ordinating centre. |
|  |
|  |
|  |
| **INITIAL QUALITY REVIEW** |
|  |
|  |
| For study related analyses, anonymised scans will managed and viewed via the eCRF blinded to treatment allocation and other clinical data. Within 2 working days of receipt of either a baseline or follow-up scan, the images will be visually reviewed for quality by either JD, DD and/or delegate or a nominated deputy who will adjudicate if all sequences are present and of acceptable quality for the primary endpoint data to be gathered. Acceptable images will be then be made available for image analysis. Where they are not, a telephone discussion will be held with the local site investigator to explore whether a repeat MRI scan is feasible. An acceptable scan can be released for endpoint review. |
|  |
|  |
|  |
| **BASELINE SCAN REVIEW** |
|  |
|  |
| Once a baseline scan has been released for initial endpoint review, it will be assigned to 2 trained and certified raters (JD, DD and/or delegate). The Fazeka’s scale and Scheltens scales will be applied. Where there is disagreement on these visual WMH scales, the study CI and Imaging Reviewer (DD) will be notified by email. These cases will be reviewed in an imaging adjudication meeting (attended by JD, DD and/or delegate). A 3^rd^ reviewer will be available if consensus cannot be reached. Exact parameters that define disagreement will be defined during development of the separate image analysis protocols. |
|  |
|  |
|  |
| **FOLLOW-UP SCAN REVIEW** |
|  |
|  |
| Once a follow-up scan has been released for initial endpoint review, it will be assigned to 2 trained and certified raters (JD, DD and/or delegate). The Fazeka’s scale and Scheltens scales will be applied by both raters. Where there is disagreement on these visual WMH scales, the study CI and Imaging Reviewer (DD) will be notified by email. These cases will be reviewed in an imaging adjudication meeting (attended by JD, DD and/or delegate). A 3^rd^ reviewer will be available if consensus cannot be reached. Exact parameters that define disagreement will be defined during development of the separate image analysis protocols. |
|  |
|  |
|  |
| **SIDE BY SIDE SCAN REVIEW** |
|  |
|  |
| Once a follow-up scan has been uploaded and the initial endpoint review has been performed, the baseline and follow-up scans will be assigned to 2 trained and certified raters (JD, DD and/or delegate). The Rotterdam Progression Scale and the Schmidt’s scale will be applied. Where there is disagreement on these visual WMH scales, the study CI and Imaging Reviewer (DD) will be notified by email. These cases will be reviewed in an imaging adjudication meeting (attended by JD, DD and/or delegate). A 3^rd^ reviewer will be available if consensus cannot be reached. Exact parameters that define disagreement will be defined during development of the separate image analysis protocols. |
|  |

|  |
| --- |
| **BRAIN MRI RATER TRAINING** |
|  |
|  |
| All raters will undergo training using existing materials for use of visual rating scales. We will then establish interobserver variability for the visual rating scales using a series of 20 brain MRI scans taken from our pilot study. This will also allow us to finalise scoring rules and define parameters for adjudication review. Agreement will be monitored throughout the study. |
|  |
|  |
|  |
| **BRAIN MRI IMAGE ANALYSIS PROTOCOLS** |
|  |
|  |
| A detailed image analysis protocol will be agreed during the trial start-up phase and will be detailed in a separate document. Image analysis will be performed from a dedicated radiology workstation. WMH will be defined as visually rated hyperintense lesions on T2-weighted axial FLAIR imaging and no corresponding hypo-intensity on T1-weighted axial images. Visual rating scales for WMH will be assigned using the FLAIR images (Fazekas and Scheltens scale) on all scans. The Rotterdam progression score and Schmidt’s progression score will be calculated by side to side review of the baseline and two year scan. Data will recorded on standardised recording sheet and entered into the eCRF held by the Robertson Centre for Biostatistics. |
|  |
|  |
|  |
| **CAROTID MRI UPLOAD AND DISTRIBUTION** |
|  |
|  |
| These images will be a part of the brain MRI protocol at selected centres so will be handled using the processes above. |
|  |
|  |
|  |
| **CAROTID MRI IMAGE ANALYSIS** |
|  |
|  |
| A detailed image analysis protocol will be agreed during the trial start-up phase and will be detailed in a separate document. Image analysis will be performed from a dedicated radiology workstation. |
|  |
|  |
|  |
| **CARDIAC MRI UPLOAD AND DISTRIBUTION** |
|  |
|  |
| This will follow the similar procedures for the brain MRIs but images will be reviewed and co-ordinated by the University of Dundee (GH / AS). |
|  |

|  |
| --- |
| **CARDIAC MRI IMAGE ANALYSIS** |
|  |
|  |
| A detailed image analysis protocol will be agreed during the trial start-up phase and will be detailed in a separate document. Image analysis will be performed from a dedicated radiology workstation.  Image analysis to establish LVM and hence LVMI will be performed using Argus software (VA60C) on a Leonardo Workstation. Endocardial and epicardial borders will be defined independently on all images corresponding to end-diastole (ED) and end-systole (ES) by an MRI physicist segmenter (blind as to the subjects’ treatment arm allocation), with assistance from a consultant radiologist as required. Segmentation rules relating to the appropriate identification of ED and ES phases, along with inclusion or exclusion of appropriate basal slices and papillary muscles will be standardised before the analysis task in order to ensure as much consistency as possible. A single segmenter will be responsible for undertaking the work, since the use of multiple segmenters is known to lead to greater inter-observer variability. The segmentation process will be performed twice for each patient in order to derive a mean value for LVM and consequently improve the precision of the measurement. Our coefficient of reproducibility for LV mass is 7.3%. Pulse wave velocity analysis will be performed using Segment (Version 1.9 R1917, Medviso, Sweden). Aortic region-of-interest contours will be defined at each temporal phase on all datasets to provide flow waveforms for each location. The distance (x) of each measurement plane from the baseline ascending aorta location will be calculated using calipers and an ECG-gated CINE segmented fast low-angle shot (FLASH) dataset that will be acquired in the sagittal oblique ‘candy cane’ orientation. PWV can then be calculated for each patient and volunteer by plotting distance (x) of each location versus transit-time (y). |
|  |
|  |
|  |
| **ABPM INTERPRETATION** |
|  |
|  |
| ABPM data will be automatically downloaded from the device to computer and uploaded to the eCRF. To be considered evaluable, the ABPM report must contain at least 20 hours data, including at least one valid reading for every hour during daytime and every 2 hours during night time.  Mean day-time systolic BP, mean daytime diastolic BP, mean 24-hour systolic blood pressure, mean 24-hour diastolic blood pressure and mean overnight systolic BP, mean overnight diastolic BP will be calculated. |
|  |

|  | | | |
| --- | --- | --- | --- |
| **SAFETY BLOOD TESTING / VENEPUNCTURE** | | | |
|  | | | |
|  | | | |
| Venepuncture will be performed from the antecubital fossa where possible (using a ~ 19G (green needle) vacutainer (or similar) system). A lavender top EDTA tube (or similar) for whole blood haematology testing, a gold top clot activator (or similar) for serum chemistry measures and a grey tube (or similar) for glucose determination will be collected (ca 8 mL in total) when required (TABLE 2).  At run-in phase (week 0), the most recent gamma-GT, cholesterol, triglyceride and glucose levels should be recorded, but specific testing for these is not required unless the tests were conducted more than 4 weeks prior to the visit. | | | |
|  | | | |
|  | | | |
|  | | | |
| **PROCEDURES FOR SAMPLE COLLECTION AND HANDLING FOR BLOOD TESTS** | | | |
|  | | | |
|  |  |  |  |
|  | **BLOOD VOLUME (mL)** | **COLLECTION TUBE** | **SAMPLE DESTINATION** |
|  |  |  |  |
|  |  |  |  |
| **FBC** | 3 | EDTA | Straight to NHS Lab |
|  |  |  |  |
|  |  |  |  |
| **U+E / LFT** | 3 | Gold clot activator | Straight to NHS Lab |
|  |  |  |  |
|  |  |  |  |
| **URATE** | 3 | Gold clot activator | Send to co-ordinating centre |
|  |  |  |  |
|  |  |  |  |
| **GLUCOSE** | 2 | Grey | Straight to NHS Lab |
|  |  |  |  |
|  | | | |
|  | | | |
| Blood for serum uric acid levels will be obtained during the run-in phase (week 4) and at week 104 during the treatment phase. There is potential for change in serum uric acid level to unmask treatment allocation as this is not anticipated to fall on placebo. In this patient group, assessment of serum uric acid level will not be of clinical use so should not be performed during the study. The blood for serum uric acid levels will be measured centrally by the NHS GGC biochemistry laboratory. Details about sampling handling for the serum uric acid are given in APPENDIX 1 and XILO-FIST Sample Handling Manual.  All safety blood results should be reviewed within 3 days of being taken by a member of the study team. All abnormal results will be reviewed by a local investigator if abnormal. | | | |
|  | | | |
|  | | | |
|  | | | |
| **BIOBANKING** | | | |
|  | | | |
|  | | | |
| Where possible, urine and blood samples will be obtained at baseline and at week 104 as outlined in the APPENDIX 1 and XILO-FIST Sample Handling Manual. They will then be couriered to the co-ordinating centre and will be stored in line with NHS Greater Glasgow and Clyde policies. This is encouraged but is not mandatory for trial participation and participants will give specific consent for this. | | | |
|  | | | |

|  | |
| --- | --- |
| **NIHSS AND MRS CERTIFICATION PROCEDURE** | |
|  | |
|  | |
| All NIHSS and mRS raters will require to be certified using available and validated on-line materials. The scoring guidance for these scales will be given to investigators in accompanying documentation. Certificates will be retained in the sponsor site file. | |
|  | |
|  | |
|  | |
| **THE MODIFIED RANKIN SCALE** | |
|  | |
|  |  |
| **DESCRIPTION** | **SCORE** |
|  |  |
|  |  |
| No symptoms at all | 0 |
|  |  |
|  |  |
| No significant disability despite symptoms; able to carry out all usual duties and activities | 1 |
|  |  |
|  |  |
| Slight disability; unable to carry out all previous activities, but able to look after own affairs without assistance | 2 |
|  |  |
|  |  |
| Moderate disability; requiring some help, but able to walk without assistance | 3 |
|  |  |
|  |  |
| Moderately severe disability; unable to walk without assistance and unable to attend to own bodily needs without assistance | 4 |
|  |  |
|  |  |
| Severe disability; bedridden, incontinent and requiring constant nursing care and attention | 5 |
|  |  |

|  |
| --- |
| 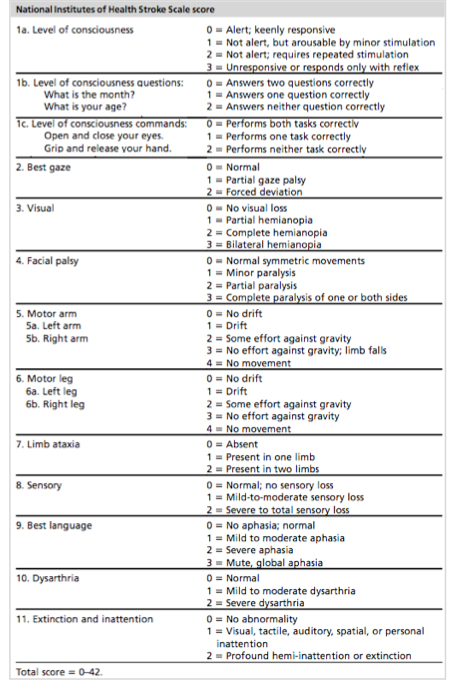 |
|  |

|  |
| --- |
| **COGNITIVE TESTING** |
|  |
|  |
| At baseline screening, dementia will be an exclusion criterion to participation in the study. The assessment of pre-stroke dementia will comprise case-note review for any documented diagnosis of dementia and informant assessment using the 16 item IQCODE. A score of 3.6 or greater will be used as threshold to define probable pre-stroke dementia, this cut-point chosen to maximise specificity. Where initial assessment suggests possible dementia, this information will be shared with the treating clinical team and the patients’ General Practitioner (G.P).  A comprehensive cognitive examination, suitable for clinical trial use and allowing assessment of all relevant cognitive modalities with a focus on “executive function” will be used (based on the ‘Hachinski 30 minute battery’^^[[36]](#endnote-36)^^).This will be performed at baseline and at the two year follow up. The battery comprises: Montreal Cognitive Assessment (MoCA); Animal Naming test of semantic fluency; Controlled Oral Word Association Test; Letter Digit Coding Test; Hopkins Verbal Learning Test; Centre for Epidemiological Studies – Depression Scale (CES-D); Neuropsychiatric Inventory Questionnaire Version (NPI-Q, final follow up visit only) and a trail making test. For the two year assessment IQCODE will be retested and the case-sheet will be re-reviewed to ensure a de-novo diagnosis of dementia has not been made since study recruitment. The MoCA will also be performed at 12 months.  The battery will be administered by a trained assessor and scored to pre-specified marking sheets. Participants will be free to take breaks as needed. If participants are unable to complete the full battery, the assessor will prioritise the MoCA and CES-D. Failure to complete the full battery is expected in some cases and will not be classed as a protocol violation.  The battery contains a screening test for depression (CES-D) and for other behavioural / neuropsychiatric problems (NPI-Q). While these are not diagnostic tests, if patient scores are suggestive of a clinical problem at baseline this information will be shared with the participant and the G.P. Further, the test battery is not diagnostic of dementia. However, substantial cognitive impairment with corresponding functional problems is likely to be indicative of a dementia. All outcomes data will reviewed by the study team and a synthesis of the cognitive data and corresponding functional outcomes (modified Rankin Scale; extended activities of daily living) will be created and shared with the participants’ clinical team and general practitioner if further action is needed.  Details of each of these scales are given in a detailed instruction booklet, which includes instructions for administering each assessment. |
|  |
|  |
|  |
| **QUALITY OF LIFE ASSESSMENT AND QUESTIONNAIRES** |
|  |
|  |
| The EQ-5D and the Stroke Impact Scale Short Form will be measured at baseline and at the end of the study.  During the study, participants will be sent a food frequency questionnaire (FFQ) to complete which assess salt intake. This questionnaire is a semi-quantitative 170 item form where participants will be asked how often they eat certain foods over a 3 month period. The questionnaire is given to participants at the recruitment visit (week 0 treatment phase), it is estimated that the questionnaire will take participants 5-10 minutes to complete. Existing participants will have a questionnaire posted to them with detailed instructions on how to complete. If any of the answers are unclear of missing a member of the research team will contact them via telephone to clarify response.  This will be posted back to Project Management Unit, West Glasgow Ambulatory Care Hospital, Dalnair Street, Glasgow G3 8SW.  The Food Frequency Questionnaire will be analysed by The Department of Human Nutrition at Aberdeen University. |
|  |

|  |
| --- |
| **INVESTIGATIONAL DRUG INFORMATION AND PROCUREMENT** |
|  |
|  |
|  |
| **ALLOPURINOL (300 mg TWICE DAILY)** |
|  |
|  |
| Allopurinol is a xanthine oxidase inhibitor which has been used for many years in the prophylaxis of gout. It is generally well tolerated, and side effects are infrequent. Full details are contained in the SmPC on the XILO-FIST web portal and on the trial master file. A rash develops in approximately 2 percent of patients treated with allopurinol (which participants will be advised to report).It usually subsides after the allopurinol has been discontinued. Gastro-intestinal upset may occur but is uncommon. More serious side effects, such as generalised hypersensitivity, occur in less than1 in 1000 cases and include exfoliative dermatitis, often with vasculitis, fever, liver dysfunction, eosinophilia, and acute interstitial nephritis and Stevens Johnson Syndrome^34^.The rate of adverse reaction is highest in patients with renal dysfunction and in the early stages of treatment and rashes are more common with concurrent amoxicillin therapy^^[[37]](#endnote-37)^^.There is a known interaction with azathioprine and 6-mercaptopurine therapy, other cytotoxic therapies, cyclosporine and didanosine. There are some reports of reductions in formed blood cell counts, although this is very rare (see exclusion criteria (section 3.3) and procedures for safety monitoring). |
|  |
|  |
|  |
| **RATIONALE FOR CHOSEN DOSE** |
|  |
|  |
| We have shown a sustained reduction in UA concentration after stroke with use of allopurinol 300 mg per day (of approximately 0.12 mmol/L across trials)^24,25^, without encountering SAEs attributable to therapy. We have shown this dose to improve cerebral nitric oxide bioavailability in patients with type 2 diabetes^27^ and to have an effect on levels of circulating markers of inflammation in participants with recent stroke^10^. However, a 300 mg twice daily dose of allopurinol may be more effective than 300 mg once daily^26^ and this dose causes regression of LVH in patients with diabetes, renal impairment^28^ and angina and this reduces myocardial ischaemia in patients with angina^29^. Further, other studies have suggested those with the highest falls in serum UA levels will derive most benefit from allopurinol use^^[[38]](#endnote-38)^^. We have therefore chosen to study a dose of allopurinol 300 mg twice daily after an initial one month treatment period of 300 mg once daily. We will allow a lower dose to be used in those who suffer side effects on the higher dose but tolerate the lower dose (this does not include rash where treatment will always be stopped) and in those who have impaired renal function (eGFR between 30 and 60 ml/min). This will be decided by an investigator at a dispensing visit. Steps taken to minimise adverse effects are outlined above. |
|  |
|  |
|  |
| **STUDY INTERVENTION** |
|  |
|  |
| Participants will be randomised in a blinded fashion and on a one to one basis to receive either allopurinol 300 mg or placebo twice daily for two years. During the first month, a 300 mg daily dose of allopurinol or placebo will be taken. All participants will then undergo a dose titration to allopurinol 300 mg twice daily or placebo unless creatinine clearance is < 60 ml / minute (based on eGFR and where the dose will be maintained at allopurinol 300 mg daily or placebo). Dose modification (a reduction from 300 mg twice daily to 300 mg once daily will occur if renal function declines (to a creatinine clearance of < 50 mL/min) or in the event of side effects. After the week 104 visit, treatment with study medication will stop. Where dose has been reduced, it will not be re-escalated.  From March 2020 the week 104 visit can be performed 3 months earlier than week 104 and up-to 6 months later if required due to restrictions required to combat the Covid-19 pandemic. If the week 104 visit is expected to be delayed by more than 2 weeks, the participant will be dispensed 2-months more IMP if it is deemed safe to do so by the site PI. This can be done a maximum of 3 times to give a maximum of 6-months additional IMP. IMP will cease at this point even if face to face contact is not allowed. |
|  |

|  |
| --- |
| **PATIENT ADVICE** |
|  |
|  |
| Participants will be asked to advise the study team of any changes in therapy at the study visits. Participants will be advised to avoid amoxicillin / co-amoxiclav treatment if possible and their G.P will be advised similarly. Should amoxicillin/ co-amoxiclav be required for clinical reasons then participants should withhold study medication during the course. They will also be advised to take their allopurinol with or after food with a full glass of water.  Participants will be advised to cease medication immediately and contact the study team (their local investigator) in the event of a rash. Participants who suffer rash will not be re-challenged but this can be attempted with other more minor side effects.  All participants will be issued with a study card with emergency unblinding information and contact details for the local study team. |
|  |
|  |
|  |
| **FORMULATION AND SOURCE OF DRUG** |
|  |
|  |
| The study drug (allopurinol 300 mg or placebo for oral administration) will be manufactured in accordance with Good Manufacturing Practice by Tayside Pharmaceuticals.  The study drug will be packed as uniquely numbered kits as described in the dispensing guide. All labelling and packaging will be prepared to meet the local regulatory requirements. Each kit will be labelled at a minimum with the following information : name of drug/Placebo, dose instructions, Investigator, EudraCT number, batch number, expiry date, storage instructions, and the statements ‘for clinical trial use only’ and ‘keep out of reach and sight of children’. Study drug will be provided free-of-charge to participating centres. Further information is provided in the study specific IMP Management and Accountability Manual. |
|  |
|  |
|  |
| **STORAGE AND STABILITY** |
|  |
|  |
| All study drugs must be stored in a secure location and will be dispensed by pharmacy who will be delegated this task by the investigator. The study drug should be stored at room temperature below 25^o^C. |
|  |
|  |
|  |
| **DRUG PROCUREMENT** |
|  |
|  |
|  |
| **DRUG ORDERING** |
|  |
|  |
| Drug will only be released to sites once all the appropriate regulatory and governance approvals are in place. Patients will be dispensed study drug at the start of the treatment phase (Treatment phase Week 0), at week 4, week 13, week 26, week 52 and week 78. Additional dispensing can take place for a maximum of 6 months if the week 104 visit is delayed due to restrictions required to combat the Covid-19 pandemic. |
|  |

|  |
| --- |
| **DRUG ACCOUNTABILITY** |
|  |
|  |
| A record of all study drug movements must be kept for accountability purposes. When study drug is received by the pharmacy, they will check for accurate delivery and acknowledge receipt. The dispensing of the study drug to subjects must be recorded on appropriate drug accountability forms.    They should include dates, quantities, batch numbers, expiry dates and any unique code numbers assigned to the investigational product(s) and/or study subjects. Pharmacy should maintain records which document adequately that:   - the patients were provided the doses specified by the protocol/amendment(s) - all study drug provided was fully reconciled.   Only those supplies intended for use in the study should be dispensed to study participants. Unused study drug must not be discarded or used for any purpose other than the present study. Further information is provided in the study specific IMP Management and Accountability Manual. |
|  |
|  |
|  |
| **DESTRUCTION OF UNUSED DRUG** |
|  |
|  |
| Study subjects should be instructed to return any remaining medication at each study visit. Returned medication must be weighed and the result entered in the eCRF. Study specific arrangements are in place for the destruction of investigational medicinal product and this must be documented. These inventories must be made available for inspection by the study sponsor or their designee and regulatory agency inspectors. The local pharmacy will be responsible for the accountability of used and unused trial supplies. Further information is provided in the study specific IMP Management and Accountability Manual. |
|  |
|  |
|  |
| **EMERGENCY UNBLINDING OF TREATMENT ALLOCATION** |
|  |
|  |
| A subject’s investigational product assignment can be unblinded when knowledge of the investigational product assignment is essential for the care of the subject. Unblinding will be via an Interactive Voice Response System (IVRS) supported by the Robertson Centre for Biostatistics and will be available at all times).Unblinding can be performed by medical personnel with access to the appropriate information listed on the patient’s information card or trial master file. Wherever possible, an attempt should be made to contact the CI in advance of emergency unblinding but in the event that this is not possible emergency unblinding should proceed. |
|  |

|  |
| --- |
| **CONCOMITANT MEDICINES** |
|  |
|  |
| Participants taking the medications listed in the exclusion criteria are ineligible for the study. Should an indication for these drugs develop during the study they will be withdrawn from study treatment. Participants taking warfarin will likely attend for extra INR monitoring during the first month of the study. This will be decided by the local investigator in accordance with clinical need, in part based on the participant’s previous INR control. Any change required to the warfarin dose will be documented in the patient’s Oral Anticoagulant Handbook. Participants will continue to attend their anticoagulant service as normal and any additional monitoring can be overseen by them or the local investigator.  Participants who require a course of amoxicillin during the treatment phase will be advised to stop taking study medication for the duration of the amoxicillin. The study drug can then be restarted the following day. |
|  |
|  |
|  |
| **DISPENSING SCHEDULE** |
|  |
|  |
| This will be detailed in the study IMP Management and Accountability Manual. |
|  |
|  |
|  |
| **PROCEDURES FOR SAFETY MONITORING DURING THE TRIAL** |
|  |
|  |
| Participants will have a clinical assessment and blood testing (for full blood count and urea and electrolytes) during the trial. They will be provided with a 24 hour number through which they can access a member of the study team. Participants will be instructed to stop taking the tablets immediately and contact the local study team should they develop rash or fever or any other symptoms which concern them. Their G.P will be informed of their participation in the study and criteria for withdrawal. A trial IDMC will be convened. |
|  |
|  |
|  |
| **CRITERIA FOR WITHDRAWAL OF PARTICIPANTS ON SAFETY GROUNDS AND WITHDRAWAL PROCESS** |
|  |
|  |
| Participants developing a rash, fever, liver dysfunction (defined as bilirubin or transaminase levels increasing to three times the ULN), renal dysfunction (defined as a drop in eGFR to below 30 mL/min), eosinophilia (defined as an eosinophil count of > 0.45*10^9 or a fall in haemoglobin below 10g/dl or neutrophil count of <1.5*10^9 on any blood sample of a platelet count of <50*10^9 that is not due to clumping will cease taking study medication immediately and will be withdrawn from treatment if no alternative cause is found. Where any of these criteria for withdrawal from treatment are met, participants will be assessed by their local study facility and the CI should be informed within 48 hours. They will be referred to the appropriate unit should any treatment be required and otherwise will be followed up by the research team until deemed safe to discharge them. We will not recruit additional participants to replace those who are withdrawn.  Those developing an indication for one of the specified drugs in the exclusion criteria will also cease study medication. Provided they continue to consent, all participants who are withdrawn from treatment will still be followed according to the study protocol  The patient can decide to withdraw from the clinical trial at any time for no reason. The CI or co-investigator also has the rights to withdraw patients from the study if deemed in the best interests of the patient or in the event of AEs, protocol violations, administrative or other reasons. Full details of withdrawal should be recorded on the eCRF. Withdrawn patients should be followed up in accordance with the protocol if they consent to this. If a patient withdraws and withdraws their consent for follow up a withdrawal form will be completed and retained in site files. |
|  |

|  |
| --- |
| **PROCEDURE FOR RESTARTING TREATMENT FOLLOWING TREATMENT INTERRUPTION** |
|  |
|  |
| If treatment is interrupted for any reason, patients will be reviewed in person or by telephone. If treatment interruption is less than 6months in duration and if deemed safe by a study physician, treatment can be recommenced. This should be restarted at the lower dose of 300 mg daily for the first month with dose titration thereafter if tolerated. If treatment interruption is within the first 3 months of the study, then they will be reviewed as planned in the protocol. If it is after 3 months they will be reviewed at one month following re-introduction of treatment for a repeat safety blood check. If the participant is taking warfarin further monitoring may be required. |
|  |
|  |
|  |
| **MAINTENANCE OF TRIAL TREATMENT RANDOMISATION CODES AND PROCEDURES FOR UNBLINING** |
|  |
|  |
| This is a randomised double-blind study. Patients who meet the entry criteria will be assigned to a Patient Study Number (eCRF Number) using the study web portal. Randomisation codes will be generated by an independent statistician based at the Robertson Centre for Biostatistics and will be stored securely by the Robertson Centre for Biostatistics with restricted access. The IVRS system will be available as a back up randomisation system.  Randomisation will utilise a minimisation algorithm which will include presence of WMH at baseline and sub-study eligibility.  Participants will all have a study card with details of contact for the local site investigators. If it is felt necessary by an attending Doctor, he / she can contact the study team. The procedures for unblinding are described above. The randomisation code should only be broken in response to a critical medical situation, if the treating physician considers that knowledge of the investigational product is essential for the clinical management and welfare of the patient. In the event of an SAE, the investigator should, if necessary, discuss the need for emergency unblinding with the CI. The reason for the unblinding will be recorded by the site principal investigator and communicated to the Robertson Centre and the CI. We will assume patients are on active treatment and manage accordingly and only unblind the participant should this be absolutely necessary. The exact circumstances in which consideration of unblinding may be required are impossible to define but may include a hypersensitivity reaction requiring hospital admission, a fall in neutrophil count below 1.5 or significant hepatic or renal impairment. |
|  |

|  |
| --- |
| **PHARMACOVIGILANCE** |
|  |
|  |
|  |
| Predictable side effects of the IMP (allopurinol) used in this trial are referred to in the SmPC. Screening for potential complications will be undertaken as detailed above (safety bloods, clinical reviews). An investigator will evaluate individual adverse events at each contact with the participant and complete a dedicated section in the eCRF where these are defined as recordable. |
|  |
|  |
|  |
| **DEFINITIONS OF ADVERSE EVENTS** |
|  |
|  |
| **Adverse Event (AE)** - Any untoward medical occurrence in a subject to whom a medicinal product has been administered, including occurrences which are not necessarily caused by or related to that product.  **Adverse Reaction (AR)** - Any untoward and unintended response in a subject to an investigational medicinal product which is related to any dose administered to that subject.  **Serious Adverse Event (SAE)** or **Serious Adverse Reaction (SAR) -** Any adverse event or adverse reaction that   1. results in death 2. is life threatening 3. requires hospitalisation or prolongation of existing hospitalisation 4. results in persistent or significant disability or incapacity 5. consists of a congenital anomaly or birth defect. 6. is otherwise considered medically significant by the investigator.   *Important adverse events/ reactions that are not immediately life-threatening or do not result in death or hospitalisation but may jeopardise the subject or may require intervention to prevent one of the other outcomes listed in the definition above such as clinically significant laboratory values, cancer and overdose.*  **Suspected Serious Adverse Reaction (SSAR)** - Any adverse reaction that is classed in nature as serious and which is consistent with the information about the medicinal product in question set out in the SmPC.  **Suspected Unexpected Serious Adverse Reaction (SUSAR)** - Any adverse reaction that is classed in nature as serious and which is **not** consistent with the information about the medicinal product in question set out in the SmPC. |
|  |

|  | | | |
| --- | --- | --- | --- |
| **RECORDING AND REPORTING AEs/SAEs** | | | |
|  | | | |
|  | | | |
| AEs must be recorded, notified, assessed, reported, analysed and managed in accordance with the Medicines for Human Use (Clinical Trials) Regulations 2004 (as amended) and the study protocol. Allopurinol is a commonly used and licensed drug with an established toxicity profile. Adverse reactions are rare in the overall treated population, especially if hepatic disorder and renal dysfunction are not present. When adverse reactions arise, they typically occur early in the course of treatment or after a dose increase. | | | |
|  | | | |
|  | | | |
| **RECORDING** | | | |
|  | | | |
|  | | | |
| AEs and SAEs occurring before randomisation will not be recorded or reported.  All SAEs occurring within the first 13 weeks of the treatment phase will be recorded in the eCRF and reported to sponsor in order to capture all potential allopurinol related adverse reactions during the main risk period. During this period, events of special interest will also be recorded, even if they do not meet criteria for an SAE. Thereafter, and up to 30 days after completing the study, all SAEs and events of special interest will be recorded in the eCRF but only SAEs not expected to occur in stroke patients, SUSARs, and events of special interest that meet criteria for an SAE will be reported to the sponsor. Events of special interest include trial outcomes (cardiovascular events) and the established common or significant adverse reactions following allopurinol use (detailed in the SmPC) that would not be expected to occur in a population of stroke patients. Expected SAEs in stroke patients are listed in **Error! Reference source not found.**^^[[39]](#endnote-39)^^ and events of special interest are listed in **Error! Reference source not found.**^39^.  Participants will be asked at each study visit about the occurrence of SAEs and events of special interest since the last visit.  Where an event requires recording, full detail (including the nature of the event, start and stop dates, severity, relationship to study drug and outcome) will be recorded in the subject’s medical records and in the eCRF. These events will be monitored and followed up until satisfactory resolution or stabilisation.  All AEs must be assessed for seriousness. SAEs must be assessed for causality, expectedness and severity. | | | |
|  | | | |
| **Causality -** This should be assessed by the CI or PI and should be described using the following categories: | | | |
|  |  | |  |
|  | - Unrelated to study drug - Possibly related to study drug - Probably related to study drug - Definitely related to study drug | |  |
|  |  |  |  |
|  |  |  |  |
|  |  |  |  |
|  |  |  |  |
|  |  |  |  |
|  |  |  |  |
|  |  | |  |
|  | | | |
| **Severity -** This should be assessed by the CI or PI and should be described using the following categories: | | | |
|  |  | |  |
|  | Mild: | awareness of event but easily tolerated |  |
|  |  | |  |
|  |  | |  |
|  | Moderate: | discomfort enough to cause some interference with usual activity |  |
|  |  | |  |
|  |  | |  |
|  | Severe: | inability to carry out usual activity |  |
|  |  | |  |
|  | | | |
|  | | | |
| **Assessment of expectedness –** The expectedness of an adverse reaction is assessed against the Reference Safety Information (RSI) (i.e. the list of expected reactions detailed in the approved SmPC for the Investigational Medicinal Product). This will be assigned by the AE and Outcome Review Committee. | | | |

|  |
| --- |
| **REPORTING** |
|  |
|  |
| Details of SAEs and events of special interest (as detailed in 5.2.1) arising during the clinical trial will be entered into the eCRF as soon as reasonably practicable. The site must enter details to the eCRF within 24 hours of first becoming aware of the event. Any follow up information should also be reported.  Once details are entered into the eCRF, SAEs will be reviewed within 24 hours by the CI or delegate who will decide if it is reportable to the sponsor. If the event is reportable to sponsor this will be done within 24 hours of the CI or delegate becoming aware of the event.  SAEs recorded in the eCRF and reported to the sponsor will be transferred to the Glasgow Pharmacovigilance database.  If reporting via the eCRF is not possible, a paper SAE form can be downloaded from the Glasgow Clinical Trials Unit website: http//www.glasgowctu.org/complete-paper-sae.apsx. This should be completed and faxed to the PV office (Fax No: +44 (0) 141 357 5588).  Events of special interest will be reviewed by the Clinical Endpoint Committee according to an agreed charter. The Clinical Endpoint Committee can upgrade an AE to an SAE. If an event is upgraded to an SAE, this will be reported to the sponsor within 24 hours of the Clinical Endpoint Committee becoming aware of the event.  **SUSARs** – All SUSARs must be reported an expedited fashion to the Medicines and Healthcare Regulatory Authority (MHRA) and Research Ethics Committee (REC)   - **Fatal or life threatening SUSARs**   Not later than 7 days after the sponsor had information that the case fulfilled the criteria for a fatal or life threatening SUSAR, and any follow up information within a further 8 days.   - **All other SUSARs**   Not later than 15 days after the sponsor had information that the case fulfilled the criteria for a SUSAR.  The Glasgow Clinical Trials Unit PV Office will report SUSARs on behalf of the CI to the MHRA via the eSUSAR reporting system and to the REC by email The PV Office will forward a copy of any SUSAR reports with an accompanying cover letter to the CI for onward distribution to co-investigators.  **Pregnancy** - Pregnancy is not considered an AE or SAE. However, the PIs will report pregnancy information on any female subject or male partner of a female subject who becomes pregnant while participating in the Trial to the sponsor within two weeks of first becoming aware of the pregnancy. This report should be provided to the PV office on the Pregnancy Notification Form provided by the sponsor (on www.glasgowctu.org). The subject will also be followed to determine the outcome of the pregnancy. Information on the status of the mother and child will be forwarded by the PI to the sponsor. |
|  |
|  |
|  |
|  |
| **UNBLINDING FOR SUSAR REPORTING** |
|  |
|  |
| In the event of a potential SUSAR, the sponsor (but not the investigators) will be unblinded to facilitate appropriate reporting to the MHRA and REC. |
|  |
|  |
|  |
| **ANNUAL SAFETY REPORTS** |
|  |
|  |
| An annual safety report must be submitted to MHRA and REC as soon as is practicable (and within 60 days) of the anniversary of the issue of the Clinical Trials Authorisation (CTA). This will be in the Development Safety Update Report (DSUR) format. The DSUR will be prepared and submitted by the PV Office in liaison with the CI. |
|  |

|  |
| --- |
| **STATISTICS AND DATA ANALYSIS** |
|  |
|  |
|  |
| **PRIMARY ENDPOINT** |
|  |
|  |
| The study is primary endpoint is WMH progression rate over 2 years, defined using the Rotterdam Progression Score. |
|  |
|  |
|  |
| **SECONDARY ENDPOINT(S)** |
|  |
|  |
| Secondary endpoints are:   - Change in mean day-time systolic BP at 1 month - Change in mean day-time diastolic BP at 1 month - Schmidt’s Progression Score - WMH volume at 2 years - New brain infarction on MRI - Rotterdam Progression Score with those who die / become too frail to undergo MRI being assigned the highest score - MoCA score - Change in mean day-time systolic BP at 2 years - Change in mean day-time diastolic BP at 2 years   The following outcomes re exploratory   - Fazekas score - Scheltens scale score - Blood pressure variability - One month mean day-time diastolic blood pressure - Two year mean day-time diastolic blood pressure - Clinic blood pressure - Incident atrial fibrillation - Recurrent stroke - Recurrent MI, stroke or cardiac death - Hospitalisation for, or incident heart failure - Incident dementia - Mortality - Animal naming test - Controlled work association test - Hopkins verbal learning test - Trail making test - Quality of life (EQ-5D, SS-QOL) - Modified Rankin scale score |
|  |
|  |
|  |
| **CARDIAC SUB-STUDY PRIMARY ENDPOINT** |
|  |
|  |
| The primary endpoint for this sub-study will be change in measured LVM at 2 years. |
|  |
|  |
|  |
| **CARDIAC SUB-STUDY SECONDARY ENDPOINT(S)** |
|  |
|  |
| Secondary endpoints are:   - Change in ejection fraction - Change in end diastolic volume - Change in end systolic volume - Change in stroke volume - Change in left atrial diameter |
|  |

|  |
| --- |
| **CARotid SUB-ANALYSIS PRIMARY ENDPOINT** |
|  |
|  |
| Change in carotid plaque volume at 2 years will be the primary endpoint for this study. |
|  |
|  |
|  |
| **SUB-ANALYSIS SECONDARY ENDPOINT(S)** |
|  |
|  |
| Plaque morphology measures will be secondary endpoints. |
|  |
|  |
|  |
| **TIME POINTS FOR ENDPOINT EVALUATION** |
|  |
|  |
| Brain MRI will be performed at baseline and 2 years using either 1.5T or 3T MRI. The Rotterdam progression score will be calculated between baseline and subsequent scans. MRI analysis will be performed centrally at the core laboratory (University of Glasgow). This will be performed as soon as possible following the 2 year MRI scan being performed.  All secondary endpoints will be evaluated at 2 years with the exception of clinic blood pressure (which will be assessed at 1 month, 2 years and as a mean on treatment level) and MoCA score which will be assessed at 1 year. |
|  |
|  |
|  |
| **STATISTICAL ANALYSIS PLAN** |
|  |
|  |
| Full details of all statistical issues and planned statistical analyses will be specified in a separate statistical analysis plan (SAP) which will be agreed before the final locking and unblinding of the study database. |
|  |
|  |
|  |
| **GENERAL CONSIDERATIONS** |
|  |
|  |
| Analyses will be carried out according to the intention-to-treat (ITT) principle, in that subjects will be analysed according to their randomised group, regardless of the treatment actually received. Primary and main secondary analyses will be repeated in a per-protocol (PP) population of subjects who have no major protocol deviations. Baseline data will be summarised by treatment group in the ITT population without formal statistical comparison. |
|  |

|  |
| --- |
| **EFFICACY ANALYSES** |
|  |
|  |
| Efficacy data will be summarised by treatment group in the ITT and PP populations. Unadjusted comparisons of continuous measures will be made using t-test or Wilcoxon-Mann-Whitney tests as appropriate; binary endpoints will be compared using Fisher’s Exact Test. Adjusted analyses will use regression models, adjusted for variables used in the minimisation algorithm. Distributional assumptions will be assessed visually, and where necessary, the outcome will be transformed, or a generalized linear model will be used with appropriate link and variance function. Treatment effect estimates will be reported with 95% confidence intervals and p-values. |
|  |
|  |
|  |
| **PRIMARY ENDPOINT** |
|  |
|  |
| WMH progression is not expected to follow a Normal distribution. Unadjusted comparison of treatment groups will use a Wilcoxon-Mann-Whitney test. A generalized linear regression model, with appropriate link and variance function, will be used to model WMH progression in relation to treatment and variables used in the minimisation algorithm. A further model will be fitted adjusting for other baseline characteristics found to be associated with WMH progression during blinded analysis.  This model will be extended in exploratory sub-group analysis to assess the mediating effects of other study outcomes, such as changes in BP and uric acid post-randomisation. |
|  |
|  |
|  |
| **SECONDARY ENDPOINT** |
|  |
|  |
| Secondary efficacy outcome measures will be analysed using appropriate two-sample tests followed by regression analyses to estimate between-group differences adjusted for variables used in the minimisation algorithm. For measures recorded at baseline as well as at follow-up, regression models will be adjusted for the baseline value. For endpoints measured at several time points, each time point will first be analysed separately, then a repeated measures model will be applied to model measurements at all time points simultaneously. |
|  |
|  |
|  |
| **PLANNED SUB-GROUP ANALYSIS** |
|  |
|  |
| The moderating effects of baseline uric acid level and fall in serum UA level in the primary analysis will be assessed through use of interaction terms in the model. These methods will also be used to investigate the moderating effects of other baseline characteristics in an exploratory manner. |
|  |
|  |
|  |
| **SAFETY ANALYSES** |
|  |
|  |
| Safety data will be reported in the safety population, defined as those who received at least one dose of study medication. Data will be summarised by treatment group without formal statistical comparison. Serious adverse events will be reported by treatment group according to body system and preferred term as classified in MedDRA. The number of events and the number and percentage of patients suffering at least one event in each category will be reported. Similar summaries will be provided for the subgroup of events that are at least possibly related to study medication, are unexpected, or both (i.e. SUSARs). Summaries will also be provided for events that start during periods defined by the study visits. |
|  |

|  |
| --- |
| **SOFTWARE FOR STATISTICAL ANALYSIS** |
|  |
|  |
| All statistical analysis will be performed using SAS for Windows v9.3 or R for Windows v3.0.0, or later versions of these programs. |
|  |
|  |
|  |
| **SAMPLE SIZE** |
|  |
|  |
| We assume that up to 90% of participants will have evidence of WMH at baseline and that, over two years, approximately 64% will progress by one point or more on the RPS (based on 2/3 of the 3 year progression rate^10^ (WHM progression has previously been described to be linear)) and the mean progression score in the placebo group will be 1.293. This and the progression rate seen with a 30% reduction in WMH progression score is shown in table 5. A sample size of 192 participants per group would give 80% power to detect this difference at a 5% significance level. This treatment effect is substantially less than the 80% relative reduction seen in the PROGRESS MRI study^12^ and given allopurinol has been shown to have a similar effect to inhibitors of the rennin angiotensin system on LVH in other disease states; we believe this is a conservative and appropriate basis for our sample size calculation.  Although we will minimise loss to clinical follow up (completed for all except one participant in our pilot study) we have increased sample size to account for a 10% drop out rate seen in our pilot study and by an additional small amount to account for the fact that those with no WMH at baseline may progress at lower rate. We will thus randomise 232 participants per group (10% drop outs will give data on 209 participants giving a further 17 participants (8%) per group to ensure sufficient power). For the ABPM analyses, inclusion of 101 participants per group will give 80% power at the 0.05 significance level to verify the 3.3 mmHg reduction in systolic BP seen in the recent meta-analysis^32^ (assumed SD of change in SBP 8.3). We will therefore have sufficient power to assess presence of clinically important change in blood pressure.  For the cardiac sub study, inclusion of 45 participants per group will give 80% power to verify the 1.8 g/m^2^ difference in the primary endpoint of LV mass index seen in a recent trial of patients with IHD (assumed SD 3) so will recruit 50 per group to allow for drop outs. We predict 40 to 50% of participants will have LVH at baseline. From our in-house validation of MRI aortic pulse wave velocity (PWV), typical mean PWV in older population volunteer with higher risk of cardiovascular disease for n=10, mean age: 52± 7 y, PWV: 8.00 m/s ± 1.40. Associated risk factors for stroke overlap with other diseases such as diabetes and peripheral arterial disease (PAD). The typical PWV in patients with PAD for n=7, mean age: 65± 11 y, PWV: 9.4 m/s ± 4.1. Assuming study power of 80%, p=0.05, a difference in population means of 2.7 m/s and a standard deviation of +/- 2.0 m/s, a total of *n=33* subjects in each cohort would be required to demonstrate PWV differences as a secondary endpoint to the cardiac MR sub study.  Few data are available to support a power calculation for the carotid sub analysis. However, previous studies have shown change in PV following statin therapy^^[[40]](#endnote-40)^^. For example, PV fell by 5.8% following statin treatment from a mean (SD) at baseline of 1036 (300) mm^3^ to 976 (331) mm^3^ at 6 months^40^. Assuming the SD of PV at 2 years is no more than 400 mm^3^, then a study with 40 participants per group will have 80% power at the 5% significance level to detect a difference of 250 mm^3^, based on a two-sample t-test. The difference detectable is reduced, however, if we apply analysis of covariance (linear regression with adjustment for baseline); for example, if the correlation between baseline and 2 years is 0.6, a difference of approximately 200 mm^3^ could be detected with 80% power. We will therefore enrol at least 90 participants to this sub-study (assuming a 10% dropout rate).  The sample size calculation for WMH progression was made using nQuery Advisor® v7.0, based on a Wilcoxon-Mann-Whitney test. Other sample size calculations were based on two-sample t-tests. All sample size calculations were based on 80% power and a two-sided 5% significance level. We will apply more sophisticated regression methods to each outcome, to maximise power, and to investigate mediating and moderating effects. |
|  |

|  | | |
| --- | --- | --- |
| **% WITH 30% REDUCTION IN PROGRESSION RATE** | | |
|  | | |
|  |  |  |
| **RPS SCORE** | **UNTREATED %** | **TREATED %** |
|  |  |  |
|  |  |  |
| 0 | 36.3 | 46.5 |
|  |  |  |
|  |  |  |
| 1 | 27.1 | 29.2 |
|  |  |  |
|  |  |  |
| 2 | 18.6 | 15.3 |
|  |  |  |
|  |  |  |
| 3 | 10.2 | 6.3 |
|  |  |  |
|  |  |  |
| 4 | 5.0 | 2.1 |
|  |  |  |
|  |  |  |
| 5 | 2.1 | 0.5 |
|  |  |  |
|  |  |  |
| 6 | 0.6 | 0.1 |
|  |  |  |
|  |  |  |
| 7 | 0.1 | 0 |
|  |  |  |
|  |  |  |
| 8 | 0 | 0 |
|  |  |  |
|  |  |  |
| **MEAN SCORE** | 1.293 | 0.905 |
|  |  |  |
|  | | |
|  | | |
| **LEVEL OF SIGNIFICANCE TO BE USED** | | |
|  | | |
|  | | |
| The primary analysis will be judged at a 5% significance level. Other analyses will not be adjusted for multiple comparisons. | | |
|  | | |
|  | | |
|  | | |
| **CRITERIA FOR TERMINATION OF TRIAL** | | |
|  | | |
|  | | |
| It is planned that recruitment will conclude once the full sample of 464 patients has been enrolled. However, an independent data safety monitoring committee will be convened and review safety at 6 monthly intervals. Criteria for trial determination will be decided by the IDMC. No interim efficacy analysis is planned. | | |
|  | | |
|  | | |
|  | | |
| **PROCEDURES FOR ACCOUNTING FOR MISSING DATA** | | |
|  | | |
|  | | |
| There will be no imputation of missing data for the primary or secondary endpoints. The level of missing data will be reported for all endpoints. | | |
|  | | |
|  | | |
|  | | |
| **PROCEDURES FOR REPORTING AND DEVIATIONS FROM THE ORIGINAL STATISTICAL PLAN** | | |
|  | | |
|  | | |
| A detailed SAP will be agreed before the final locking and unblinding of the study database. Any deviations from this plan will be documented and justified in the final study report. | | |
|  | | |

|  |
| --- |
| **SELECTION OF PARTICIPANTS TO BE INCLUDED IN THE ANALYSES** |
|  |
|  |
| Efficacy analysis will be conducted on an intention to treat basis but there will be no imputation of missing data (such as that due to participant death or withdrawal for imaging and other endpoints). |
|  |
|  |
| Safety data will be reported and analysed using the safety set of patients who were randomised and received at least one dose of study drug. |
|  |

|  |
| --- |
| **TRIAL CLOSURE / DEFINITION OF END OF TRIAL** |
|  |
|  |
|  |
| For the purposes of regulatory requirements the end of the trial is defined as one month after the date of the last investigational visit for the last patient undergoing protocol treatment. |
|  |

|  | | |
| --- | --- | --- |
| **SOURCE DATA / DOCUMENTS** | | |
|  | | |
|  | | |
|  | | |
| All participant data will be identified by the participant study identification. The format of the source data are outlined in table 6 and all data will be held in the participant’s clinical record. eCRFs will be completed by either the study nurse or an investigator at each study visit.  All study data will be held in the study Robertson Centre for Biostatistics. The Robertson Centre for Biostatistics manages all studies to the highest standards in accordance with its internal Standard Operating Procedures, Principles of Good Clinical Practice, the European Union Clinical Trials Directive 2001/20/EC, the ICH Harmonised Tripartite Guideline: Statistical Principles for Clinical Trials E9 and all other industry legal and regulatory guidelines. It has extensive experience of managing data in the context of privacy and data protection legislature, including the Data Protection Act 1998 and EU Data Protection Directive 95/46/EC. The Centre is certified for ISO 9001:2008 and ISO 27001:2005.  Only the study investigators will have access to participant identifiable data. However, access to participant records will be required for trial-related monitoring, audits and regulatory inspections, which will include direct access to source data and documents. | | |
|  | | |
|  | | |
|  | | |
| **DEFINITION AND HANDLING OF SOURCE DATA**  ***note the study workbook can be scanned into the clinical records** | | |
|  | | |
|  |  |  |
|  | **SOURCE DATA** | **ENTRY OF SOURCE DATA TO ECRF** |
|  |  |  |
|  |  |  |
| **Baseline Demographic Variables** | Entry made into clinical records * | Direct entry into eCRF |
|  |  |  |
|  |  |  |
| **Safety Blood Tests** | Held in patients’ clinical record | Direct entry into eCRF |
|  |  |  |
|  |  |  |
| **Brain MRI** | Clinical report in patients’ clinical record | Study related measures entered into eCRF |
|  |  |  |
|  |  |  |
| **Carotid MRI** | Clinical report in patients’ clinical record | Study related measures will not be entered into the eCRF and will be securely transferred to the RCB |
|  |  |  |
|  |  |  |
| **ECG** | Held in patients’ clinical record | Key parameters entered directly into eCRF |
|  |  |  |
|  |  |  |
| **Echocardiography** | Held in patients’ clinical record | Key parameters entered directly into eCRF |
|  |  |  |
|  |  |  |
| **ABPM** | Held in patients’ clinical record | Key parameters uploaded directly into eCRF, upload of report |
|  |  |  |
|  |  |  |
| **Cardiac MRI** | Held in patients’ clinical record | Study related measures will not be entered into the eCRF and will be securely transferred to the RCB |
|  |  |  |
|  |  |  |
| **Cognitive Tests** | Entry made into clinical records * | Key parameters entered directly into eCRF |
|  |  |  |

|  |
| --- |
| **DATA HANDLING AND RECORD KEEPING** |
|  |
|  |
|  |
| **COMPLETION OF ECRF** |
|  |
|  |
| The eCRF will be developed by the Robertson Centre for Biostatistics. Access to the eCRF will be restricted, via a study-specific web portal, and only authorised site-specific personnel will be able to make entries to their patient data via the web portal.  The Investigator, or his/her designee will be responsible for all entries into the eCRF and will confirm (electronically) that the data are accurate and complete, and that they have reviewed all of the data contained in the eCRF. |
|  |
|  |
|  |
| **DATA VALIDATION** |
|  |
|  |
| Data will be validated at the point of entry into the eCRF. Any additional data discrepancies will be flagged to the investigator and any data changes will be recorded in order to maintain a complete audit trail (reason for change, date change made, who made change). |
|  |
|  |
|  |
| **DATA SECURITY** |
|  |
|  |
| The Robertson Centre for Biostatistics systems are fully validated in accordance with industry and regulatory standards, and incorporate controlled access security. High volume servers are firewall protected and preventative system maintenance policies are in place to ensure no loss of service. Web servers are secured by digital certificates. Data integrity is assured by strictly controlled procedures, including secure data transfer procedures. |
|  |
|  |
|  |
| **DATABASE SOFTWARE** |
|  |
|  |
| Data will be stored in MS SQL Server. |
|  |
|  |
|  |
| **RECORD RETENTION** |
|  |
|  |
| All data and samples from the trial will be retained by the investigators for 20 years after the end of the trial. |
|  |
|  |
|  |
| **ARCHIVING** |
|  |
|  |
| All study documentation will be archived on the conclusion of the study (LPLV) and retained for at least 10 years.  eCRF data will be stored by the Robertson Centre for Biostatistics for 20 years after completion of the trial. |
|  |

|  |
| --- |
| **TRIAL MANAGEMENT** |
|  |
|  |
|  |
| The trial management teams will be in place before recruitment begins. |
|  |
|  |
|  |
| **ROUTINE MANAGEMENT OF TRIAL** |
|  |
|  |
| The trial will be co-ordinated from the Western Infirmary Stroke Unit, Glasgow and will be overseen by the Trial Management Committee (TMC) which comprises the applicants and is chaired by Prof J Dawson. |
|  |
|  |
|  |
| **TRIAL STEERING COMMITTEE (TSC)** |
|  |
|  |
| The TSC will oversee conduct of the trial and include members suggested in NIHR guidelines for GCP. The committee will include Prof Dawson, Professor Lees, Professor Struthers, Dr Forbes, an independent chair (Prof Phillip Bath), one independent member (agreed by Prof Bath) and a lay representative. The TSC will meet at least annually and teleconference when required. |
|  |
|  |
|  |
| **INDEPENDENT DATA MONITORING COMMITTEE (IDMC)** |
|  |
|  |
| The IDMC will be independent and follow MRC guidelines for GCP. The aim is to ensure the safety, rights and well-being of the trial participants in the light of data from the trial and relevant external sources (for example, data from other trials). The IDMC will be chaired by Professor Peter Sandercock. It will also include Professor Gary Ford and one other clinical member as suggested by the chair and approved by the steering committee. A statistician from the Robertson Centre for Biostatistics will also provide support. Full IDMC reviews will occur annually with interim reviews six monthly. |
|  |
|  |
|  |
| **TRIAL WRITING COMMITTEE** |
|  |
|  |
| The writing committee have responsibility for writing all abstracts and manuscripts for publication and will comprise the applicants and the TSC chair. They are responsible for approving content and dissemination, and will be named authors, of all publications, abstracts and presentations arising from the study and for assuring the confidentiality and integrity of the study. It will provide collaborators with approved publicity material and information updates at regular intervals during the course of the study. The definitive publications from the trial will be written with input from the collaborators and will acknowledge all those who have contributed to the study.  In addition a contributing institution may add a name of another clinician as an author on relevant publications if this individual is responsible for > 10% of the total number of evaluable patients entered through that centre.  No site or individual will publish data without prior approval of the writing committee. |
|  |

|  |
| --- |
| **STUDY MONITORING** |
|  |
|  |
|  |
| Study Monitoring Visits will be conducted by NHS Greater Glasgow and Clyde (GG&C) Monitor(s). The level, frequency and priorities of monitoring will be based on the outcome of the completed monitoring risk assessment, and will be clearly documented in the Monitoring Plan which will be approved by the NHS GG&C Research Governance Manager. As standard, Monitoring Visit(s) will cover Site File review, review of Informed Consent Forms (ICFs), Source Data Verification (SDV), Investigational Medicinal Product accountability, and SAE reporting. Each site will be monitored separately and will as a basis receive a Site Compliance Visit prior to the start of recruitment, a full monitoring visit when there are participants active on the trial, and a close out visit after the last subject has completed the last visit. |
|  |

|  |
| --- |
| **PROTOCOL AMENDMENTS** |
|  |
|  |
|  |
| Any change in the study protocol will require an amendment. Any proposed protocol amendments will be initiated by the CI and any required amendment forms will be submitted to REC and/or MHRA. The Sponsor will determine whether an amendment is non-substantial or substantial and will review all amended documents prior to submission to REC and/or MHRA. All amended versions of the protocol will be signed by CI and sponsor representative. Before the amended protocol can be implemented (or sent to other participating sites) favourable opinion/approval must be sought from the original reviewing REC, MHRA and Sponsor. All protocol versions and their amendments must be notified to the study team and to the data centre**.** |
|  |

|  |
| --- |
| **ETHICAL CONSIDERATIONS** |
|  |
|  |
|  |
| **ETHICAL CONDUCT OF STUDY** |
|  |
|  |
| Study will be carried on accordance with the World Medical Association Declaration of Helsinki (1964) and it revisions (Tokyo (1975), Venice (1983), Hong Kong (1989), South Africa (1996) and Edinburgh (2000)).  There are no special ethical considerations pertaining to this study. Favourable ethical opinion will be sought before patients are entered into this clinical trial. Trial patients will only be allowed to enter the study once they have provided written informed consent.  The CI will update the ethics committee of any new information related to the study. |
|  |
|  |
|  |
| **INFORMED CONSENT** |
|  |
|  |
| Written informed consent will be obtained from each trial patient. The research nurse or investigator will explain the exact nature of the study in writing, provision of patient information sheet, and verbally. This will include the known side-effects that may be experienced, and the risks of participating in this clinical trial. Trial patients will be informed that they are free to withdraw their consent from the study or study treatment at any time.  We will supplement our participant information sheets with established resources that explain MRI scanning and the blood pressure monitoring (<http://www.bhf.org.uk/heart-health/tests/mri-scans.aspx>, <http://www.patient.co.uk/pdf/4705.pdf>, <http://www.nhs.uk/Conditions/MRI-scan/Pages/How-does-it-work.aspx> and <http://www.bhsoc.org/resources/abpm/>). |
|  |

|  |
| --- |
| **INSURANCE AND INDEMNITY** |
|  |
|  |
|  |
| XILOFIST is co-sponsored by NHS Greater Glasgow and Clyde and the University of Glasgow. The sponsors will be liable for negligent harm caused by the design of the trial. NHS Indemnity is provided under the Clinical Negligence and Other Risks Indemnity Scheme (CNORIS). As the substantive employer of the CI, The University of Glasgow also has insurance with Royal and Sun Alliance. It will be confirmed prior to the trial starting that insurance cover will be provided automatically under the current policy. The insurance cover will be subject to NHS indemnity being in place and REC approval being obtained.  The NHS has a duty of care to patients treated, whether or not the patient is taking part in a clinical trial, and the NHS remains liable for clinical negligence and other negligent harm to patients under this duty of care.  As this is a clinician-led study there are no arrangements for no-fault compensation. |
|  |

|  |
| --- |
| **FUNDING** |
|  |
|  |
|  |
| The study is funded by a Stroke Association and British Heart Foundation Joint Programme grant. |
|  |

|  |
| --- |
| **CO-SPONSOR RESPONSIBILITIES (NHS GREATER GLASGOW AND CLYDE / UNIVERSITY OF GLASGOW)** |
|  |
|  |
|  |
| Prior to study initiation, a non-commercially funded clinical trial co-sponsorship agreement will be put in place between NHS Greater Glasgow and Clyde and University of Glasgow. The role and liabilities each organisation will take under The Medicines for Human Use (Clinical Trials) Regulations, 2004 SI 2004:1031 are laid out in this agreement signed by both organisations. The University of Glasgow shall be responsible for carrying out the obligations and responsibilities set out in the aforementioned agreement, and shall be deemed "sponsor" for the purposes of, Part 3 of the Regulations in relation to the Study. NHS Greater Glasgow and Clyde shall be responsible for carrying out the obligations and responsibilities set out in the agreement, and shall be deemed "sponsor" for the purposes of, Parts 4, 5, 6 and 7 of the Regulations in relation to the Study. |
|  |

|  |
| --- |
| **ANNUAL REPORTS** |
|  |
|  |
|  |
| A biannual progress report will be submitted to the funder, the first being submitted 6 months from the date that all trial related approvals are in place. Annual reports will be submitted to the ethics committee, regulatory authority and sponsor with the first submitted one year after the date that all trial related approvals are in place. |
|  |

|  |
| --- |
| **DISSEMINATION OF FINDINGS** |
|  |
|  |
|  |
| Study results will be submitted to an International Conference and will be submitted for publication in a peer review journal. The findings will be made open access. A lay summary will be given to those participants who wish to receive it (participants will be asked at their last study visit). |
|  |

|  |
| --- |
| **REFERENCES** |
|  |

|  |
| --- |
|  |
|  |
|  |
|  |
| **SAMPLE COLLECTION FOR BIOBANKING** |
|  |
|  |
|  |
| Non-fasting samples should be taken at baseline and at 104 week visit.  **Required blood tubes -** 1 x SST tube, 2 x 4 mL EDTA (lavender) tube  **Required urine tubes -** 1 x universal or other similar container  **Sample Labelling** All blood and urine samples will be labelled with the participant study ID. The date of sampling and the visit type (baseline / 24 month) should also be annotated.  **Sample Processing** All blood and urine samples should be processed and frozen within two hours of being obtained.  **Urine Samples**  *Aliquoting*  The urine should be aliquoted into the supplied tubes.  *Freezing and Storage*  The urine aliquots should be frozen at either -20°C or -80°C (-80°C preferred).  **Blood Samples**  *Centrifuge of Blood Samples*  The SST tube should be left to stand at room temperature until the sample has clotted (approximately 30 minutes). They should then be centrifuged at 1500 g (3000 rpm) for 15 minutes until blood is well separated.  *Aliquoting*  The serum should be aliquoted into supplied tubes.  *Freezing and Storage*  Samples should be frozen immediately after storage at either -20°C or -80°C (-80°C preferred).  **EDTA (Lavender) Tube**  This should be frozen at -80°C.  Please refer to XILO-FIST Sample Handling Manual for further information. |
|  |

|  | | | | | |
| --- | --- | --- | --- | --- | --- |
|  | | | | | |
|  | | | | | |
|  | | | | | |
|  | | | | | |
| **EXPECTED EVENTS (cont.)** | | | | | |
|  | | | | | |
|  | | | | | |
|  |  |  |  |  |  |
|  | **Serious Adverse Event (AE)** | **Number of Occurrences** | **% of Total SAE Occurrences** | **Cumulative % of SAE Occurrences** |  |
|  |  |  |  |  |  |
|  |  |  |  |  |  |
|  | **Stroke in Evolution** | 350 | 11.240 | 11.2 |  |
|  |  |  |  |  |  |
|  |  |  |  |  |  |
|  | **Pneumonia** | 174 | 5.588 | 16.8 |  |
|  |  |  |  |  |  |
|  |  |  |  |  |  |
|  | **Ischemic Cerebral Infarction** | 151 | 4.849 | 21.7 |  |
|  |  |  |  |  |  |
|  |  |  |  |  |  |
|  | **Brain Edema** | 135 | 4.335 | 26.0 |  |
|  |  |  |  |  |  |
|  |  |  |  |  |  |
|  | **Cardiac Failure** | 106 | 3.404 | 29.4 |  |
|  |  |  |  |  |  |
|  |  |  |  |  |  |
|  | **Myocardial Infarction** | 90 | 2.890 | 32.3 |  |
|  |  |  |  |  |  |
|  |  |  |  |  |  |
|  | **Cerebral Hemorrhage** | 79 | 2.537 | 34.8 |  |
|  |  |  |  |  |  |
|  |  |  |  |  |  |
|  | **Aspiration Pneumonia** | 78 | 2.505 | 37.3 |  |
|  |  |  |  |  |  |
|  |  |  |  |  |  |
|  | **Respiratory Failure** | 68 | 2.184 | 39.5 |  |
|  |  |  |  |  |  |
|  |  |  |  |  |  |
|  | **Hemorrhagic Transformation** | 67 | 2.152 | 41.7 |  |
|  |  |  |  |  |  |
|  |  |  |  |  |  |
|  | **Pulmonary Embolism** | 63 | 2.023 | 43.7 |  |
|  |  |  |  |  |  |
|  |  |  |  |  |  |
|  | **Atrial Fibrillation** | 56 | 1.798 | 45.5 |  |
|  |  |  |  |  |  |
|  |  |  |  |  |  |
|  | **Urinary Tract Infection** | 52 | 1.670 | 47.2 |  |
|  |  |  |  |  |  |
|  |  |  |  |  |  |
|  | **Pulmonary Edema** | 48 | 1.541 | 48.7 |  |
|  |  |  |  |  |  |
|  |  |  |  |  |  |
|  | **Cerebrovascular Disorder** | 47 | 1.509 | 50.2 |  |
|  |  |  |  |  |  |
|  |  |  |  |  |  |
|  | **Cardiac Arrest** | 46 | 1.477 | 51.7 |  |
|  |  |  |  |  |  |
|  |  |  |  |  |  |
|  | **Carotid Artery Disease** | 43 | 1.381 | 53.1 |  |
|  |  |  |  |  |  |
|  |  |  |  |  |  |
|  | **Sepsis** | 39 | 1.252 | 54.3 |  |
|  |  |  |  |  |  |
|  |  |  |  |  |  |
|  | **Angina Pectoris** | 37 | 1.188 | 55.5 |  |
|  |  |  |  |  |  |
|  |  |  |  |  |  |
|  | **Hypotension** | 35 | 1.124 | 56.6 |  |
|  |  |  |  |  |  |
|  |  |  |  |  |  |
|  | **Coma** | 34 | 1.092 | 57.7 |  |
|  |  |  |  |  |  |
|  |  |  |  |  |  |
|  | **Pyrexia** | 33 | 1.060 | 58.8 |  |
|  |  |  |  |  |  |
|  |  |  |  |  |  |
|  | **Renal Failure** | 31 | 0.996 | 59.8 |  |
|  |  |  |  |  |  |

|  | | | | | |
| --- | --- | --- | --- | --- | --- |
| **EXPECTED EVENTS (cont.)** | | | | | |
|  | | | | | |
|  | | | | | |
|  |  |  |  |  |  |
|  | **Serious Adverse Event (AE)** | **Number of Occurrences** | **% of Total SAE Occurrences** | **Cumulative % of SAE Occurrences** |  |
|  |  |  |  |  |  |
|  |  |  |  |  |  |
|  | **Headache** | 29 | 0.931 | 60.7 |  |
|  |  |  |  |  |  |
|  |  |  |  |  |  |
|  | **ICP increased** | 27 | 0.867 | 61.6 |  |
|  |  |  |  |  |  |
|  |  |  |  |  |  |
|  | **Respiratory Tract Infection** | 27 | 0.867 | 62.5 |  |
|  |  |  |  |  |  |
|  |  |  |  |  |  |
|  | **Bradycardia** | 26 | 0.835 | 63.3 |  |
|  |  |  |  |  |  |
|  |  |  |  |  |  |
|  | **Transient Ischemic Attack** | 25 | 0.803 | 64.1 |  |
|  |  |  |  |  |  |
|  |  |  |  |  |  |
|  | **Gastrointestinal Hemorrhage** | 24 | 0.771 | 64.9 |  |
|  |  |  |  |  |  |
|  |  |  |  |  |  |
|  | **Hypertension** | 24 | 0.771 | 65.6 |  |
|  |  |  |  |  |  |
|  |  |  |  |  |  |
|  | **Hemorrhagic Cerebral Infarction** | 22 | 0.706 | 66.3 |  |
|  |  |  |  |  |  |
|  |  |  |  |  |  |
|  | **Somnolence** | 22 | 0.706 | 67.1 |  |
|  |  |  |  |  |  |
|  |  |  |  |  |  |
|  | **Cerebral Incarceration** | 18 | 0.578 | 67.6 |  |
|  |  |  |  |  |  |
|  |  |  |  |  |  |
|  | **Deep Vein Thrombosis** | 18 | 0.578 | 68.2 |  |
|  |  |  |  |  |  |
|  |  |  |  |  |  |
|  | **Syncope** | 17 | 0.546 | 68.8 |  |
|  |  |  |  |  |  |
|  |  |  |  |  |  |
|  | **Depression** | 16 | 0.514 | 69.3 |  |
|  |  |  |  |  |  |
|  |  |  |  |  |  |
|  | **Hematuria** | 15 | 0.482 | 69.7 |  |
|  |  |  |  |  |  |
|  |  |  |  |  |  |
|  | **Vomiting** | 14 | 0.450 | 70.2 |  |
|  |  |  |  |  |  |
|  |  |  |  |  |  |
|  | **Cerebrovascular Accident** | 13 | 0.417 | 70.6 |  |
|  |  |  |  |  |  |
|  |  |  |  |  |  |
|  | **Convulsion** | 13 | 0.417 | 71.0 |  |
|  |  |  |  |  |  |
|  |  |  |  |  |  |
|  | **COPD** | 12 | 0.385 | 71.4 |  |
|  |  |  |  |  |  |
|  |  |  |  |  |  |
|  | **Dyspnea** | 12 | 0.385 | 71.8 |  |
|  |  |  |  |  |  |
|  |  |  |  |  |  |
|  | **Anemia** | 11 | 0.353 | 72.2 |  |
|  |  |  |  |  |  |
|  |  |  |  |  |  |
|  | **Aspiration** | 11 | 0.353 | 72.5 |  |
|  |  |  |  |  |  |
|  |  |  |  |  |  |
|  | **Bronchitis Acute** | 11 | 0.353 | 72.9 |  |
|  |  |  |  |  |  |
|  |  |  |  |  |  |
|  | **Cardiopulmonary Failure** | 11 | 0.353 | 73.2 |  |
|  |  |  |  |  |  |
|  | | | | | |

|  | | | | | |
| --- | --- | --- | --- | --- | --- |
| **EXPECTED EVENTS (cont.)** | | | | | |
|  | | | | | |
|  | | | | | |
|  |  |  |  |  |  |
|  | **Serious Adverse Event (AE)** | **Number of Occurrences** | **% of Total SAE Occurrences** | **Cumulative % of SAE Occurrences** |  |
|  |  |  |  |  |  |
|  |  |  |  |  |  |
|  | **Constipation** | 11 | 0.353 | 73.6 |  |
|  |  |  |  |  |  |
|  |  |  |  |  |  |
|  | **Epilepsy** | 11 | 0.353 | 73.9 |  |
|  |  |  |  |  |  |
|  |  |  |  |  |  |
|  | **Abdominal Tenderness** | 10 | 0.321 | 74.2 |  |
|  |  |  |  |  |  |
|  |  |  |  |  |  |
|  | **Dehydration** | 10 | 0.321 | 74.6 |  |
|  |  |  |  |  |  |
|  |  |  |  |  |  |
|  | **Ileus** | 10 | 0.321 | 74.9 |  |
|  |  |  |  |  |  |
|  |  |  |  |  |  |
|  | **Septic Shock** | 10 | 0.321 | 75.2 |  |
|  |  |  |  |  |  |
|  |  |  |  |  |  |
|  | **Colon Neoplasm** | 9 | 0.289 | 75.5 |  |
|  |  |  |  |  |  |
|  |  |  |  |  |  |
|  | **Rectal Hemorrhage** | 9 | 0.289 | 75.8 |  |
|  |  |  |  |  |  |
|  |  |  |  |  |  |
|  | **Tachycardia** | 9 | 0.289 | 76.1 |  |
|  |  |  |  |  |  |
|  |  |  |  |  |  |
|  | **Thrombosis** | 9 | 0.289 | 76.4 |  |
|  |  |  |  |  |  |
|  |  |  |  |  |  |
|  | **Ventricular Tachycardia** | 9 | 0.289 | 76.7 |  |
|  |  |  |  |  |  |
|  |  |  |  |  |  |
|  | **Arrhythmia** | 8 | 0.257 | 76.9 |  |
|  |  |  |  |  |  |
|  |  |  |  |  |  |
|  | **Coronary Artery Disease** | 8 | 0.257 | 77.2 |  |
|  |  |  |  |  |  |
|  |  |  |  |  |  |
|  | **Multi-organ Failure** | 8 | 0.257 | 77.4 |  |
|  |  |  |  |  |  |
|  |  |  |  |  |  |
|  | **Neurological Symptom** | 8 | 0.257 | 77.7 |  |
|  |  |  |  |  |  |
|  |  |  |  |  |  |
|  | **Respiratory Arrest** | 8 | 0.257 | 77.9 |  |
|  |  |  |  |  |  |
|  |  |  |  |  |  |
|  | **Shock** | 8 | 0.257 | 78.2 |  |
|  |  |  |  |  |  |
|  |  |  |  |  |  |
|  | **Urosepsis** | 8 | 0.257 | 78.5 |  |
|  |  |  |  |  |  |
|  |  |  |  |  |  |
|  | **Cardiogenic Shock** | 7 | 0.225 | 78.7 |  |
|  |  |  |  |  |  |
|  |  |  |  |  |  |
|  | **Hip Fracture** | 7 | 0.225 | 78.9 |  |
|  |  |  |  |  |  |
|  |  |  |  |  |  |
|  | **Melena** | 7 | 0.225 | 79.1 |  |
|  |  |  |  |  |  |
|  |  |  |  |  |  |
|  | **Nausea** | 7 | 0.225 | 79.4 |  |
|  |  |  |  |  |  |
|  | | | | | |

|  |
| --- |
|  |
|  |
|  |
|  |
| **PREDEFINED EVENTS OF SPECIAL INTEREST** |
|  |
|  |
|  |
| The following is a list of predefined events of special interest:   - Stroke - Dementia - Atrial fibrillation - Myocardial infarction - Acute coronary syndrome (ACS) - Suspected drug rash - Renal failure (defined as eGFR falling to <60 mls/min) - Cardiac failure (hospitalisation with) - Allopurinol hypersensitivity reaction including Stevens Johnson syndrome, toxic epidermal necrolysis - Blood and lymphatic system problems - eosinophilia (>0.45*10^9) / neutropenia (<1.5 *10^9) / thrombocytopenia (<50*10^9) or anaemia (haemoglobin <10g/dl) - Hepatic dysfunction (defined as serum bilirubin, AST or ALT greater than three times upper limit of normal (ULN)).   N.B, participants will be asked whether they have suffered gout at all visits. This will not be a study outcome measure. |
|  |

|  |
| --- |

1. Sacco RL, Diener HC, Yusuf S, Cotton D, Ounpuu S, Lawton WA, et al. Aspirin and extended-release dipyridamole versus clopidogrel for recurrent stroke. New England Journal of Medicine 2008 Sep 18;359(12):1238-51. [↑](#endnote-ref-1)
2. Pendlebury ST, Rothwell PM. Prevalence, incidence, and factors associated with pre-stroke and post-stroke dementia: a systematic review and meta-analysis.Lancet Neurology 2009 Nov;8(11):1006-18. [↑](#endnote-ref-2)
3. Serrano S, Domingo J, Rodriguez-Garcia E, Castro MD, del ST. Frequency of cognitive impairment without dementia in patients with stroke: a two-year follow-up study. Stroke 2007 Jan;38(1):105-10. [↑](#endnote-ref-3)
4. Pollock A, St George B, Fenton M, Firkins L. Top ten research priorities relating to life after stroke. The Lancet Neurology 2012 Mar;11(3):209. [↑](#endnote-ref-4)
5. Debette S, Markus HS. The clinical importance of white matter hyperintensities on brain magnetic resonance imaging: systematic review and meta-analysis. BMJ 2010;341:c3666. [↑](#endnote-ref-5)
6. Fu JH, Lu CZ, Hong Z, Dong Q, Luo Y, Wong KS. Extent of white matter lesions is related to acute subcortical infarcts and predicts further stroke risk in patients with first ever ischaemic stroke. Journal of Neurology, Neurosurgery & Psychiatry 2005 Jun;76(6):793-6. [↑](#endnote-ref-6)
7. Weber R, Weimar C, Blatchford J, Hermansson K, Wanke I, M+Âller-Hartmann C, et al. Telmisartan on Top of Antihypertensive Treatment Does Not Prevent Progression of Cerebral White Matter Lesions in the Prevention Regimen for Effectively Avoiding Second Strokes (PRoFESS) MRI Sub-study. Stroke 2012 Sep 1;43(9):2336-42. [↑](#endnote-ref-7)
8. Oksala NK, Oksala A, Pohjasvaara T, Vataja R, Kaste M, Karhunen PJ, et al. Age related white matter changes predict stroke death in long term follow-up. Journal of Neurology, Neurosurgery & Psychiatry 2009 Jul;80(7):762-6. [↑](#endnote-ref-8)
9. van Dijk EJ, Prins ND, Vrooman HA, Hofman A, Koudstaal PJ, Breteler MMB. Progression of Cerebral Small Vessel Disease in Relation to Risk Factors and Cognitive Consequences. Stroke 2008 Oct 1;39(10):2712-9. [↑](#endnote-ref-9)
10. Schmidt R, Berghold A, Jokinen H, Gouw AA, van der Flier WM, Barkhof F, et al. White Matter Lesion Progression in LADIS. Stroke 2012 Oct 1;43(10):2643-7. [↑](#endnote-ref-10)
11. Sierra C, de la Sierra A, Pare JC, Gomez-Angelats E, Coca A. Correlation between silent cerebral white matter lesions and left ventricular mass and geometry in essential hypertension. American Journal of Hypertension 2002 Jun;15(6):507-12. [↑](#endnote-ref-11)
12. Dufouil C, Chalmers J, Coskun O, Besan+ºon V+, Bousser MG, Guillon P, et al. Effects of Blood Pressure Lowering on Cerebral White Matter Hyperintensities in Patients With Stroke. Circulation 2005 Sep 13;112(11):1644-50. [↑](#endnote-ref-12)
13. Vlek AL, Visseren FL, Kappelle LJ, Witkamp TD, Vincken KL, Mali WP, et al. Blood pressure and white matter lesions in patients with vascular disease: the SMART-MR study. Current Neurovascular Research 2009 Aug;6(3):155-62. [↑](#endnote-ref-13)
14. Selvetella G, Notte A, Maffei A, Calistri V, Scamardella V, Frati G, et al. Left ventricular hypertrophy is associated with asymptomatic cerebral damage in hypertensive patients. Stroke 2003 Jul;34(7):1766-70. [↑](#endnote-ref-14)
15. Fox ER, Taylor HA, Jr., Benjamin EJ, Ding J, Liebson PR, Arnett D, et al. Left ventricular mass indexed to height and prevalent MRI cerebrovascular disease in an African American cohort: the Atherosclerotic Risk in Communities study. Stroke 2005 Mar;36(3):546-50. [↑](#endnote-ref-15)
16. van Elderen SG, Brandts A, Westenberg JJ, van der Grond J, Tamsma JT, van Buchem MA, et al. Aortic stiffness is associated with cardiac function and cerebral small vessel disease in patients with type 1 diabetes mellitus: assessment by magnetic resonance imaging. European Radiology 2010 May;20(5):1132-8. [↑](#endnote-ref-16)
17. Boyd AC, McKay T, Nasibi S, Richards DAB, Thomas L. Left ventricular mass predicts left atrial appendage thrombus in persistent atrial fibrillation. European Heart Journal GÇô Cardiovascular Imaging 2012 Jul 24. [↑](#endnote-ref-17)
18. Costanzo P, Savarese G, Rosano G, Musella F, Casaretti L, Vassallo E, et al. Left ventricular hypertrophy reduction and clinical events. A meta-regression analysis of 14 studies in 12,809 hypertensive patients. International Journal of Cardiology(0). [↑](#endnote-ref-18)
19. Devereux RB, Dahlof B, Gerdts E, Boman K, Nieminen MS, Papademetriou V, et al. Regression of Hypertensive Left Ventricular Hypertrophy by Losartan Compared With Atenolol. Circulation 2004 Sep 14;110(11):1456-62. [↑](#endnote-ref-19)
20. Dawson J, Quinn T, Walters M. Uric acid reduction: a new paradigm in the management of cardiovascular risk?. Current Medicinal Chemistry 2007;14(17):1879-86. [↑](#endnote-ref-20)
21. Weir CJ, Muir SW, Walters MR, Lees KR. Serum urate as an independent predictor of poor outcome and future vascular events after acute stroke. Stroke 2003 Aug;34(8):1951-6. [↑](#endnote-ref-21)
22. Dawson J, Lees KR, Weir CJ, Quinn T, Ali M, Hennerici MG, et al. Baseline serum urate and 90-day functional outcomes following acute ischemic stroke. Cerebrovascular Diseases 2009;28(2):202-3. [↑](#endnote-ref-22)
23. Vannorsdall TD, Jinnah HA, Gordon B, Kraut M, Schretlen DJ. Cerebral ischemia mediates the effect of serum uric acid on cognitive function. Stroke 2008 Dec;39(12):3418-20. [↑](#endnote-ref-23)
24. Dawson J, Quinn TJ, Harrow C, Lees KR, Walters MR. The effect of allopurinol on the cerebral vasculature of patients with subcortical stroke; a randomized trial. British Journal of Clinical Pharmacology 2009 Nov;68(5):662-8. [↑](#endnote-ref-24)
25. Muir SW, Harrow C, Dawson J, Lees KR, Weir CJ, Sattar N, et al. Allopurinol use yields potentially beneficial effects on inflammatory indices in those with recent ischemic stroke: a randomized, double-blind, placebo-controlled trial. Stroke 2008 Dec;39(12):3303-7. [↑](#endnote-ref-25)
26. George J, Carr E, Davies J, Belch JJ, Struthers A. High-dose allopurinol improves endothelial function by profoundly reducing vascular oxidative stress and not by lowering uric acid. Circulation 2006 Dec 5;114(23):2508-16. [↑](#endnote-ref-26)
27. Dawson J, Quinn T, Harrow C, Lees KR, Weir CJ, Cleland SJ, et al. Allopurinol and Nitric Oxide Activity in the Cerebral Circulation of Those With Diabetes: A randomized trial. Diabetes Care 2009 Jan;32(1):135-7. [↑](#endnote-ref-27)
28. Kao MP, Ang DS, Gandy SJ, Nadir MA, Houston JG, Lang CC, et al. Allopurinol benefits left ventricular mass and endothelial dysfunction in chronic kidney disease. Journal of the American Society of Nephrology 2011 Jul;22(7):1382-9. [↑](#endnote-ref-28)
29. Noman A, Ang DS, Ogston S, Lang CC, Struthers AD. Effect of high-dose allopurinol on exercise in patients with chronic stable angina: a randomised, placebo controlled crossover trial. Lancet 2010 Jun 19;375(9732):2161-7. [↑](#endnote-ref-29)
30. Khan F, George J, Wong K, McSwiggan S, Struthers AD, Belch JJ. Allopurinol treatment reduces arterial wave reflection in stroke survivors. Cardiovasc Ther 2008;26(4):247-52. [↑](#endnote-ref-30)
31. Feig DI, Soletsky B, Johnson RJ. Effect of allopurinol on blood pressure of adolescents with newly diagnosed essential hypertension: a randomized trial. JAMA 2008 Aug 27;300(8):924-32. [↑](#endnote-ref-31)
32. Agarwal V, Hans N, Messerli FH. Effect of Allopurinol on Blood Pressure: A Systematic Review and Meta-Analysis. The Journal of Clinical Hypertension 2012;no. [↑](#endnote-ref-32)
33. Higgins P, Dawson J, Lees KR, McArthur K, Quinn TJ, Walters MR. Xanthine Oxidase Inhibition For The Treatment Of Cardiovascular Disease: A Systematic Review and Meta-Analysis. Cardiovasc Ther 2012;30(4):217-26. [↑](#endnote-ref-33)
34. Emmerson, B. T. "Drug therapy - The management of gout." New England Journal of Medicine 334.7 (1996): 445-51. [↑](#endnote-ref-34)
35. Roujeau JC, Kelly JP, Naldi L, *et al.*Medication use and the risk of Stevens-Johnson syndrome or toxic epidermal necrolysis. N Engl J Med, 1995;333:1600–1607 [↑](#endnote-ref-35)
36. Hachinski V, Iadecola C, Petersen RC, Breteler MM, Nyenhuis DL, Black SE, et al. National Institute of Neurological Disorders and StrokeGÇôCanadian Stroke Network Vascular Cognitive Impairment Harmonization Standards. Stroke 2006 Sep 1;37(9):2220-41. [↑](#endnote-ref-36)
37. [Anon]: Excess of Ampicillin Rashes Associated with Allopurinol Or Hyperuricemia. New England Journal of Medicine 286:505-&, 1972 [↑](#endnote-ref-37)
38. Hare JM, Mangal B, Brown J et al. Impact of oxypurinol in patients with symptomatic heart failure. J Am Coll Cardiol 2008;51:2301-2309. [↑](#endnote-ref-38)
39. Hesse K, Fulton RL, Abdul-Rahim AH, Lees KR. Characteristic Adverse Events and Their Incidence Among Patients Participating in Acute Ischaemic Stroke Trails. Stroke 2014;45:2677-2682 [↑](#endnote-ref-39)
40. Migrino RQ, Bowers M, Harmann L, Prost R, LaDisa JF. Carotid plaque regression following 6- month statin therapy assessed by 3T cardiovascular magnetic resonance: comparison with ultrasound intima media thickness. JCMR 2011;13:37 doi:10.1186/1532-429X-13-37 [↑](#endnote-ref-40)
